# Microcomputed tomography analysis of curved root canal preparation when coronal flaring and glide path files used with heat-treated nickel titanium rotary files

**DOI:** 10.1371/journal.pone.0299896

**Published:** 2024-04-03

**Authors:** Ammar AbuMostafa, Mohammed M. Alrefaie, Nedal Abu-Mostafa, Fahda N. Algahtani

**Affiliations:** 1 Department of Restorative Dentistry, College of Dentistry, Riyadh Elm University, Riyadh, Kingdom of Saudi Arabia; 2 Department of Restorative Dentistry, Ministry of Health, Riyadh Third Cluster, Riyadh, Kingdom of Saudi Arabia; 3 Department of Oral and Maxillofacial Surgery and Diagnostic Science, College of Dentistry, Riyadh Elm University, Riyadh, Kingdom of Saudi Arabia; 4 Department of Clinical Dental Sciences, College of Dentistry, Princess Nourah Bint Abdulrahman University, Riyadh, Saudi Arabia; University of Puthisastra, CAMBODIA

## Abstract

The objective was to evaluate the effect of glide path and coronal flaring on the dentin volume removal and percentage of touched walls in curved canals using two heat-treated rotary files. The mesiobuccal canal of forty-eight, randomly selected, extracted mandibular molars was divided into two groups of 24 each, according to the type of instrument used (RACE EVO and EdgeSequel rotary files). Each group was further divided into three subgroups; Group (A): Control using one file shaped to 04/30, Group (B) with a glide path (EdgeGlidePath (EGP)), and Group (C): with a glide path and coronal flaring (EGP and EdgeTaper Platinum (ETP) SX file respectively). The root canals were then instrumented using the assigned instruments. The assessment was carried out using micro-CT. The comparison of the mean values of the tested groups about dentin volume removal and percentage of untouched walls did not reach statistical significance (p<0.05). Glide path and coronal flaring had an insignificant effect on the dentin volume removal and percentage of untouched walls in curved canals.

## Introduction

Endodontic infections are bacteriogenic, therefore adequate disinfection of root canal systems is necessary for successful treatment outcomes [1]. Root canal anatomy is a complicated system and complete eradication of bacteria is not possible [1]. Shaping of root canals is a key step to allow disinfectant to clean the end of the root [1]. Therapy will result in the healing of the periapical tissue and the preservation of the dental structure [1].

The complicity of the root canal system was addressed in the literature [1]. Root canal treatment is difficult for undergraduate students and general dentists, especially in the molar region [1–3]. The mishaps are mainly located in this region for many different reasons [3]. One of the main reasons is the curvature of the root in the molar region particularly in the mesial root of upper and lower molars [3]. Facing this challenge, root canal preparation techniques were modernized over the years, resulting in several alternative techniques available today [4].

The main traditional techniques such as the anti-curvature and the crown-down technique indicated that the coronal portion of the curved canal has to be straightened using peeso reamers or gates glidden (GG) [5, 6]. These techniques aimed to reduce the number of curvatures in curved canals especially in the posterior region [5, 6]. Reducing the number of curvatures will reduce the friction between the file and the coronal canal walls, which will allow the file to freely and actively engage with the walls in the apical region [5, 6]. This technique had enormous success because it improved the file’s ability to reach the end of the root and reduce the risk of file fracture [5, 6].

Nowadays, motor-driven files are used and this technique was modified [7–9]. The highly tapered orifice opener is used to open the canal in the coronal third followed by small size rotary file to create a smooth glide path to the end of the curved root canal system [7–9]. Finally, the canal will be prepared to the final shape to allow adequate cleaning of root canal space and root obturation [7–9]. However, the current development in root file manufacturing and processing techniques in addition to current dynamic irrigation techniques has even challenged this concept [10].

The modern heat-treated nickel titanium files are believed to have exceptional flexibility, cantering ability, and cyclic fatigue resistance [11]. For example, the RACE EVO (RE) files (FKG, La Chaux-de-Fonds, Switzerland) manufacturer claimed that in addition to the previously mentioned properties, these heat-treated files have high cutting efficiency and operate at high speed improving their overall efficiency. Another example is EdgeSequel (ES) files (EdgeEndo, New Mexico, USA.) which have unique designs that improve their flexibility and make them an excellent choice for curved root canal systems. Since both of these files are synthesized to have high flexibility and the ability to endure challenging curved root canal systems, the need for coronal flaring before their use is questioned [10]. Moreover, the benefits of using glide path rotary files before these rotary files need to be examined.

The efficiency of cutting plays a pivotal role in the overall performance of rotary files. Root canal files (RE) are characterized by a rounded triangular cross-section, whereas endodontic files (ES) possess a triangular cross-section. Notably, files with a triangular cross-section are deemed sharp files and are recognized for their superior cutting efficiency [12, 13]. However, while the literature acknowledges the cutting efficiency of triangular cross-section files, there is limited exploration into their application as single rotary files. Accordingly, this study intended to explore the feasibility of using these files as single rotary files. Moreover, the investigation extended to assess any impact of utilizing glide path files and orifice openers with triangular cross-sections in conjunction with the rotary files.

Excessive removal of dentin during coronal flaring may result in perforation and treatment failure [10]. Additionally, excessive removal of dentin at the cervical region will weaken the root structure rendering it susceptible to fracture [10]. The glide path rotary files were advised to use especially in curved root canals to reduce procedural errors such as ledging and file breakage [14, 15]. The advantage of their use in addition to the modern highly flexible heat-treated NiTi rotary files is questionable [14, 15]. Moreover, dynamic irrigation is intended to improve irrigation penetrative and lubricant ability reducing the need for excessive flaring or sequential use of rotary files [4].

One of the main trends in endodontics is to reduce the amount of tooth structure removed in endodontic access and root canal shaping [10]. Because these traditional access and shaping techniques became unnecessary for predictable disinfection of the canals in light of the modern advances in magnification and technology in shaping and irrigation [4, 10]. Thus the effect of coronal flaring and glide path in the amount of dentin volume removal and percentage of touched walls in curved canals need to be evaluated. Moreover. RE and ES were not compared in terms of the amount of dentin volume removal and percentage of touched walls. Therefore, the study aimed to compare the coronal pre-flaring and glide path in addition to RE and ES in terms of dentin volume removal and percentage of touched walls in curved root canal systems. The Null hypothesis was that there would be no significant difference in the dentin volume removal and percentage of touched walls in curved canals among the tested groups.

## Material and methods

### Ethical approvals

The research was registered in the Research Centre of Riyadh Elm University and received ethical approval from the Institutional Review Board (IRB) (approval number: FPGRP/2021/598/533/563). Consent forms were not obtained as this was a laboratory-based study involving no human subjects. Additionally, the teeth used in this study were extracted from patients undergoing orthodontic or periodontal treatment, not specifically for this study. These teeth were collected from the university’s dental clinics during December 2021 and January 2022. The extracted teeth were cleaned and disinfected in 1% Sodium hypochlorite (NaOCl) (Prime dental products, India). Then they were stored in a glass bottle containing 0.1% hyaluronate solution (Synojoint, Arthrex, Germany).

### Sample power calculation

The Sample power was calculated using the G-Power sample power calculator (Universitat-Kiel, Kiel, Germany). Using 5% as margin of error, 95% confidence interval, and power of 80%, a total sample size of 48, with eight samples per group, was determined.

### Specimen selection

The mesial root of the randomly selected extracted teeth was examined visually and radiographically. The visual examination was carried out using 10x magnification power under the dental microscope. While the radiographic examination was carried out using the Micro-CT (Bruker SkyScan, Kontich, Belgium) to confirm the suitability of teeth for the objectives of the study. The inclusion criteria were the following: permanent sound teeth, Vertucci type IV root canal anatomy in the mesial root (two separate mesial canals), and the range of canal curvature between 20° - 40° [16, 17]. On the other hand, the following teeth were excluded: those with more than one apical foramen, teeth with resorption, teeth with previous endodontic treatment, and cracked or fractured roots.

### Grouping

**Fig 1:**
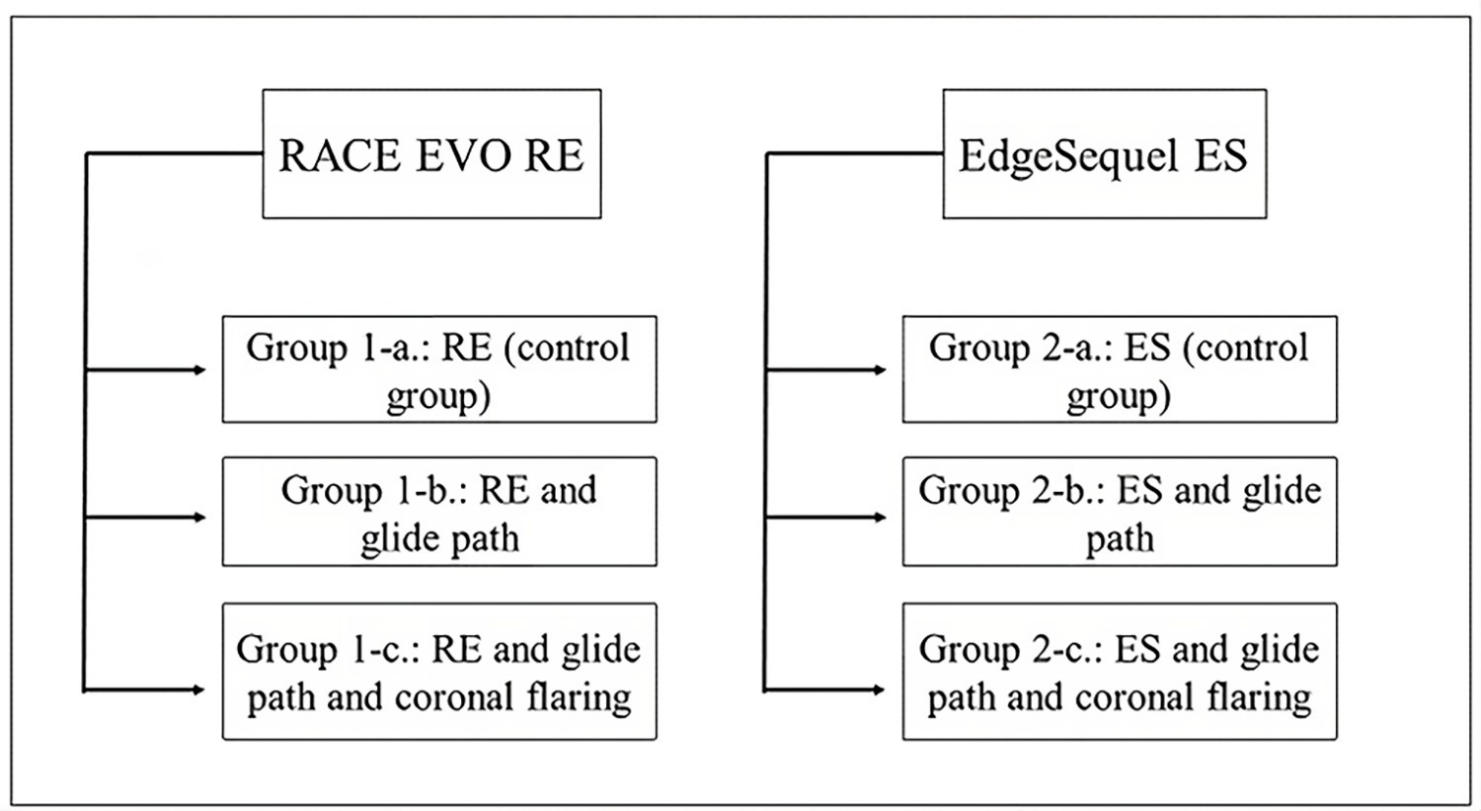
The main two groups and the six subgroups used in the experiment.

The forty-eight molars were allocated into two main groups (N = 24) based on the type of rotary file used for shaping the MB canal. Then each group was subdivided into three subgroups (n = 8) based on the shaping technique as it is shown in Fig 1. The total number of subgroups was six.

### Specimen preparation

#### Access opening and working length

An endodontic resident in his final year of clinical training accessed the extracted molars under the dental microscope (Zumax Medical Co. Jiangsu Province, China). Two sets of burs were used for the access cavity preparation. The crown initially was accessed with a round bur size 4 (Brasseler, USA) to reach the roof of the pulp chamber and remove any dentin overhang in the pulp chamber. Then the access was refined using End Z bur (Dentsply Maillefer, Switzerland). The initial file was a #10 K-file (Dentsply Maillefer, Switzerland) and it was used to negotiate the MB canal until the end of this file was visualized at the apical foramen. At this point, the working length was determined by deducting 1 mm from the total length of the canal. The canal was then shaped with #15 K-file (Dentsply Maillefer, Switzerland) to the previously established working length. The irrigation was carried on consistently during the procedure using 2.5% Sodium hypochlorite NaOCl (Prime dental products, India) solution.

#### Chemo-mechanical preparation

The same endodontic resident with knowledge and experience of all systems used for the study performed the shaping procedure under dental microscope. The shaping techniques used for coronal flaring, glide path preparation, and final shaping are summarized in Table 1. The endodontic engine was adjusted to the desired manufacturer-recommended settings in continuous rotation motion (X-Smart, Dentsply Maillefer, Switzerland). The lubricating agent and the irrigation solution were used constantly during the shaping procedure and in between the files. The lubricant was 15% ethylenediaminetetracetic acid (EDTA) cream RC-Prep (Premier, Philadelphia, United States of America) and the irrigant was 2.5% NaOCl solution. The total volume of irrigation material used during working length determination and shaping was 15 mL NaOCL and the shaping procedure consumed 2 to 2:30 minutes. The canal was dried with size 30 Edge paper point with 04 taper (EdgeEndo, New Mexico, USA.) prior Micro-CT scanning.

**Table 1:** The rotary files used and the followed manufacturer instructions [18].

The shaping procedure	The file type (size/taper)	The manufacturer	The speed and torque	The shaping technique
**Coronal flaring**	EdgeTaper Platinum (ETP) SX file (25/12)	EdgeEndo, New Mexico, USA.	350 ^RPM and 3 ^^NCM	The file used in gentle picking motion with lubricant in the coronal third (less than 7 mm) to create straight line access after measuring the working length. The canal was shaped with ETP before measuring the working length in group c.
**Glide path**	EdgeGlidePath (EGP) file (19/variable taper)	EdgeEndo, New Mexico, USA.	400 RPM and 3 NCM	The rotary file used gently and gradually in picking motion in the presence of lubricant until it reaches the full working length. The canal was shaped with EGP after it was shaped with size 15 K-file in group b and c.
**Group1 mechanical preparation**	*RE (30/04)	FKG, La Chaux-de-Fonds, Switzerland	450 RPM and 3 NCM	The rotary file used gently and gradually in picking motion in the presence of lubricant until it reaches the full working length. The canal was shaped to 30/04 in all groups.
**Group2 mechanical preparation**	**ES (30/04)	EdgeEndo, New Mexico, USA.	800 RPM and 1.5 NCM	

*RE: RACE EVO

**ES: RACE EVO

^RPM: Round Per Minute

^^NCM: Newton Centimeters.

### Micro-CT analysis

All the teeth had pre- and post-treatment scans using the Bruker SkyScan 1172 high-resolution micro-CT (Bruker SkyScan, Kontich, Belgium). The teeth were mounted individually in polyethylene plastic containers to allow standardization of their position while being secured with polyvinyl siloxane on the micro-stage of the specimen chamber in the micro-CT machine (Bruker SkyScan, Kontich, Belgium).

The scanner configuration used was 95 kV voltage, 104 μA anode current, 158 ms exposure time, 25 μm image pixel size, Al 0.5 mm, 0.3 rotation step for 360° angle, frame averaging of 4 for improved signal-to-noise ratio and random movement of 8 minimize ring artifacts. A flat-field correction was performed before the scanning procedure to correct variations in the camera pixel sensitivity.

After the scanning, a reconstruction of the projected images was performed using N-Recon, program version 1.6.9.4 (Bruker Skyscan, Belgium) to produce a reconstructed cross-sectional image. Numerical parameters needed to establish the best image results were checked and adjusted. A ring artifact reduction of 5 for non-uniformity of the background image taken by the x-ray camera; 25% beam hardening compensation to prevent the specimen from appearing artificially denser at or near its surface, and less dense at its central parts; and a smoothing of 2 using Gaussian kernel were applied. A 16-bit TIF file format was the choice for saving the images because of the variety of densities comprising the specimen.

Reconstructed Pre- and Post-images were loaded to the Dataviewer program version 1.5.6.2 (Bruker Skyscan, Belgium) Software for 3D co-registration. A registration data set was then saved and loaded in the CTAn software for imaging and analyzing selectively, binarising, and quantifying pre- and post-scan root canal instrumentation. Finally, CTVol 2.3.2.0 (Bruker Skyscan, Belgium) was used for 3D visualization and production of color-coded images of the samples.

### Calculate dentin volume removal and touched walls

The amount of volume removed from the root canal surface area was calculated by subtracting the values for the treated canals from those recorded for the untreated counterparts. However, the percentage of untouched canal area was calculated by the number of static voxels (voxels present in the same position on the canal surface before and after instrumentation divided by the total number of voxels present on the canal surface ^14^, according to the following formula:

Thepercentageofuntouchedcanalarea=numberofstaticvoxels×100/totalnumberofsurfacevoxels

### Statistical analysis

Descriptive statistics were presented using mean and standard deviation. The comparison between dentin volume removal and touching ability among the performed root canal instrumentation was analyzed using the One-way Analysis of Variance (ANOVA). A p-value cut-off point of 0.05 at 95% CI was used to determine statistical significance. All data analyses were performed using Statistical Packages for Software Sciences (SPSS) version 21 Armonk, New York, IBM Corporation.

## Results

The comparison of the mean values of tested groups in terms of dentin volume removal is not statistically significant as shown in Table 2. The same table also shows that similar finding is observable when the subgroups in RE and SE were compared separately.

**Table 2:** The comparison of dentin volume removal within the subgroups of RE and ES individually and between the overall subgroups.

The subgroups for group 1	Dentin volume removal (mm^3^) Mean ± SD	F-test. P-value ^§^	The subgroups for group 2	Dentin volume removal (mm^3^) Mean ± SD	F-test. P-value ^§^
**RE** *	0.88 ± 0.21	2.454; 0.110	**ES** **	1.03 ± 0.26	0.603; 0.557
RE* **with glide path**	0.98 ± 0.30		ES** **with glide path**	1.07 ± 0.43	
RE* **with glide path and coronal flaring**	1.04 ± 0.23		ES** **with glide path and coronal flaring**	1.37 ± 0.27	
**F-test.** **P-value** ^**§**^	1.175; 0.338				

^§^ P-value was calculated using the One-Way ANOVA test

* RE: Race Evo

** ES: EdgeSequel.

**Fig 2:**
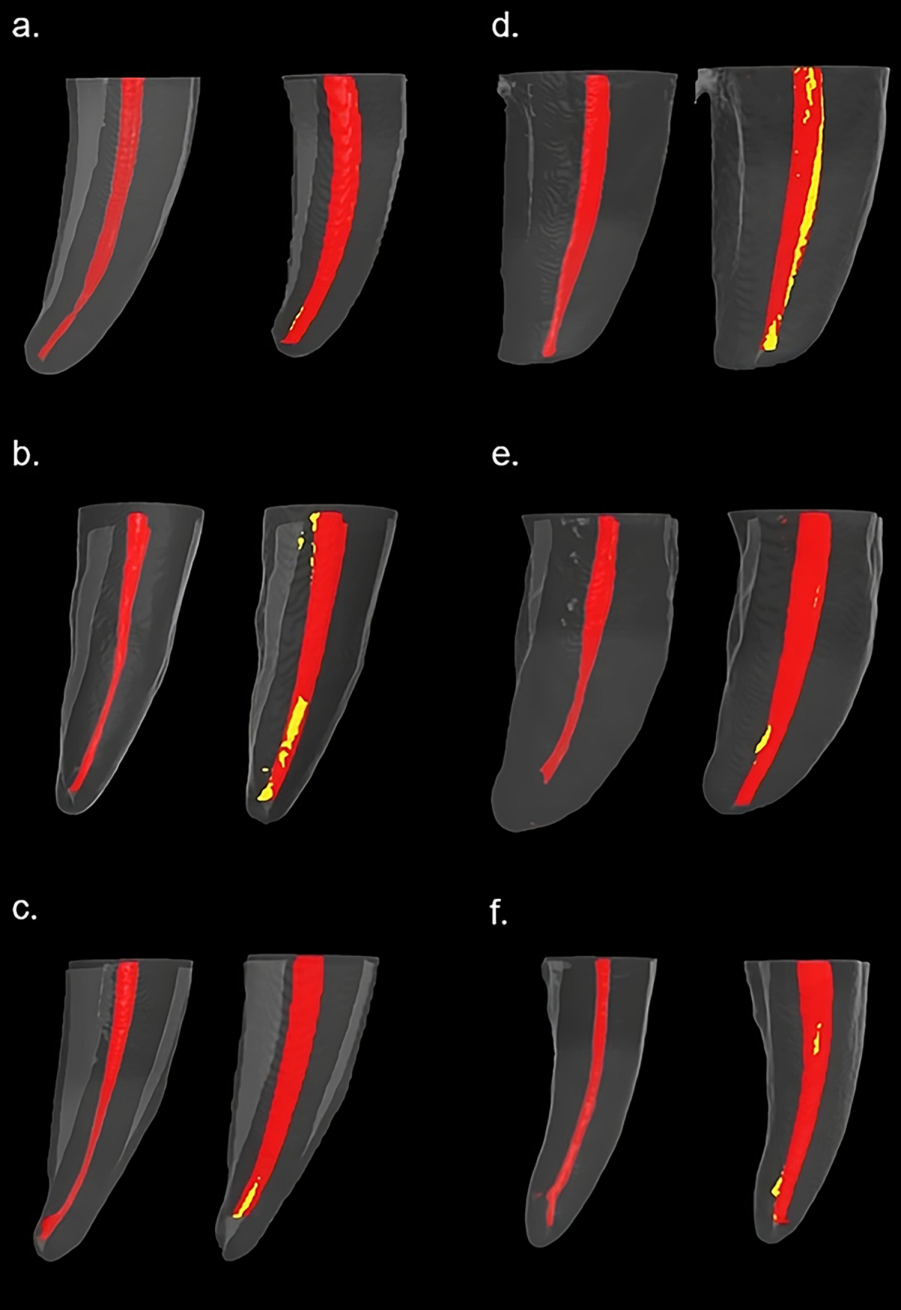
Three-dimensional models of the mesial root of lower mandibular molars that shows root canal anatomy before and after preparation. The red color indicates touched surfaces and the yellow indicates untouched surfaces in post-preparation configuration. (a, b, and c images) represent groups 1-a, 1-b, 1-c respectively using RE rotary files, (d, e, f images) 2-a, 2-b, 2-c respectively using ES rotary files represent group 2.

When mean values of the percentage of untouched walls are compared, there is no statistical difference between the overall subgroups and between the individual subgroups of the two main groups of RE and ES as shown in Table 3. Fig 2 shows the three-dimensional model of one of the subgroups and the visual appearance of the touched and untouched walls.

**Table 3:** The comparison of the percentage of untouched walls within the subgroups of RE and ES individually and between the overall subgroups.

The subgroups for group 1	% Of Untouched Walls	F-test.	The subgroups for group 2	% Of Untouched Walls	F-test.
	Mean ± SD	P-value ^§^		Mean ± SD	P-value ^§^
**RE** *	18.2 ± 3.52	1.766; 0.201	**ES** **	14.4 ± 3.51	0.059; 0.943
RE* **with glide path**	17.9 ± 3.89		ES** **with glide path**	14.6 ± 5.45	
RE* **with glide path and coronal flaring**	17.5 ± 5.91		ES** **with glide path and coronal flaring**	13.1 ± 4.37	
**F-test.** **P-value** ^**§**^	1.589; 0.184				

^§^ P-value has been calculated using the One-Way ANOVA test

* RE: Race Evo

** ES: EdgeSequel.

## Discussion

The present study evaluated the effect of glide path and coronal flaring on the dentin volume removal and the percentage of touched walls in curved canals of two NiTi instruments, RE and ES, using micro-computerized technology (micro-CT). No significant difference among the tested groups was found. Thus, the null hypothesis was accepted.

Curved root canals are a challenging experience for dental practitioners, and treatment mishaps are often common in these canals [2, 3]. The root canal angle and radius of curvature are one of the main factors considered when evaluating the difficulty of root canal treatment RCT [8]. One of the main recommended techniques to shape curved root canal anatomy is to follow the traditional concept of the anti-curvature technique and crown-down technique [5, 6]. This technique requires the clinicians to establish straight-line access to the mid-root to allow free access to the curvature in the coronal third [5, 6]. A further recommendation was to use small K-files in sequence to the root terminus to create a glide path for larger rotary NiTi files [8, 14, 15]. The glide path is a crucial step to prevent blockage of the curved root canal system. Since motor-driven rotary NiTi files are more efficient in creating the glide path, in comparison to manual K files, small-sized glide path rotary files were developed [8, 14, 15]. These files can be used after size 10 K files and before the use of rotary file systems [8, 14, 15].

The up-to-date thermal processing of NiTi files has improved their centering ability and cyclic fatigue resistance in curved canals [4, 11]. Moreover, dynamic irrigation has improved irrigants’ ability to reach the end of the root canal system [4]. Therefore, the endodontic practice gradually shifted to follow more conservative root canal preparation using small taper shaping files [19, 20]. A traditional concept such as the anti-curvature technique/crown-down using modern NiTi rotary files such as orifice openers in curved root canals has been challenged [19]. Although this technique has been successfully employed over the past years to reduce the incidence of stripping perforation, ledging, and file separation in curved root canal systems, this technique could also risk weakening the tooth structure at the cervical region resulting in early tooth loss as a result of a crack or vertical root fracture [19]. Clinicians often find themselves in a difficult spot in choosing whether to conserve the tooth structure in a curved root canal system or to improve the efficiency and predictability of shaping curved root apex using an orifice opener [19].

When orifice openers were compared to the Gates Glidden GG that was used earlier for coronal flaring, investigators found that GG drills removed substantially more dentin [21]. Unlike orifice openers that have the same geometry as the root canal, the football shape of GG drills may easily result in the perforation of mandibular molars [21]. The dentin volume reduction, after using the ETP orifice opener when combined with 04 tapered heat-treated rotary files, was not particularly studied before. The study found that coronal flaring using an SX orifice opener did not increase the removal of dentin volume in the coronal third. The ProTaper Gold orifice opener removed less amount of dentin in MB2 when it was compared to 25/04 and 30/06 tapered heat-treated rotary files [22]. Interestingly, one systemic review addressed the lack of evidence that associates coronal flaring with dentin micro-cracks formation [14]. Moreover, the review suggested that coronal flaring improves the accuracy of working length determination [14].

The same systemic review examined the role of glide path files in root canal shaping [14]. The glide path shaping was important for maintaining the root canal anatomy, however, in this review, they did not specify one particular file system or motion [14]. There are different types of rotary and reciprocating files that are used for glide path preparation and their performance can differ significantly [22]. For example, the ProGlider PG glide path file when used with ProTaper Next PN, appeared to reduce procedural mishaps such as transportation in curved canals, compared to the preparation of PN alone or PathFile PF and PN [23]. On the other hand, when the glide path was created by PF, it maintained the root canal anatomy compared to canals shaped with reciprocating WaveOne WO primary [24]. All these studies addressed the transportation and preservation of root canal anatomy. However, the focus of this study was the dentin volume removal that appeared to be not affected by the use of SX glide path files in both rotary file systems.

Many factors could influence the results of this study such as the metallurgy of the NiTi files, their sizes, and their taper. The NiTi files used in the current study are all thermally-treated and have controlled memory, while the WO file tested was mainly austenitic at room temperature [24]. The controlled memory improves the NiTi file-centering abilities which could reduce the need for glide path files [11]. Moreover, the final shape of root canal preparation in this study was 30/04 which could have similar dimensions to the SX orifice opener and could potentially mask the effect of glide path preparation.

Instrumentation with RE and ES left 17.86% and 14.03% of the canal walls untouched, respectively. An inverse relationship was found between the percentage of untouched walls and dentin volume removal, suggesting that the more dentin volume removal, the less percentage of untouched walls. Zhang et al. found that the percentage of untouched walls in the second mesiobuccal canals in maxillary first molars varied between 13.06% and 22.67% [25]. This could be explained by the fact that mesiobuccal canals in mandibular first molars used in this study, and maxillary molars that used in their study, are small and round. On the other hand, another study found that the percentage of untouched walls after instrumentation by XP endo Shaper and TRUShape of maxillary premolars was 47.26% and 48.18%, respectively [26]. The authors attributed this high percentage of untouched walls to the variability in the anatomy of root canals for maxillary premolars [26]. Additionally, it was reported that wide distal canals had significantly more untouched areas than narrow mesial canals in mandibular first molars. Thus preoperative canal anatomy had a major influence on the assessment of untouched areas. Micro-CT scans are used in endodontics to assess the preparation of root canals using stainless-steel hand endodontic instruments and motor-driven NiTi files [23, 25, 26]. They are used to compare endodontic features such as dentin volume removal, canal transportation, and centralization of files after canal preparation [23, 25, 26]. Micro-CT is an ideal method for in vitro studies due to its reliability, reproducibility, and non-destructiveness [27].

Finally, the present study has certain limitations and the results have to be interpreted carefully for clinical application. Since this study analyzed curved canals, which may not apply to all types of canals. Moreover, it examined the effect of a specific size of instruments that were selected for the study, making the results applicable only when the same set of instruments is used in vivo. Interestingly, EdgeEndo files and files used in rotary motion were among the most commonly used in the United States of America [28]. Therefore, clinicians might find this study helpful when they intend to treat curved canals using 04 thermally-treated rotary files. However, the findings need to be verified in vivo-setup.

## Conclusion

Within the limitation of the study, the coronal flaring with the ETP orifice opener and EGP file had no significant effect on the dentin volume removal and the percentage of untouched walls when used in conjunction with 04 Tapered RE and ES in a curved root canal system. Thus, the study proposes the effectiveness of using a single-size heat-treated file with a triangular cross-section to clean the curved MB canal. It further implies that coronal flaring and glide path files can be conservative while unnecessary to improve the shaping and cleaning in this situation. However, other potential benefits of using orifice openers and glide path files in terms of efficiency and safety in the hands of experienced and inexperienced clinicians can be explored in other studies.

## Supporting information

S1 File Raw data. (ZIP)

## References

[1] Siqueira Junior JF, Rôças I das N, Marceliano-Alves MF, Pérez AR, Ricucci D. Unprepared root canal surface areas: causes, clinical implications, and therapeutic strategies. Braz Oral Res. 2018;32(suppl 1):1–19. doi: 10.1590/1807-3107bor-2018.vol32.0065 30365606

[2] Alghamdi NS, Algarni YA, Ain TS, Alfaifi HM, AlQarni AA, Mashyakhi JQ, et al. Endodontic mishaps during root canal treatment performed by undergraduate dental students: An observational study. Medicine. 2021 Nov 24;100(47). doi: 10.1097/MD.0000000000027757 34964733 PMC8615340

[3] Eleftheriadis GI, Lambrianidis TP. Technical quality of root canal treatment and detection of iatrogenic errors in an undergraduate dental clinic. Int Endod J. 2005 Oct 1;38(10):725–34. doi: 10.1111/j.1365-2591.2005.01008.x 16164687

[4] Iandolo A, Iandolo G, Malvano M, Pantaleo G, Simeone M. Modern technologies in Endodontics. G Ital Endod. 2016 Jun 1;30(1):2–9.

[5] Abou-Rass M, Frank AL, Glick DH. The anticurvature filing method to prepare the curved root canal. J Am Dent Assoc. 1980 Nov 1;101(5):792–4. doi: 10.14219/jada.archive.1980.0427 6935269

[6] Morgan LF, Montgomery S. An evaluation of the crown-down pressureless technique. J Endod. 1984 Oct 1;10(10):491–8. doi: 10.1016/S0099-2399(84)80207-1 6593413

[7] Riitano F, Riitano F. Anatomic Endodontic Technology (AET)–a crown-down root canal preparation technique: basic concepts, operative procedure and instruments. Int Endod J. 2005 Aug 1;38(8):575–87. doi: 10.1111/j.1365-2591.2005.00970.x 16011778

[8] Chaniotis A, Ordinola-Zapata R. Present status and future directions: Management of curved and calcified root canals. Int Endod J. 2022 May 1;55(S3):656–84. doi: 10.1111/iej.13685 35106792

[9] Hülsmann M, Peters OA, Dummer PMH. Mechanical preparation of root canals: shaping goals, techniques and means. Endod Topics. 2005 Mar 1;10(1):30–76.

[10] Ballester B, Giraud T, Ahmed HMA, Nabhan MS, Bukiet F, Guivarc’h M. Current strategies for conservative endodontic access cavity preparation techniques—systematic review, meta-analysis, and decision-making protocol. Clin Oral Investig. 2021 Nov 1;25(11):6027–44. doi: 10.1007/s00784-021-04080-7 34623506

[11] Zupanc J, Vahdat-Pajouh N, Schäfer E. New thermomechanically treated NiTi alloys—a review. Int Endod J. 2018 Oct;51(10):1088–103. doi: 10.1111/iej.12924 29574784

[12] Donnermeyer D, Viedenz A, Schäfer E, Bürklein S. Impact of new cross-sectional designs on the shaping ability of rotary NiTi instruments in S-shaped canals. Odontology [Internet]. 2020 Apr 1 [cited 2024 Feb 9];108(2):174–9. Available from: https://link.springer.com/article/10.1007/s10266-019-00450-6 31446499 10.1007/s10266-019-00450-6

[13] Schäfer E, Oitzinger M. Cutting Efficiency of Five Different Types of Rotary Nickel–Titanium Instruments. J Endod. 2008 Feb 1; 34(2):198–200. doi: 10.1016/j.joen.2007.10.009 18215681

[14] Plotino G, Nagendrababu V, Bukiet F, Grande NM, Veettil SK, De-Deus G, et al. Influence of Negotiation, Glide Path, and Preflaring Procedures on Root Canal Shaping—Terminology, Basic Concepts, and a Systematic Review. J Endod. 2020 Jun 1;46(6):707–29. doi: 10.1016/j.joen.2020.01.023 32334856

[15] Ajina M, Billis G, Chong BS. The Effect of Glide Path Preparation on Root Canal Shaping Procedures and Outcomes. Eur Endod J. 2022 Jun 1;7(2):92–105. doi: 10.14744/eej.2022.97659 35786583 PMC9285994

[16] Vertucci FJ. Root canal anatomy of the human permanent teeth. Oral Surgery, Oral Medicine, Oral Pathology. 1984 Nov 1;58(5):589–99. doi: 10.1016/0030-4220(84)90085-9 6595621

[17] Schneider SW. A comparison of canal preparations in straight and curved root canals. Oral Surg Oral Med Oral Pathol. 1971;32(2):271–5. doi: 10.1016/0030-4220(71)90230-1 5284110

[18] Assiry AA, Karobari MI, Lin GSS, Batul R, Snigdha NT, Luke AM, et al. Microstructural and Elemental Characterization of Root Canal Sealers Using FTIR, SEM, and EDS Analysis. Applied Sciences 2023, Vol 13, Page 4517 [Internet]. 2023 Apr 2 [cited 2024 Feb 8];13(7):4517. Available from: https://www.mdpi.com/2076-3417/13/7/4517/htm

[19] Chan MYC, Cheung V, Lee AHC, Zhang C. A Literature Review of Minimally Invasive Endodontic Access Cavities—Past, Present and Future. Eur Endod J. 2022 Mar 1;7(1):1. doi: 10.14744/eej.2022.62681 35353062 PMC9035856

[20] Marvaniya J, Agarwal K, Mehta DN, Parmar N, Shyamal R, Patel J. Minimal Invasive Endodontics: A Comprehensive Narrative Review. Cureus. 2022 Jun 16;14(6). doi: 10.7759/cureus.25984 35859953 PMC9287844

[21] Duarte MAH, Bernardes RA, Ordinola-Zapata R, de Vasconcelos BC, Bramante CM, de Moraes IG. Effects of Gates-Glidden, LA Axxess and orifice shaper burs on the cervical dentin thickness and root canal area of mandibular molars. Braz Dent J. 2011;22(1):28–31. doi: 10.1590/s0103-64402011000100004 21519644

[22] Heyse JD, Ordinola-Zapata R, Gaalaas L, McClanahan SB. The effect of rotary instrumentation on dentin thickness in the danger zone of the MB2 canal of maxillary first molars. Australian Endodontic Journal. 2022 Aug 1;48(2):239–44. doi: 10.1111/aej.12555 34351045

[23] Elnaghy AM, Elsaka SE. Evaluation of root canal transportation, centering ratio, and remaining dentin thickness associated with ProTaper Next instruments with and without glide path. J Endod. 2014;40(12):2053–6. doi: 10.1016/j.joen.2014.09.001 25301350

[24] Vorster M, van der Vyver PJ, Paleker F. Canal Transportation and Centering Ability of WaveOne Gold in Combination with and without Different Glide Path Techniques. J Endod. 2018 Sep 1;44(9):1430–5. doi: 10.1016/j.joen.2018.06.003 30078574

[25] Zhang Y, Liu J, Gu Y, Wang J, Xu H, Zhang G. Analysis of second mesiobuccal root canal instrumentation in maxillary first molars with three nickel-titanium rotary instruments: a micro-computed tomographic study. Odontology. 2021 Apr 1;109(2):496–505. doi: 10.1007/s10266-020-00564-2 33175279

[26] Perez Morales M de las N, González Sánchez JA, Olivieri Fernández JG, Laperre K, Abella Sans F, Jaramillo DE, et al. TRUShape Versus XP-endo Shaper: A Micro-computed Tomographic Assessment and Comparative Study of the Shaping Ability-An In Vitro Study. J Endod. 2020 Feb 1; 46(2):271–6. 31839412 10.1016/j.joen.2019.10.027

[27] Versiani MA, Keleș A. Applications of Micro-CT Technology in Endodontics. Micro-computed Tomography (micro-CT) in Medicine and Engineering. 2020;183–211.

[28] Logsdon J, Dunlap C, Arias A, Scott R, Peters OA. Current Trends in Use and Reuse of Nickel-Titanium Engine-driven Instruments: A Survey of Endodontists in the United States. J Endod. 2020 Mar 1;46(3):391–6. doi: 10.1016/j.joen.2019.12.011 32029265

