# Correction: Microcomputed tomography analysis of curved root canal preparation when coronal flaring and glide path files used with heat-treated nickel titanium rotary files

**DOI:** 10.1371/journal.pone.0306193

**Published:** 2024-06-21

**Authors:** Ammar AbuMostafa, Mohammed M. Alrefaie, Nedal Abu-Mostafa, Fahda N. Algahtani

In the Funding section, the Researchers Supporting Project Number from the funder Princess Nourah bint Abdulrahman University is listed incorrectly. The correct project number is: PNURSP2024R363.
