# Supplementary figures and images for: Microcomputed tomography analysis of curved root canal preparation when coronal flaring and glide path files used with heat-treated nickel titanium rotary files (part 2 of 2)
Source: PLoS One. 2024 Apr 3;19(4):e0299896. doi: 10.1371/journal.pone.0299896 (PMC10990200; doi:10.1371/journal.pone.0299896)

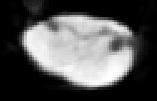

Supplement: S1 File — (ZIP) [file pone.0299896.s001.zip › Dra. Ola/Results & Images/22/1mm post.JPG]

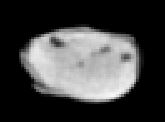

Supplement: S1 File — (ZIP) [file pone.0299896.s001.zip › Dra. Ola/Results & Images/22/1mm pre.JPG]

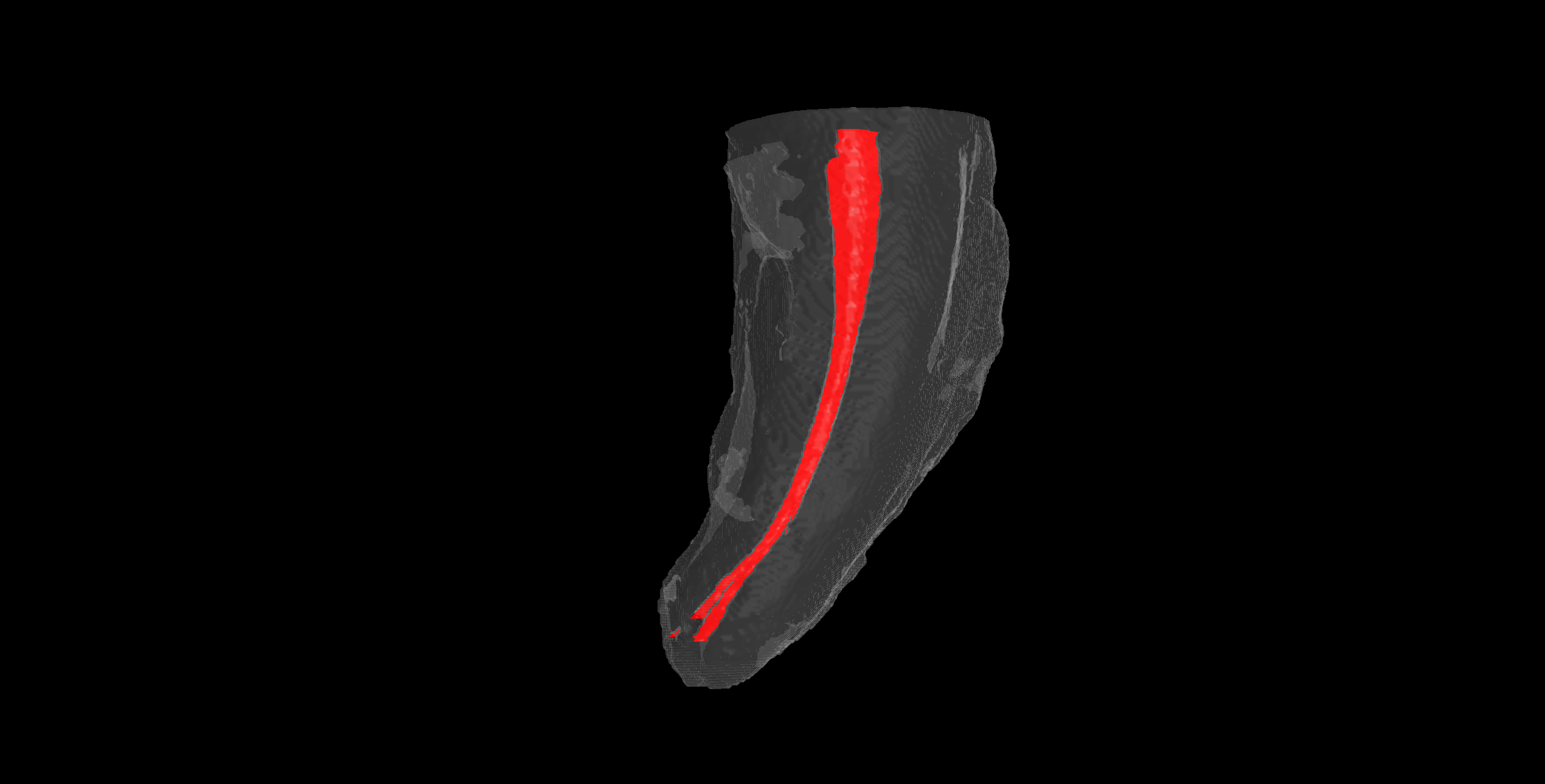

Supplement: S1 File — (ZIP) [file pone.0299896.s001.zip › Dra. Ola/Results & Images/22/22_buc.bmp]

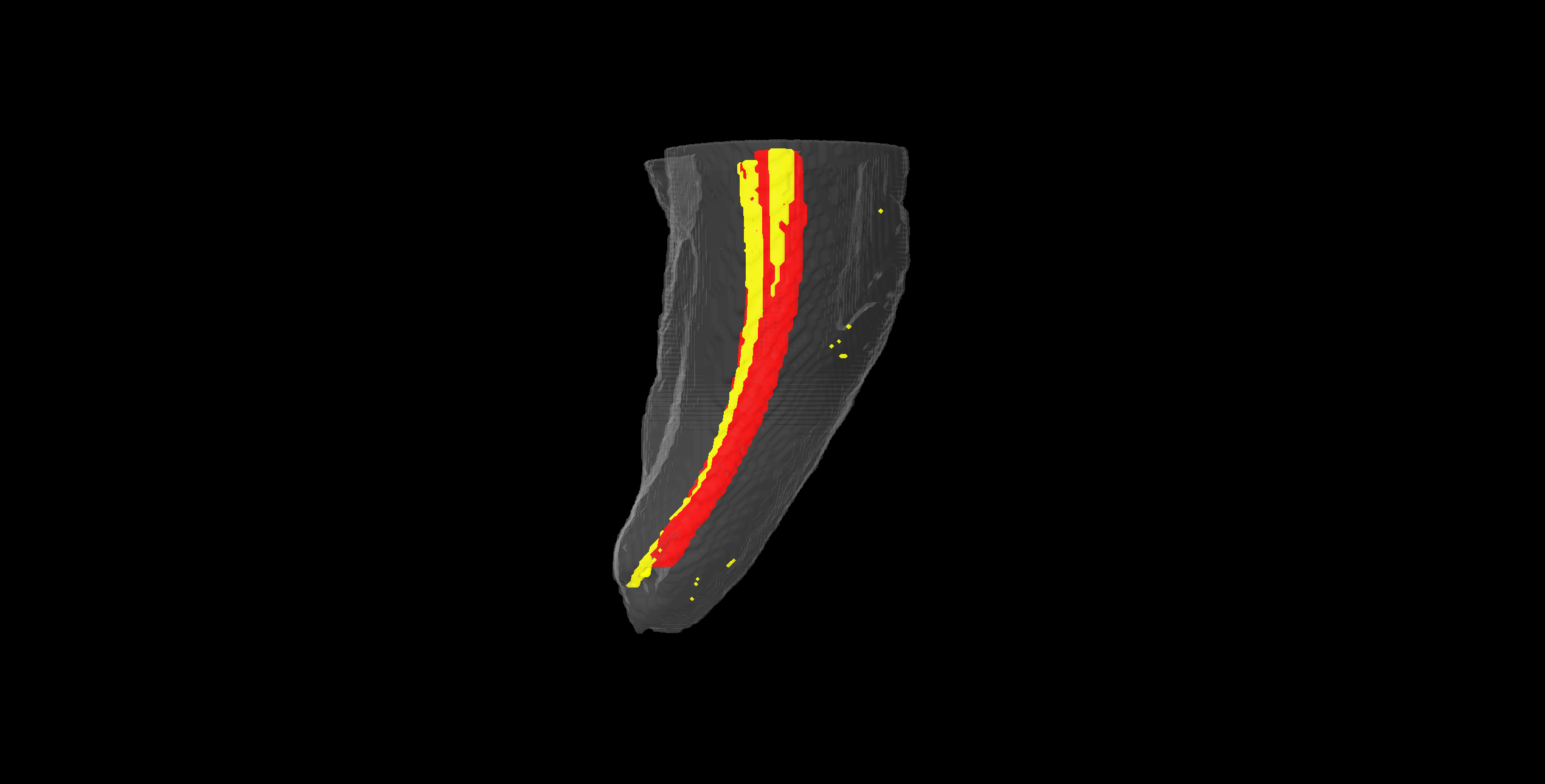

Supplement: S1 File — (ZIP) [file pone.0299896.s001.zip › Dra. Ola/Results & Images/22/22_buc_2.bmp]

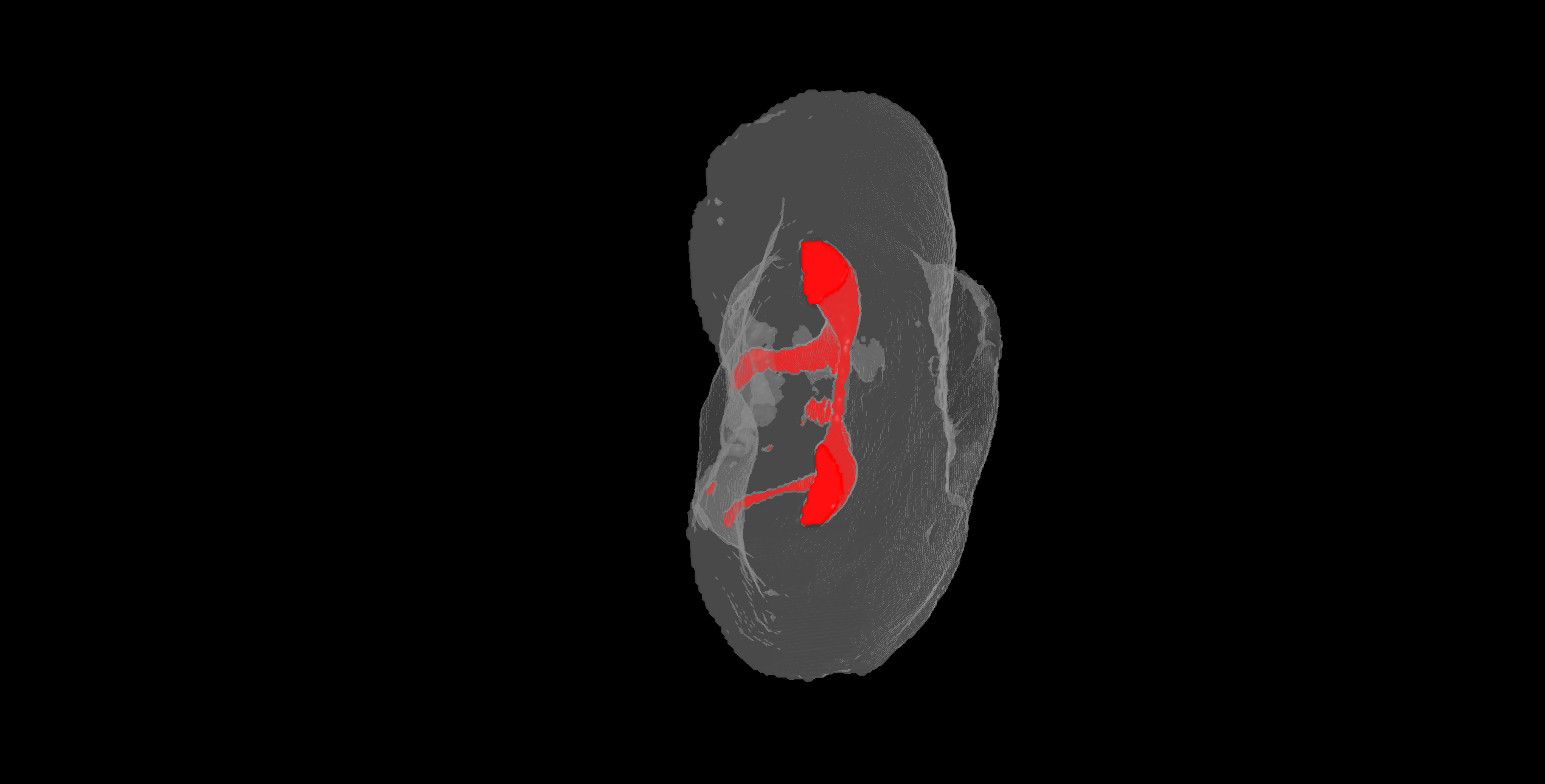

Supplement: S1 File — (ZIP) [file pone.0299896.s001.zip › Dra. Ola/Results & Images/22/22_cor.bmp]

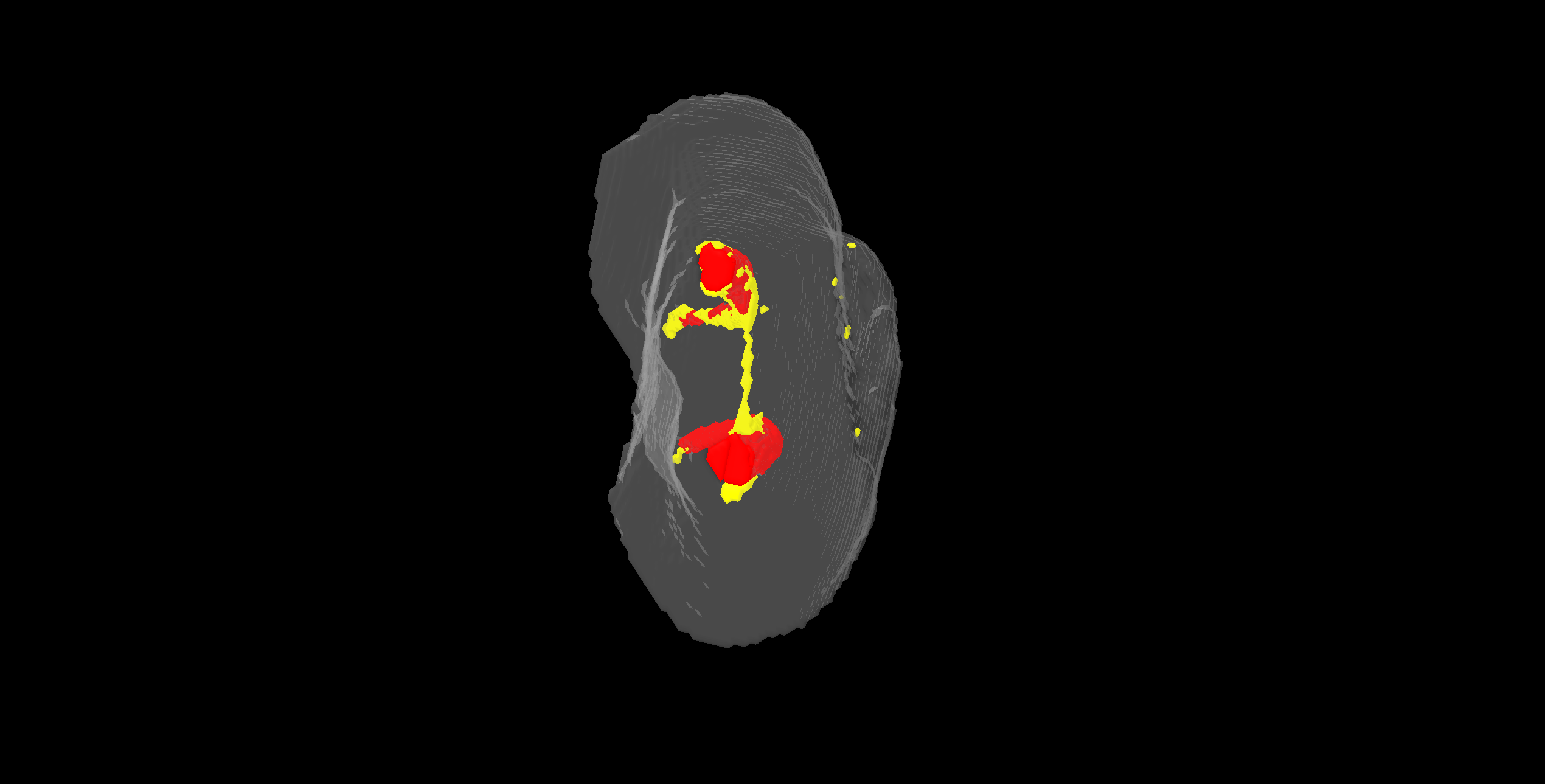

Supplement: S1 File — (ZIP) [file pone.0299896.s001.zip › Dra. Ola/Results & Images/22/22_cor_2.bmp]

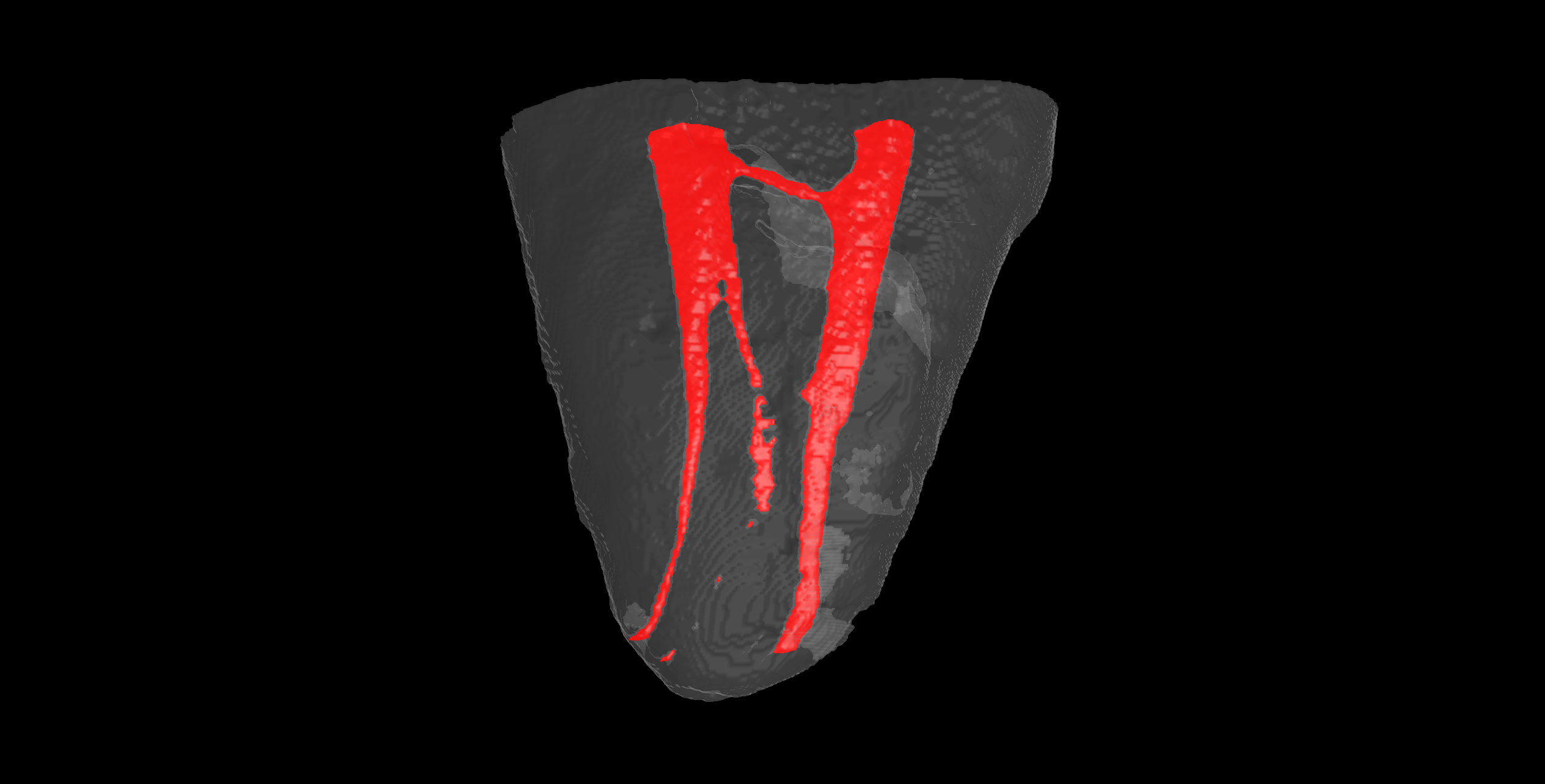

Supplement: S1 File — (ZIP) [file pone.0299896.s001.zip › Dra. Ola/Results & Images/22/22_mes.bmp]

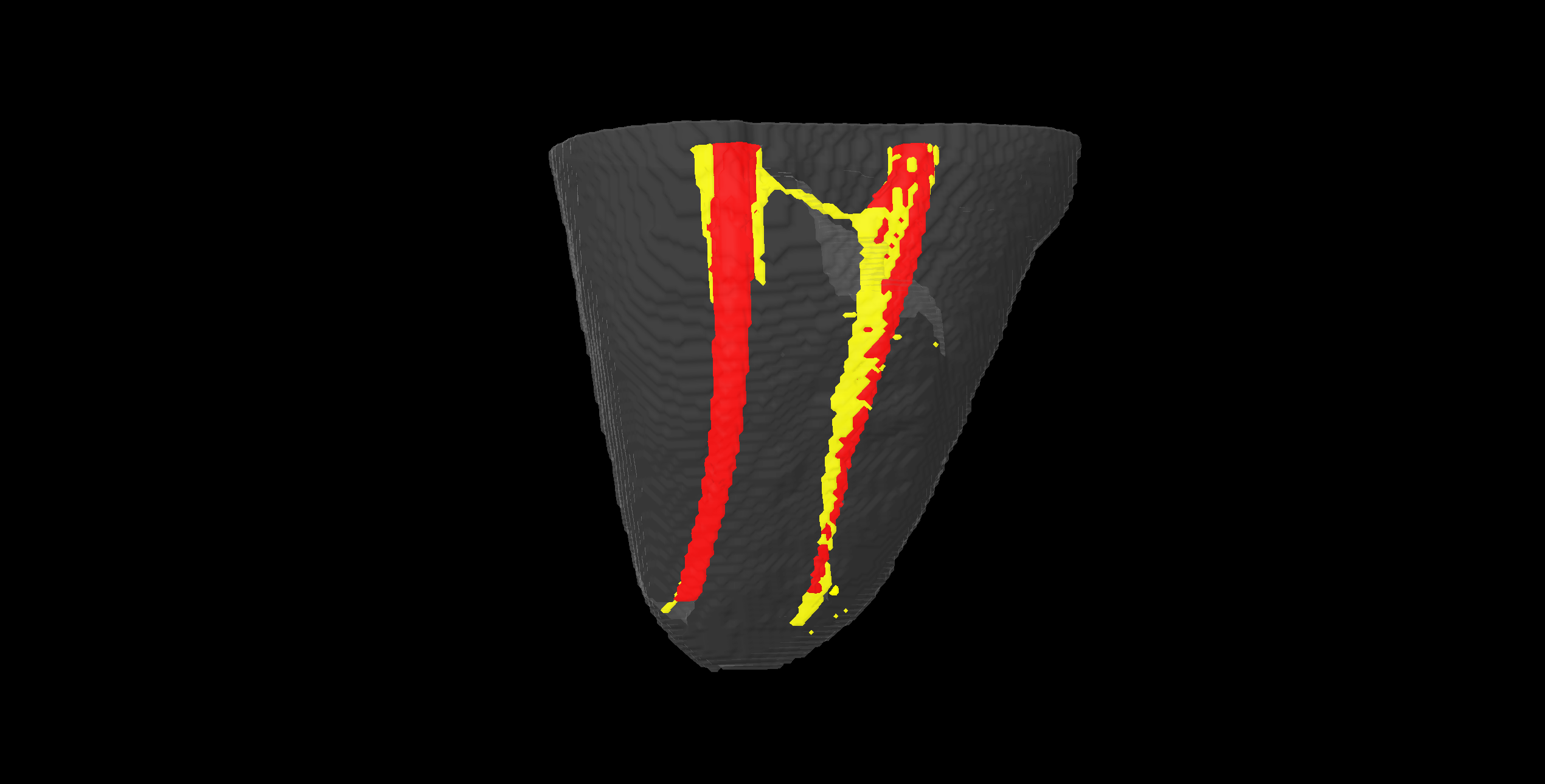

Supplement: S1 File — (ZIP) [file pone.0299896.s001.zip › Dra. Ola/Results & Images/22/22_mes_2.bmp]

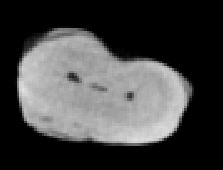

Supplement: S1 File — (ZIP) [file pone.0299896.s001.zip › Dra. Ola/Results & Images/22/3mm pre.JPG]

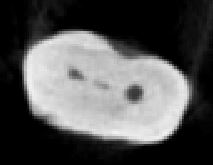

Supplement: S1 File — (ZIP) [file pone.0299896.s001.zip › Dra. Ola/Results & Images/22/3mmm post.JPG]

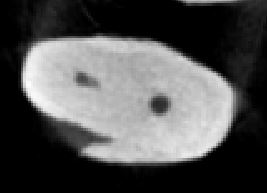

Supplement: S1 File — (ZIP) [file pone.0299896.s001.zip › Dra. Ola/Results & Images/22/5mm post.JPG]

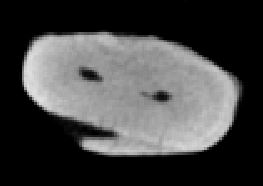

Supplement: S1 File — (ZIP) [file pone.0299896.s001.zip › Dra. Ola/Results & Images/22/5mm pre.JPG]

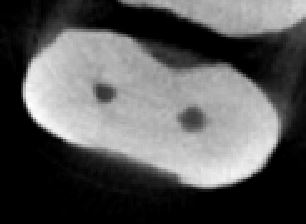

Supplement: S1 File — (ZIP) [file pone.0299896.s001.zip › Dra. Ola/Results & Images/22/7mm post.JPG]

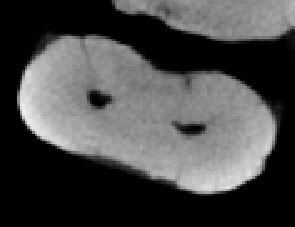

Supplement: S1 File — (ZIP) [file pone.0299896.s001.zip › Dra. Ola/Results & Images/22/7mm pre.JPG]

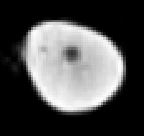

Supplement: S1 File — (ZIP) [file pone.0299896.s001.zip › Dra. Ola/Results & Images/25/1mm post.JPG]

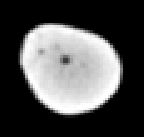

Supplement: S1 File — (ZIP) [file pone.0299896.s001.zip › Dra. Ola/Results & Images/25/1mm pre.JPG]

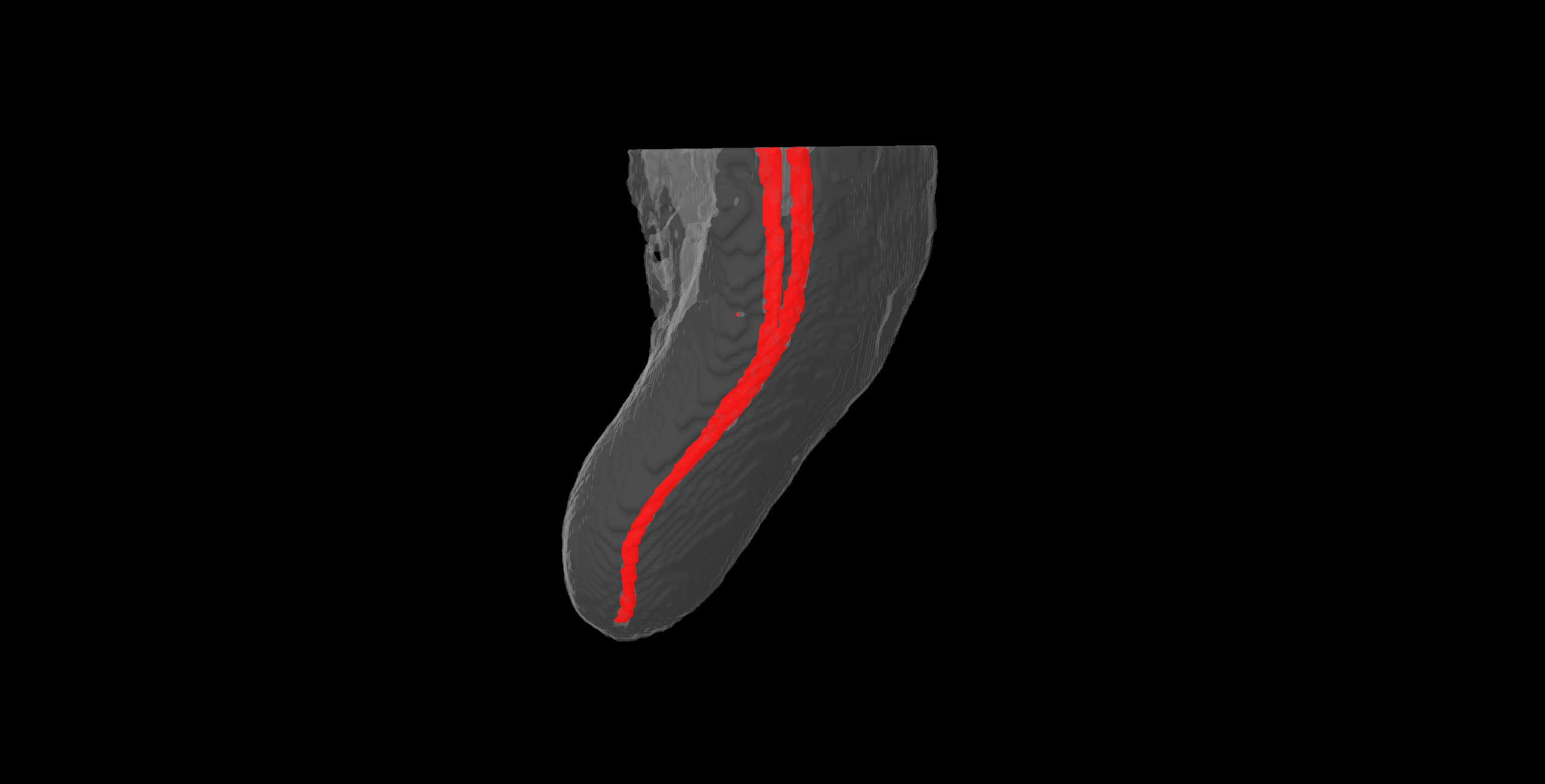

Supplement: S1 File — (ZIP) [file pone.0299896.s001.zip › Dra. Ola/Results & Images/25/25_buc.bmp]

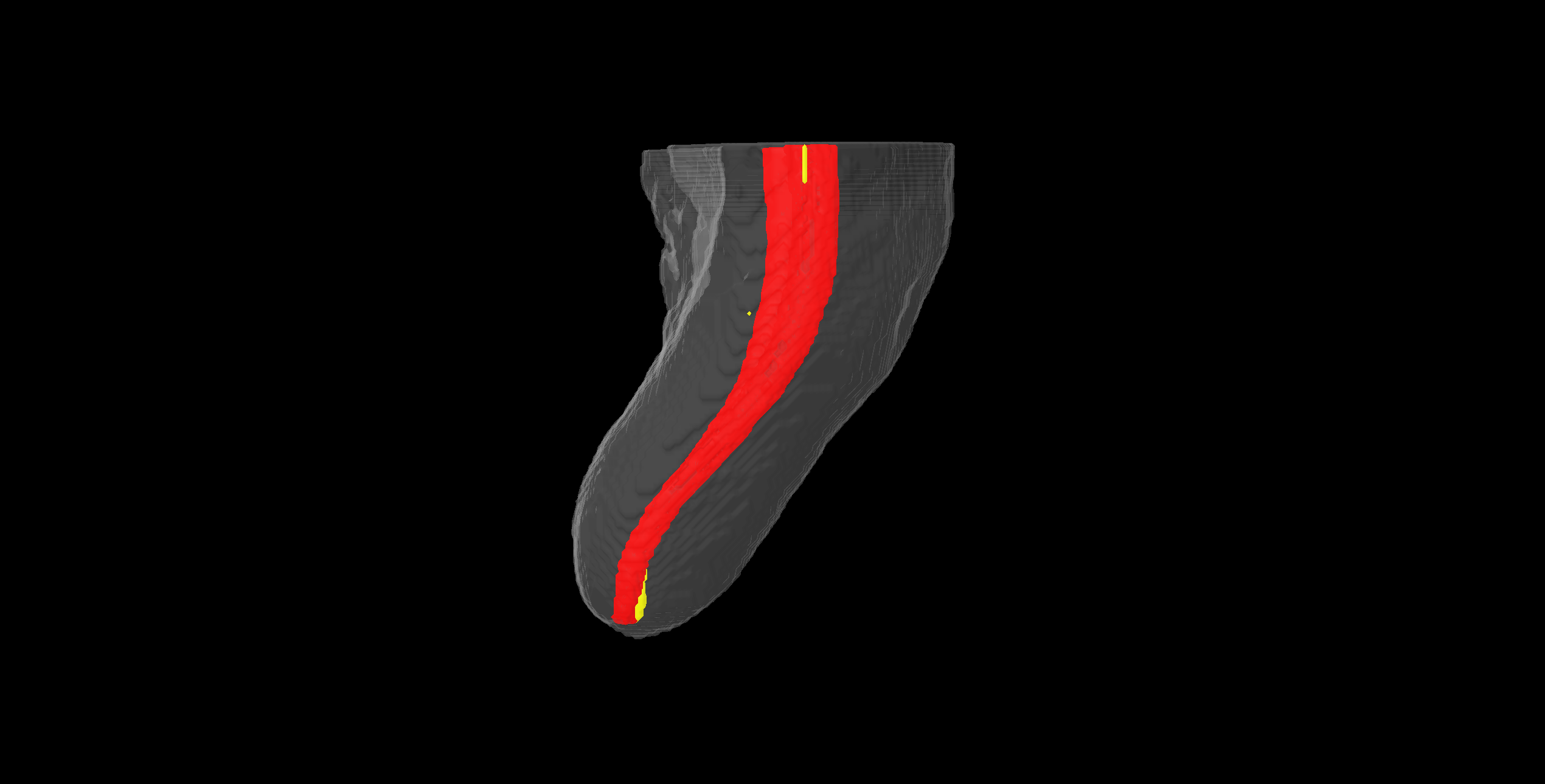

Supplement: S1 File — (ZIP) [file pone.0299896.s001.zip › Dra. Ola/Results & Images/25/25_buc_2.bmp]

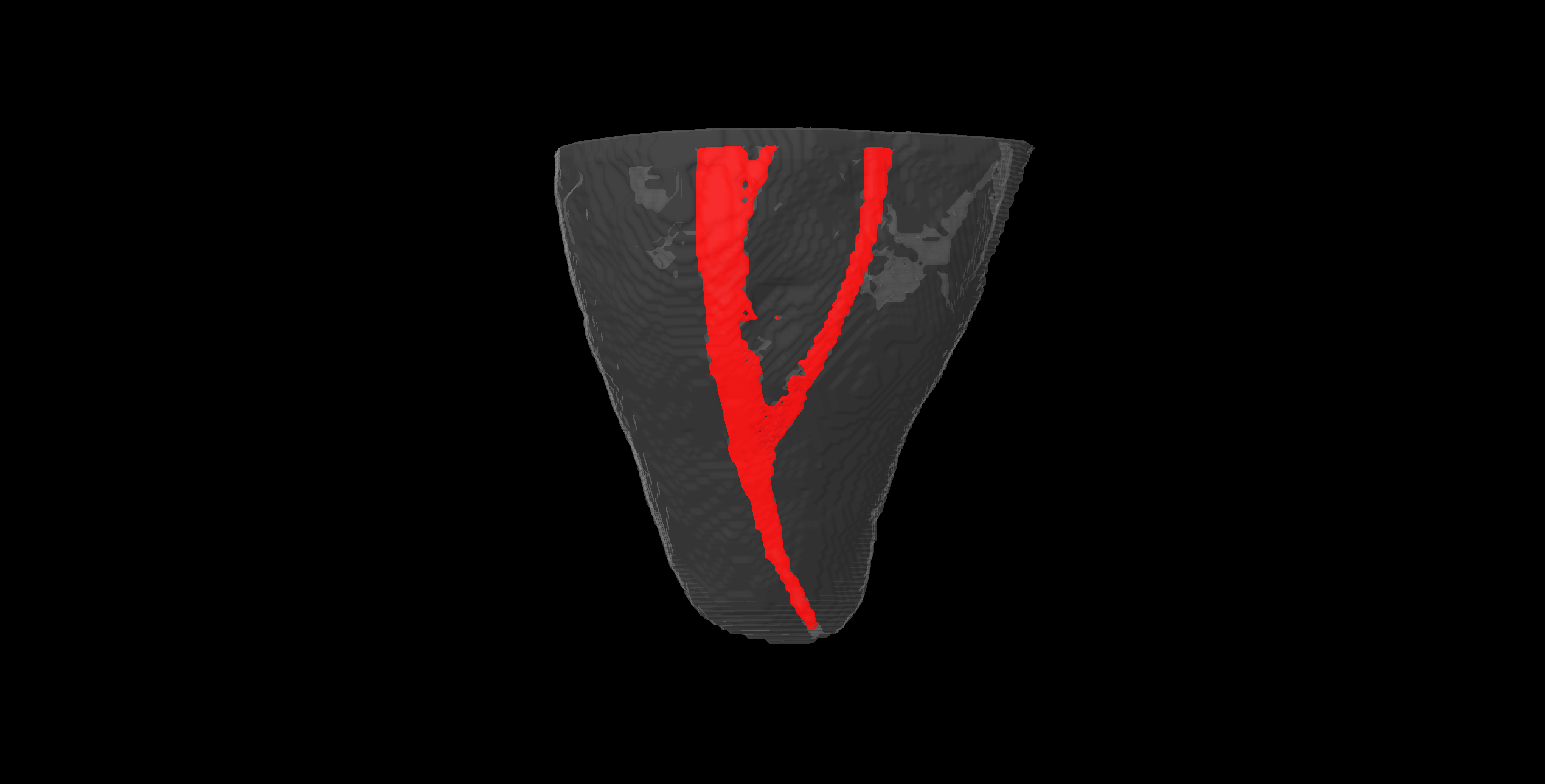

Supplement: S1 File — (ZIP) [file pone.0299896.s001.zip › Dra. Ola/Results & Images/25/25_mes.bmp]

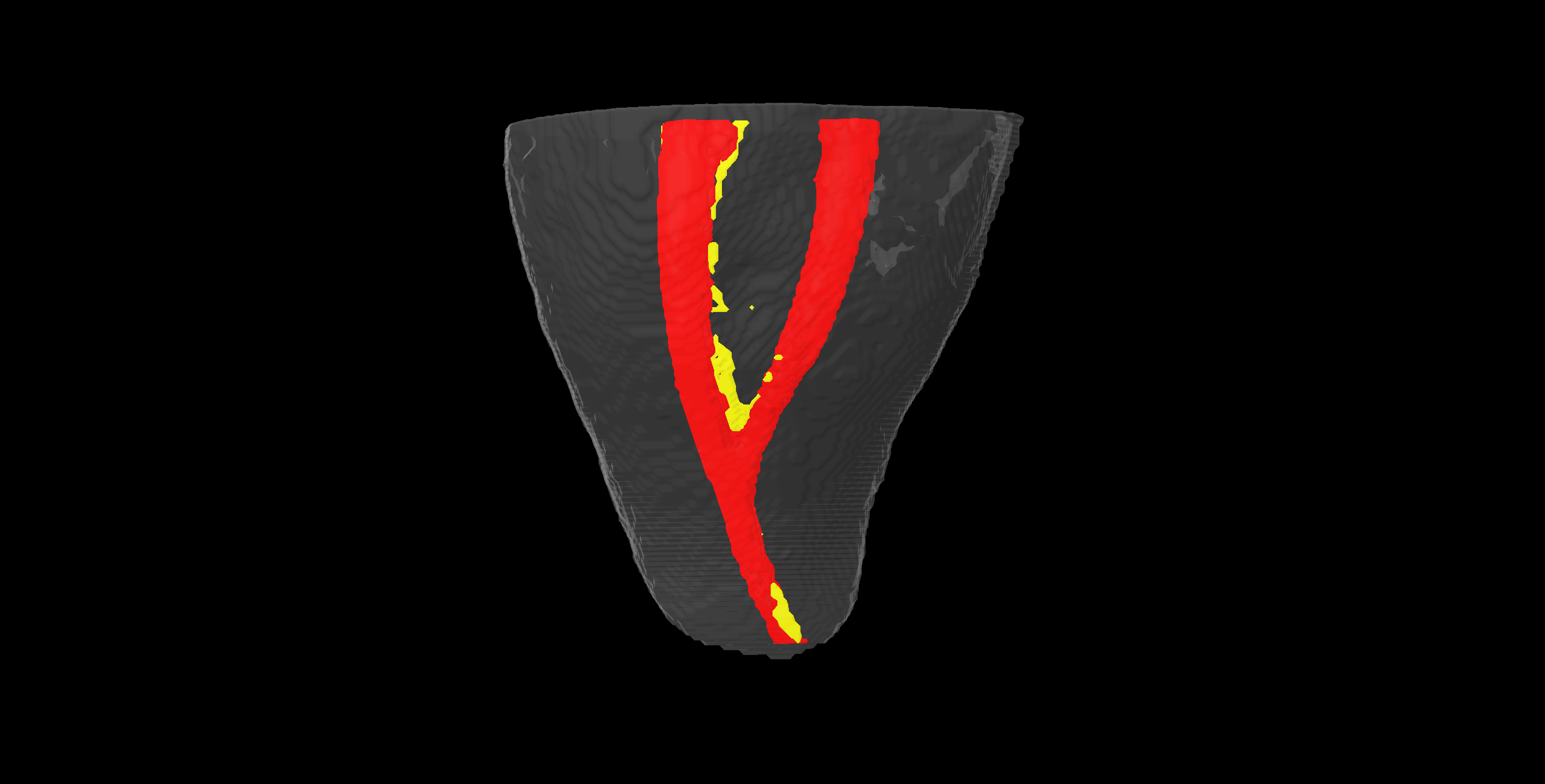

Supplement: S1 File — (ZIP) [file pone.0299896.s001.zip › Dra. Ola/Results & Images/25/25_mes_2.bmp]

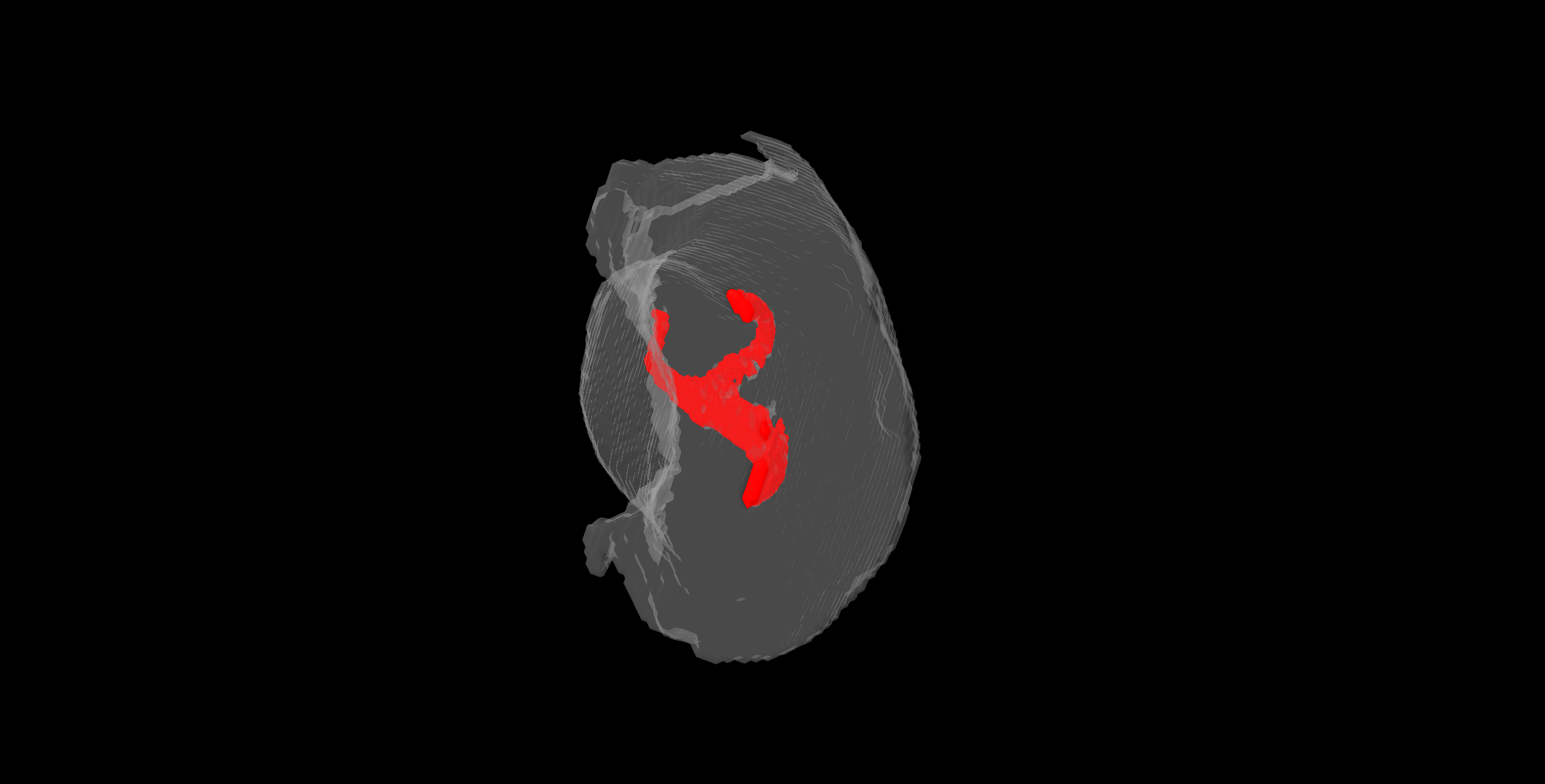

Supplement: S1 File — (ZIP) [file pone.0299896.s001.zip › Dra. Ola/Results & Images/25/25_occ.bmp]

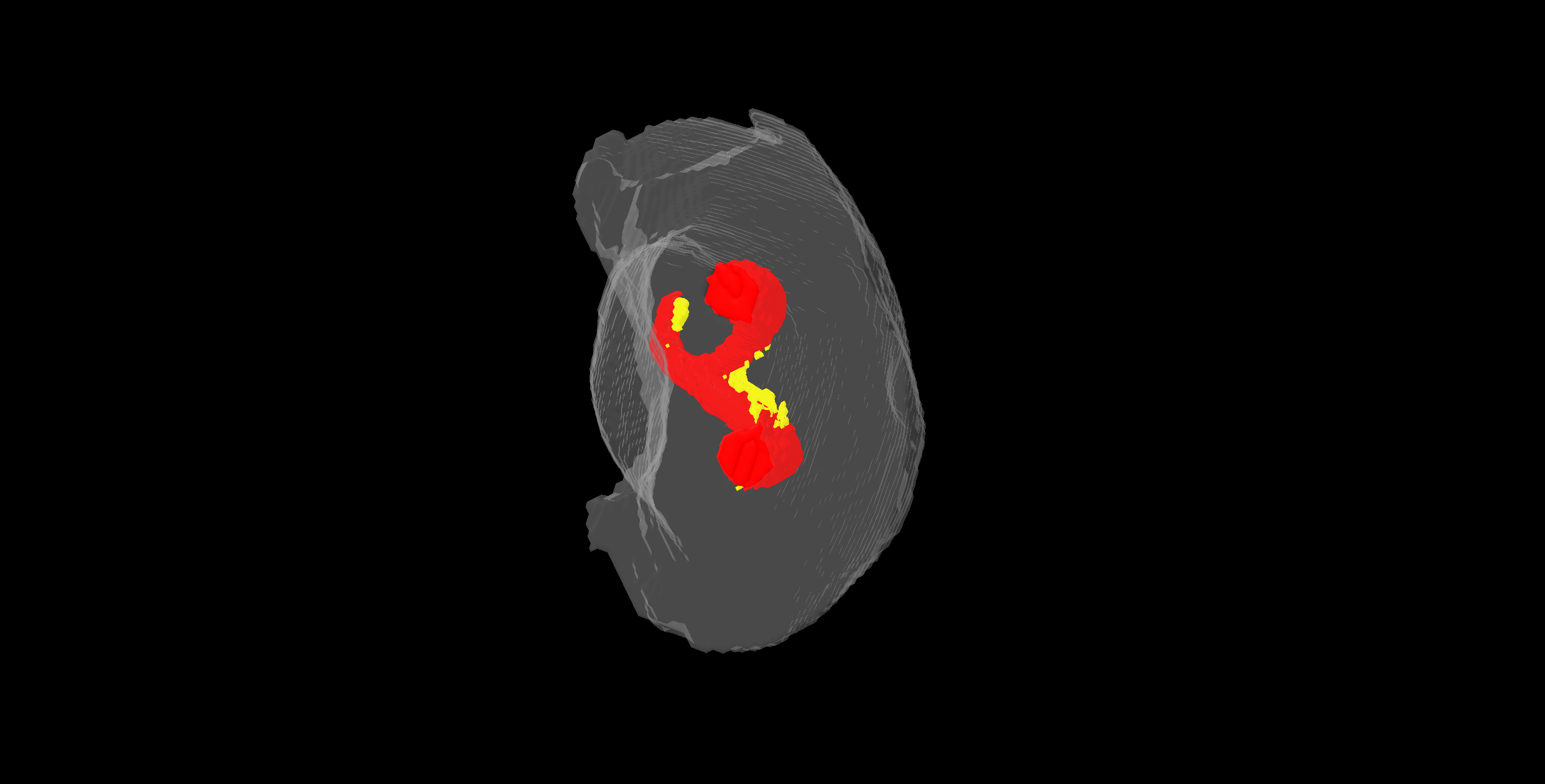

Supplement: S1 File — (ZIP) [file pone.0299896.s001.zip › Dra. Ola/Results & Images/25/25_occ_2.bmp]

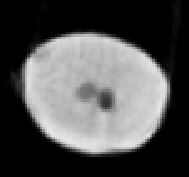

Supplement: S1 File — (ZIP) [file pone.0299896.s001.zip › Dra. Ola/Results & Images/25/3mm post.JPG]

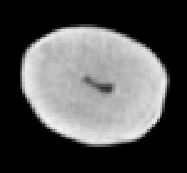

Supplement: S1 File — (ZIP) [file pone.0299896.s001.zip › Dra. Ola/Results & Images/25/3mm pre.JPG]

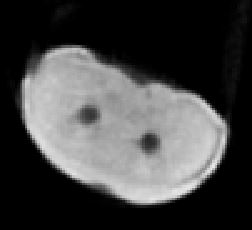

Supplement: S1 File — (ZIP) [file pone.0299896.s001.zip › Dra. Ola/Results & Images/25/5mm post.JPG]

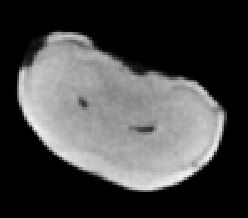

Supplement: S1 File — (ZIP) [file pone.0299896.s001.zip › Dra. Ola/Results & Images/25/5mm pre.JPG]

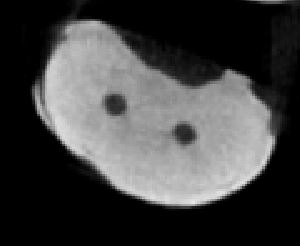

Supplement: S1 File — (ZIP) [file pone.0299896.s001.zip › Dra. Ola/Results & Images/25/7mm post.JPG]

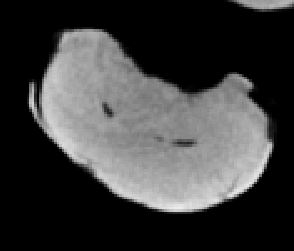

Supplement: S1 File — (ZIP) [file pone.0299896.s001.zip › Dra. Ola/Results & Images/25/7mm pre.JPG]

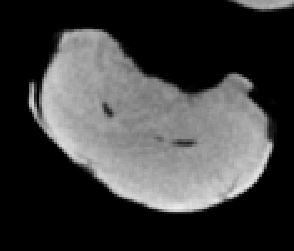

Supplement: S1 File — (ZIP) [file pone.0299896.s001.zip › Dra. Ola/Results & Images/25/7mm pre.JPG~RF86b684.TMP]

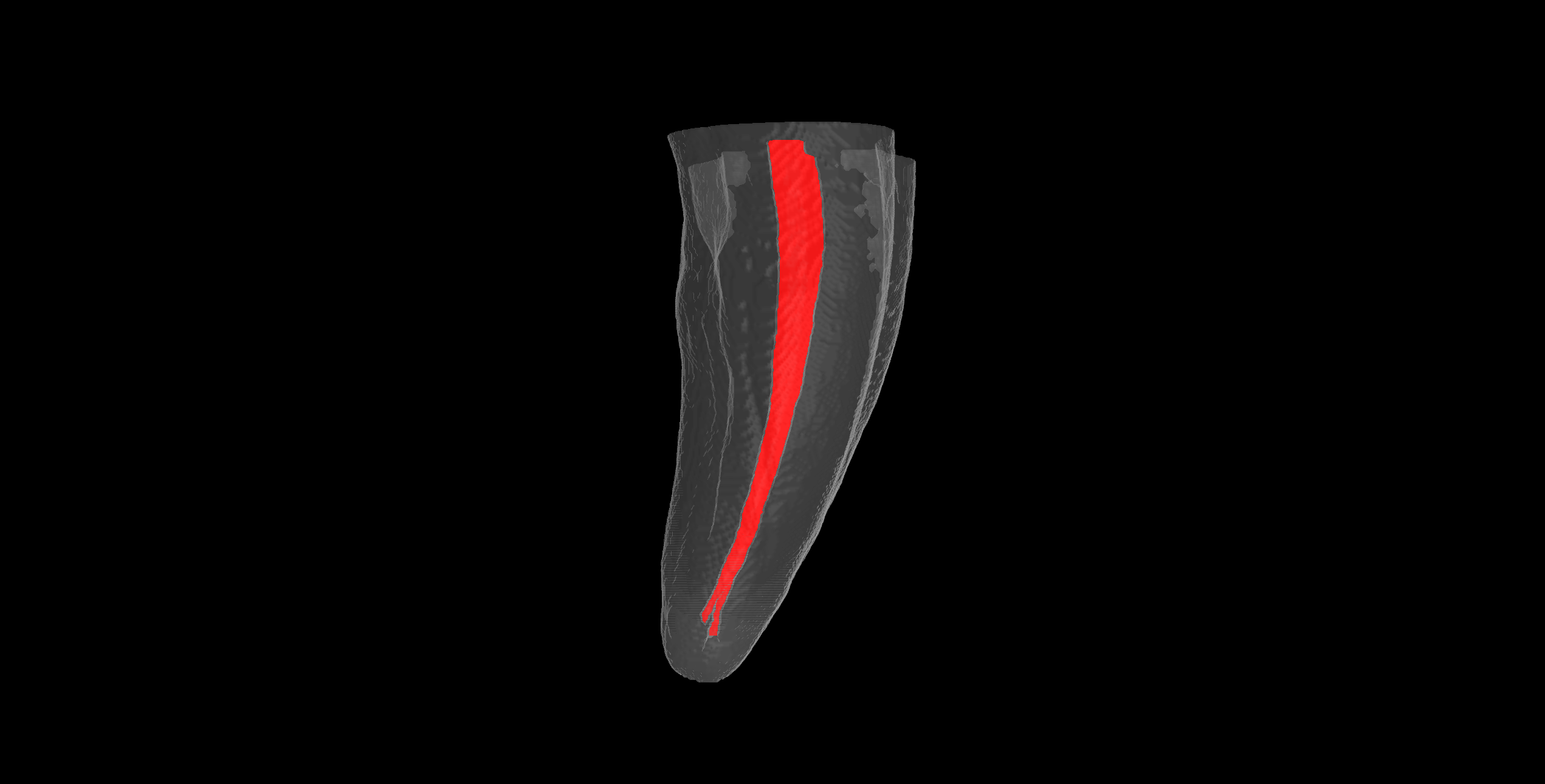

Supplement: S1 File — (ZIP) [file pone.0299896.s001.zip › Dra. Ola/Results & Images/26/26_buc.bmp]

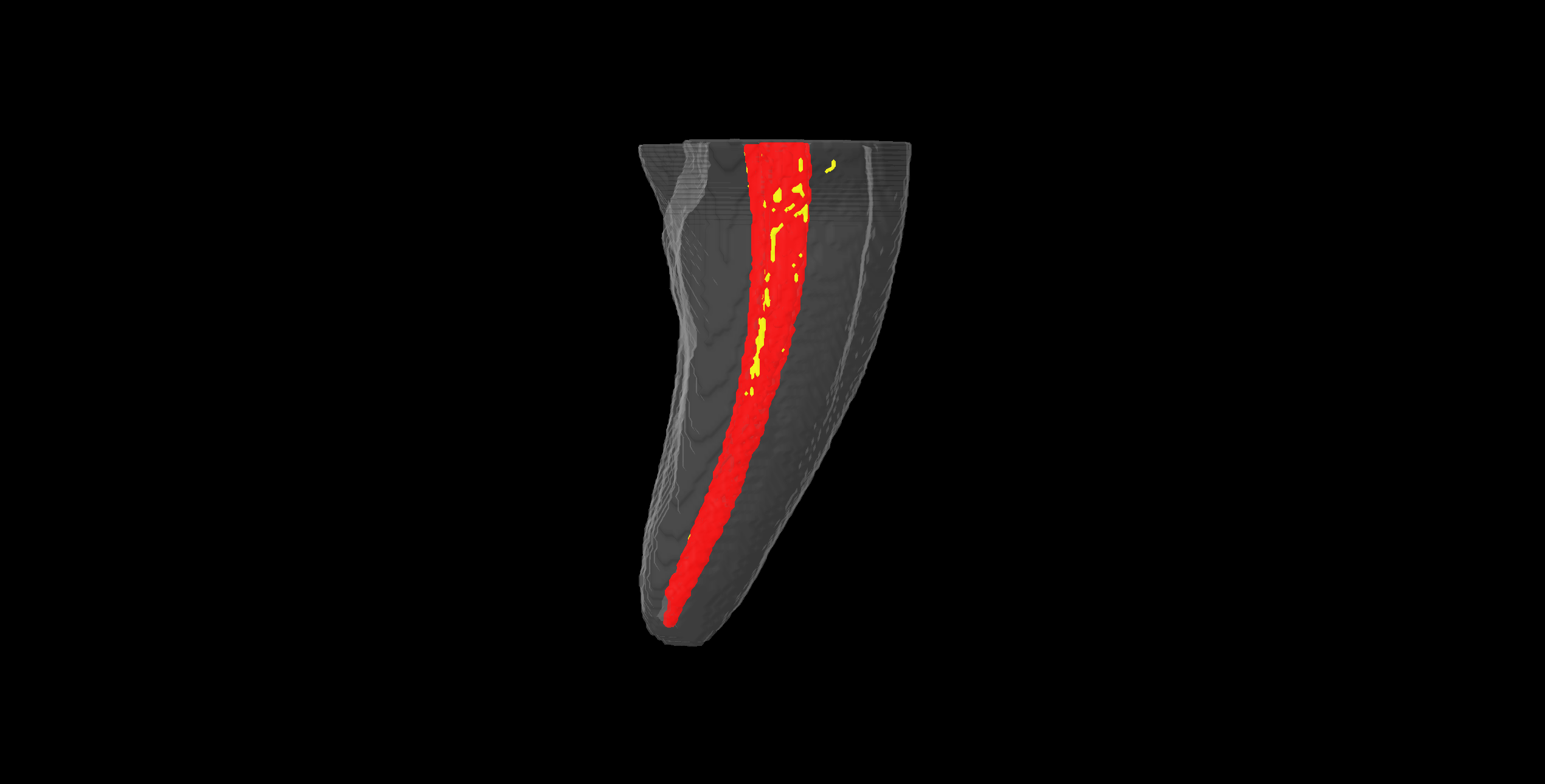

Supplement: S1 File — (ZIP) [file pone.0299896.s001.zip › Dra. Ola/Results & Images/26/26_buc_2.bmp]

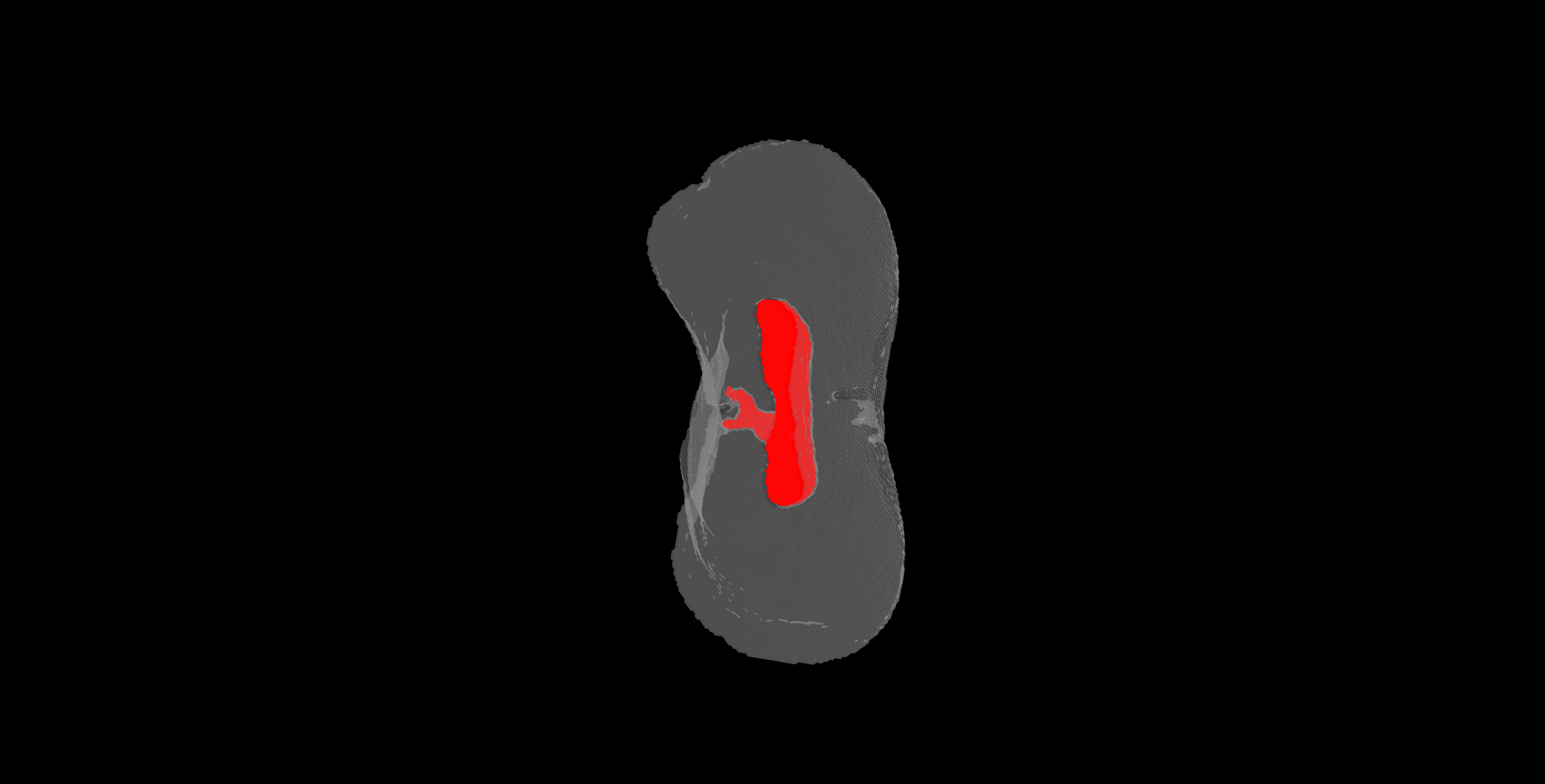

Supplement: S1 File — (ZIP) [file pone.0299896.s001.zip › Dra. Ola/Results & Images/26/26_cor.bmp]

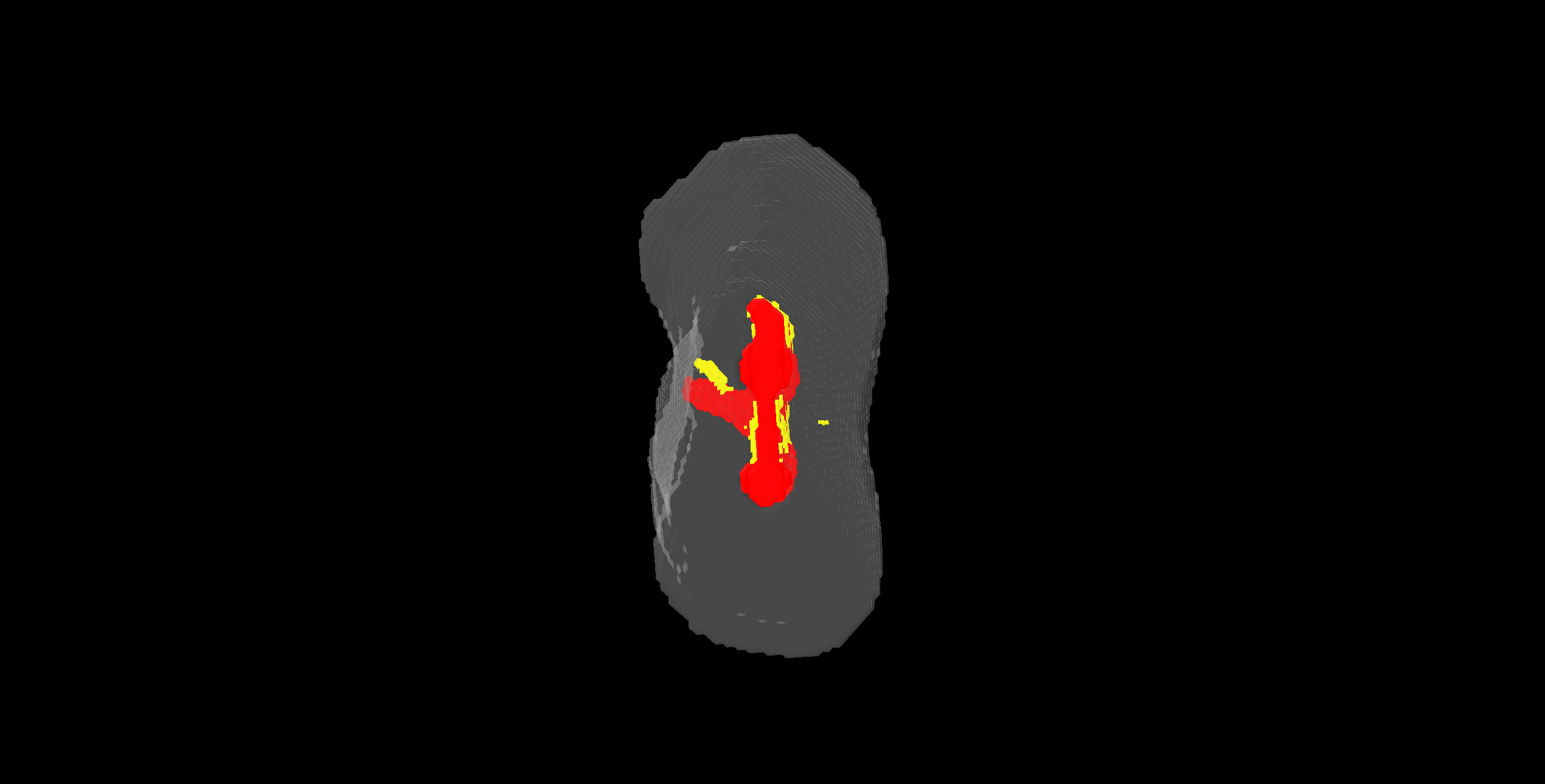

Supplement: S1 File — (ZIP) [file pone.0299896.s001.zip › Dra. Ola/Results & Images/26/26_cor_2.bmp]

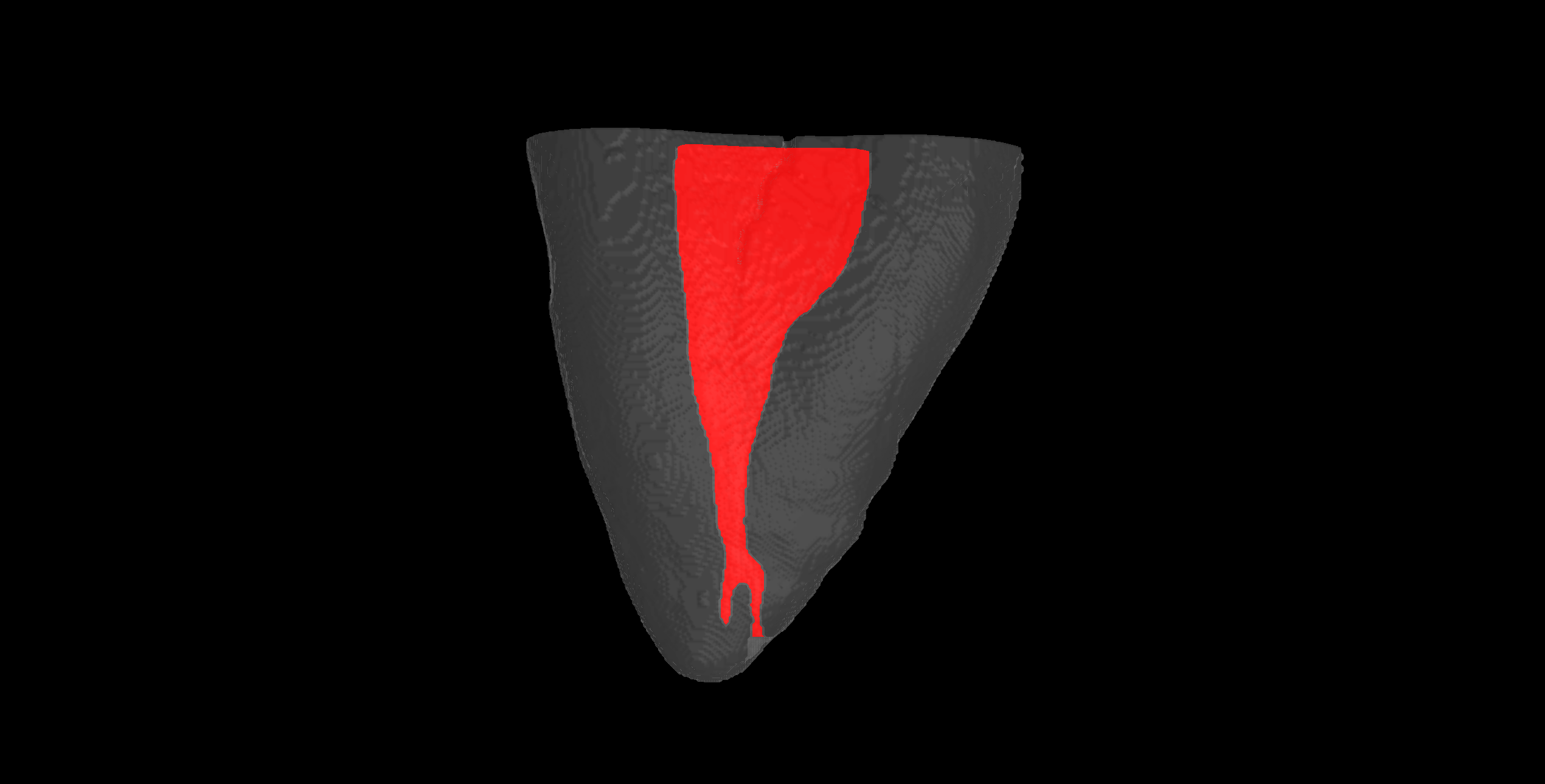

Supplement: S1 File — (ZIP) [file pone.0299896.s001.zip › Dra. Ola/Results & Images/26/26_mes.bmp]

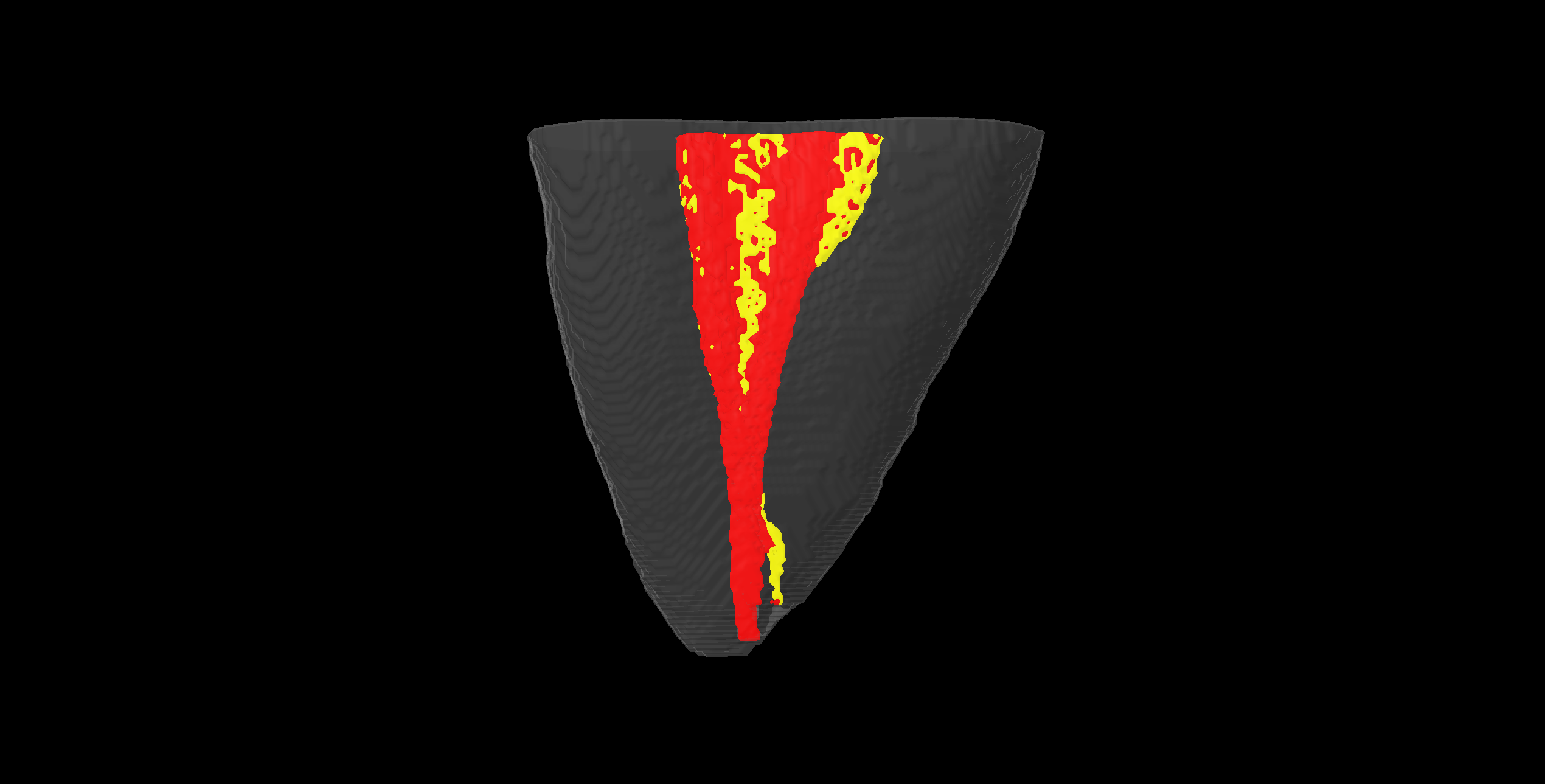

Supplement: S1 File — (ZIP) [file pone.0299896.s001.zip › Dra. Ola/Results & Images/26/26_mes_2.bmp]

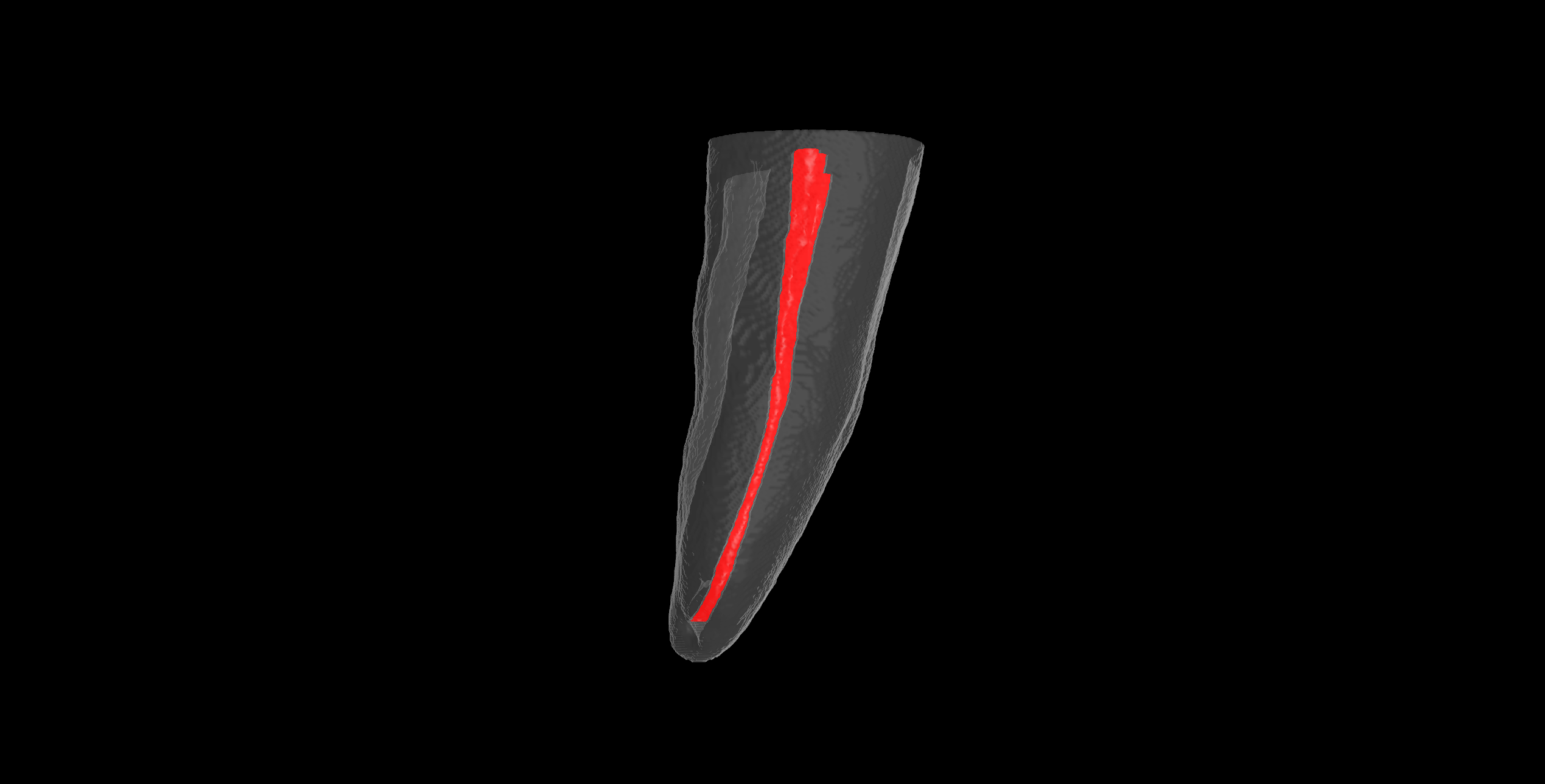

Supplement: S1 File — (ZIP) [file pone.0299896.s001.zip › Dra. Ola/Results & Images/28/28_buc.bmp]

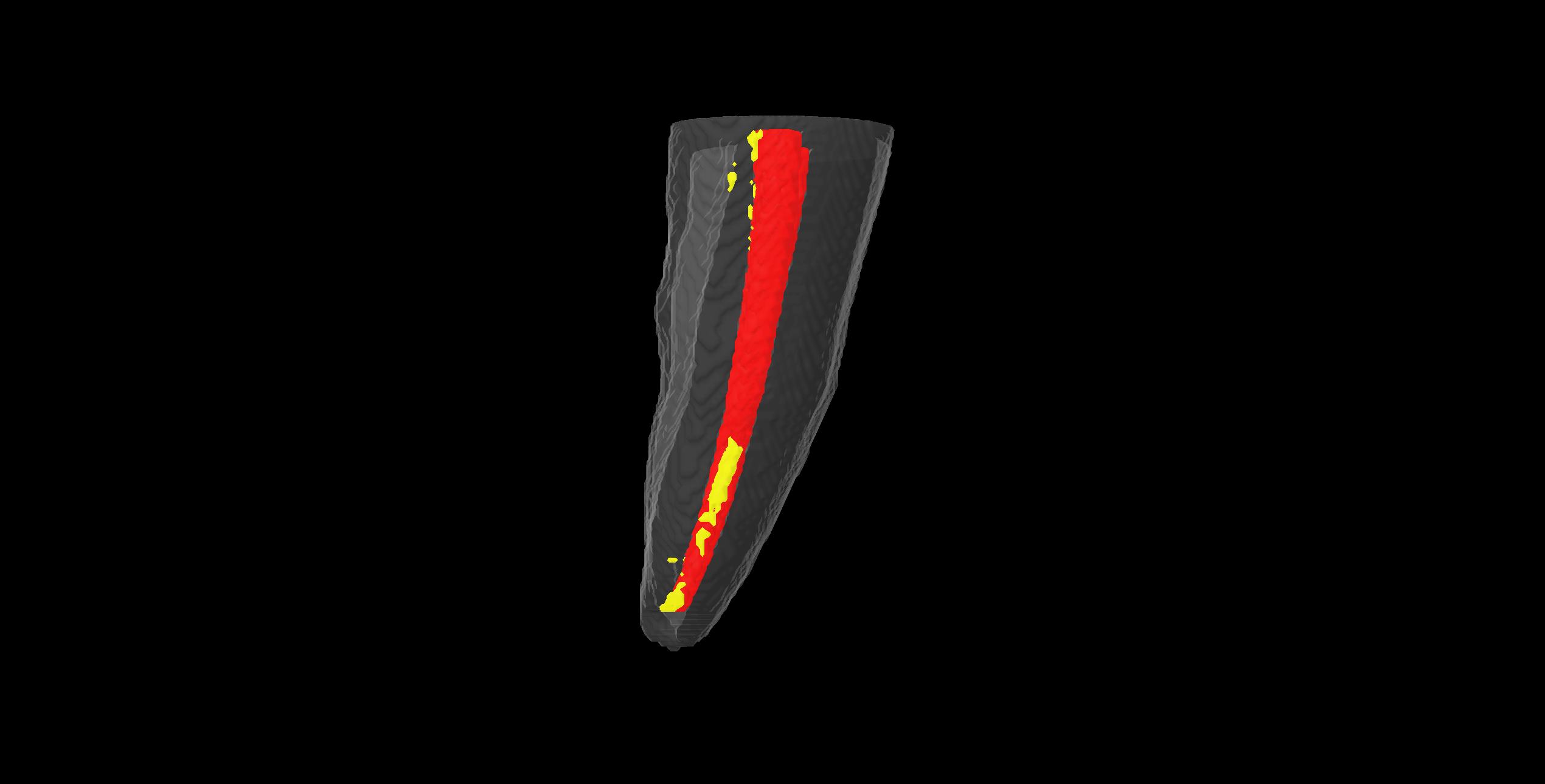

Supplement: S1 File — (ZIP) [file pone.0299896.s001.zip › Dra. Ola/Results & Images/28/28_buc_2.bmp]

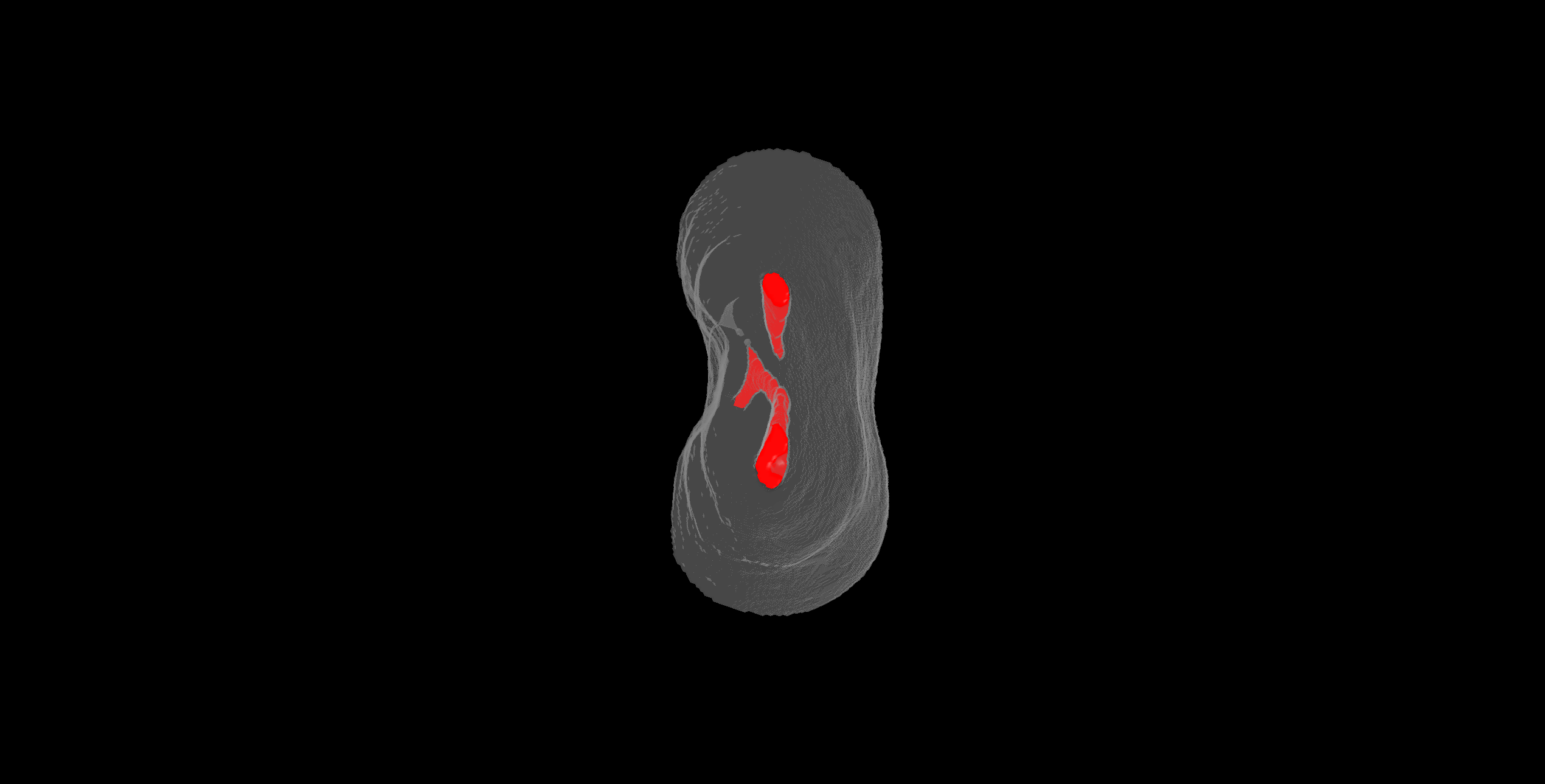

Supplement: S1 File — (ZIP) [file pone.0299896.s001.zip › Dra. Ola/Results & Images/28/28_cor.bmp]

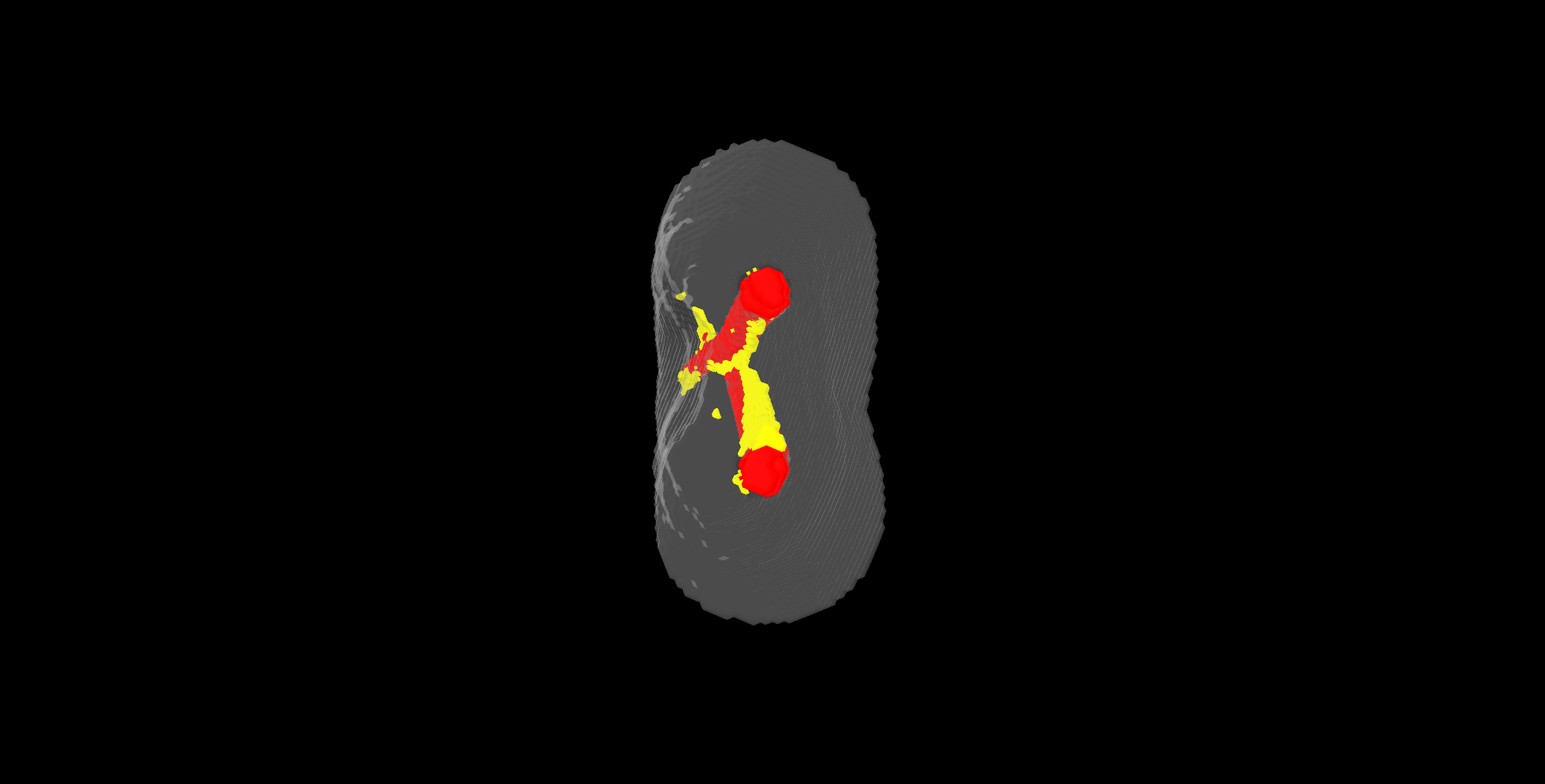

Supplement: S1 File — (ZIP) [file pone.0299896.s001.zip › Dra. Ola/Results & Images/28/28_cor_2.bmp]

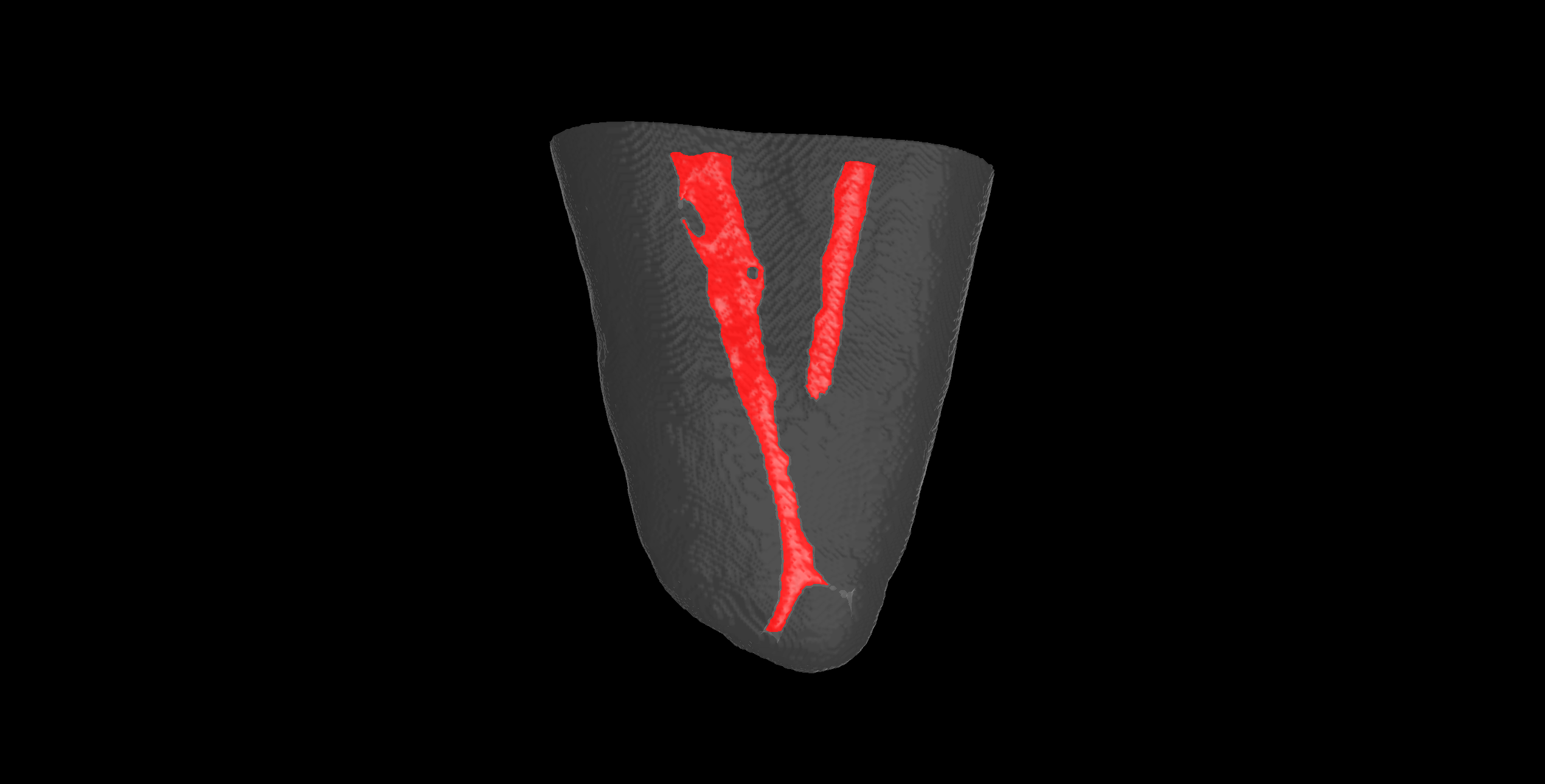

Supplement: S1 File — (ZIP) [file pone.0299896.s001.zip › Dra. Ola/Results & Images/28/28_mes.bmp]

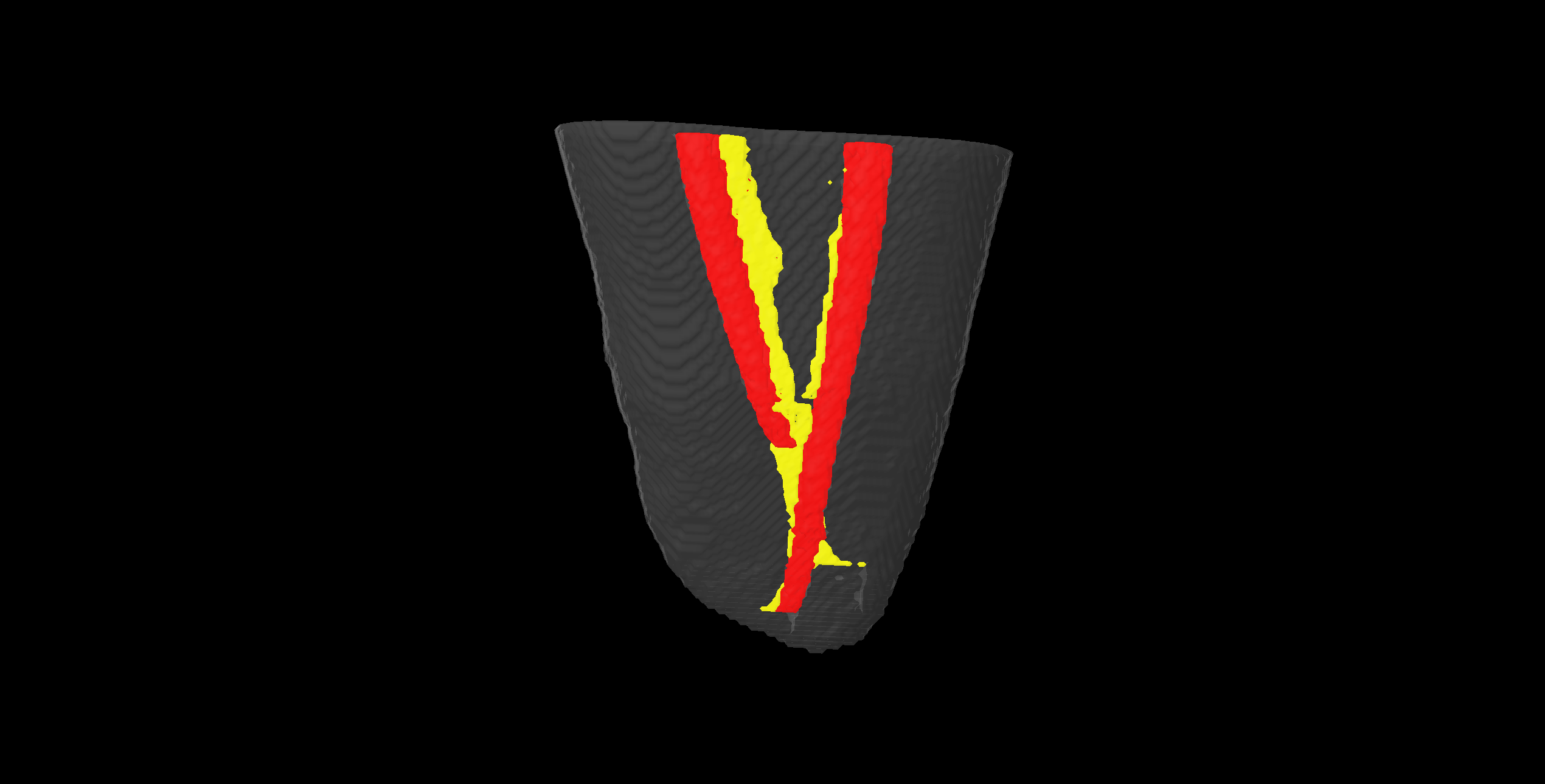

Supplement: S1 File — (ZIP) [file pone.0299896.s001.zip › Dra. Ola/Results & Images/28/28_mes_2.bmp]

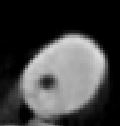

Supplement: S1 File — (ZIP) [file pone.0299896.s001.zip › Dra. Ola/Results & Images/29/1mm post.JPG]

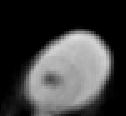

Supplement: S1 File — (ZIP) [file pone.0299896.s001.zip › Dra. Ola/Results & Images/29/1mm pre.JPG]
