# Supplementary figures and images for: Microcomputed tomography analysis of curved root canal preparation when coronal flaring and glide path files used with heat-treated nickel titanium rotary files (part 1 of 2)
Source: PLoS One. 2024 Apr 3;19(4):e0299896. doi: 10.1371/journal.pone.0299896 (PMC10990200; doi:10.1371/journal.pone.0299896)

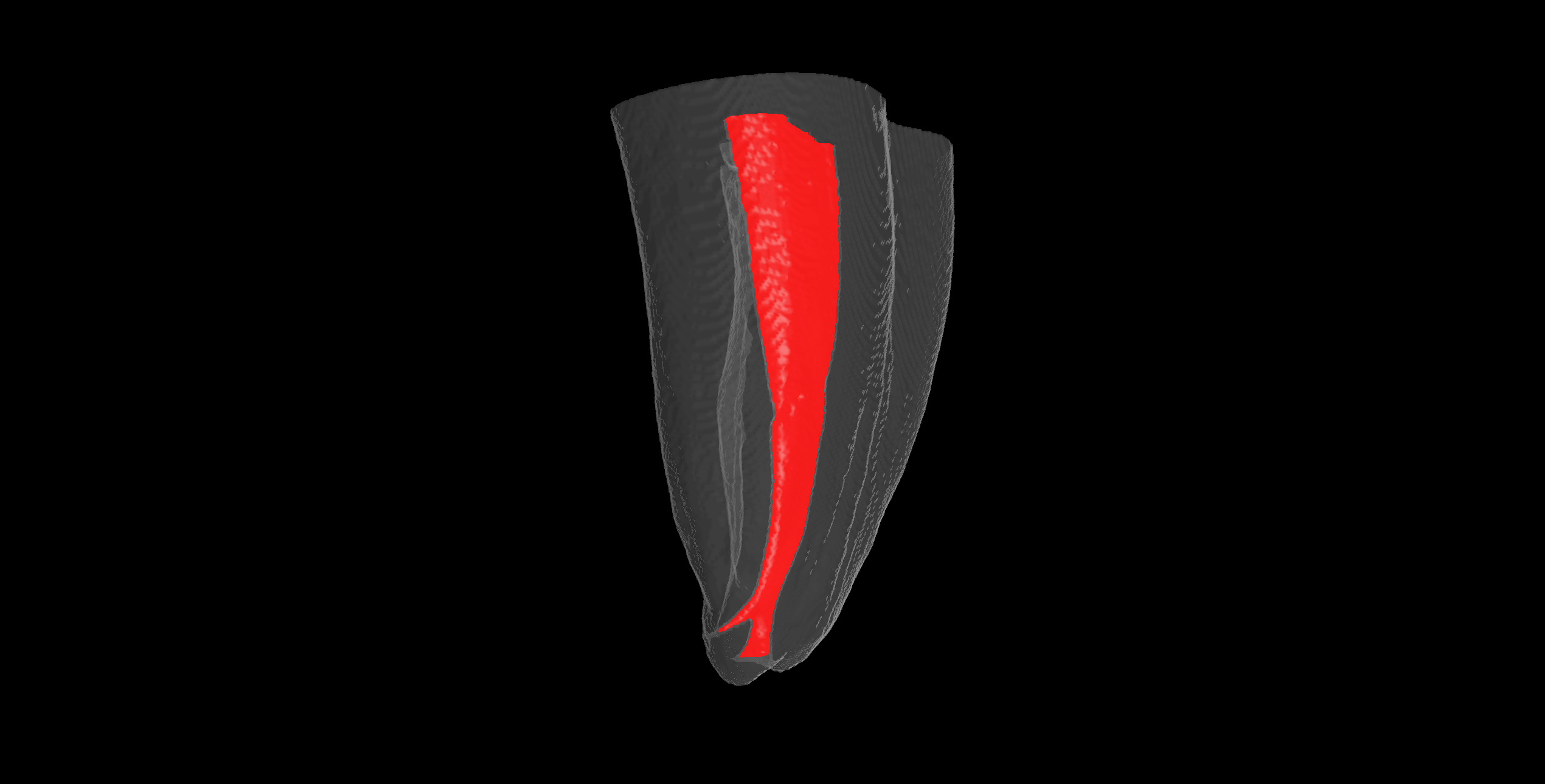

Supplement: S1 File — (ZIP) [file pone.0299896.s001.zip › Dra. Ola/Results & Images/10/10_buc.bmp]

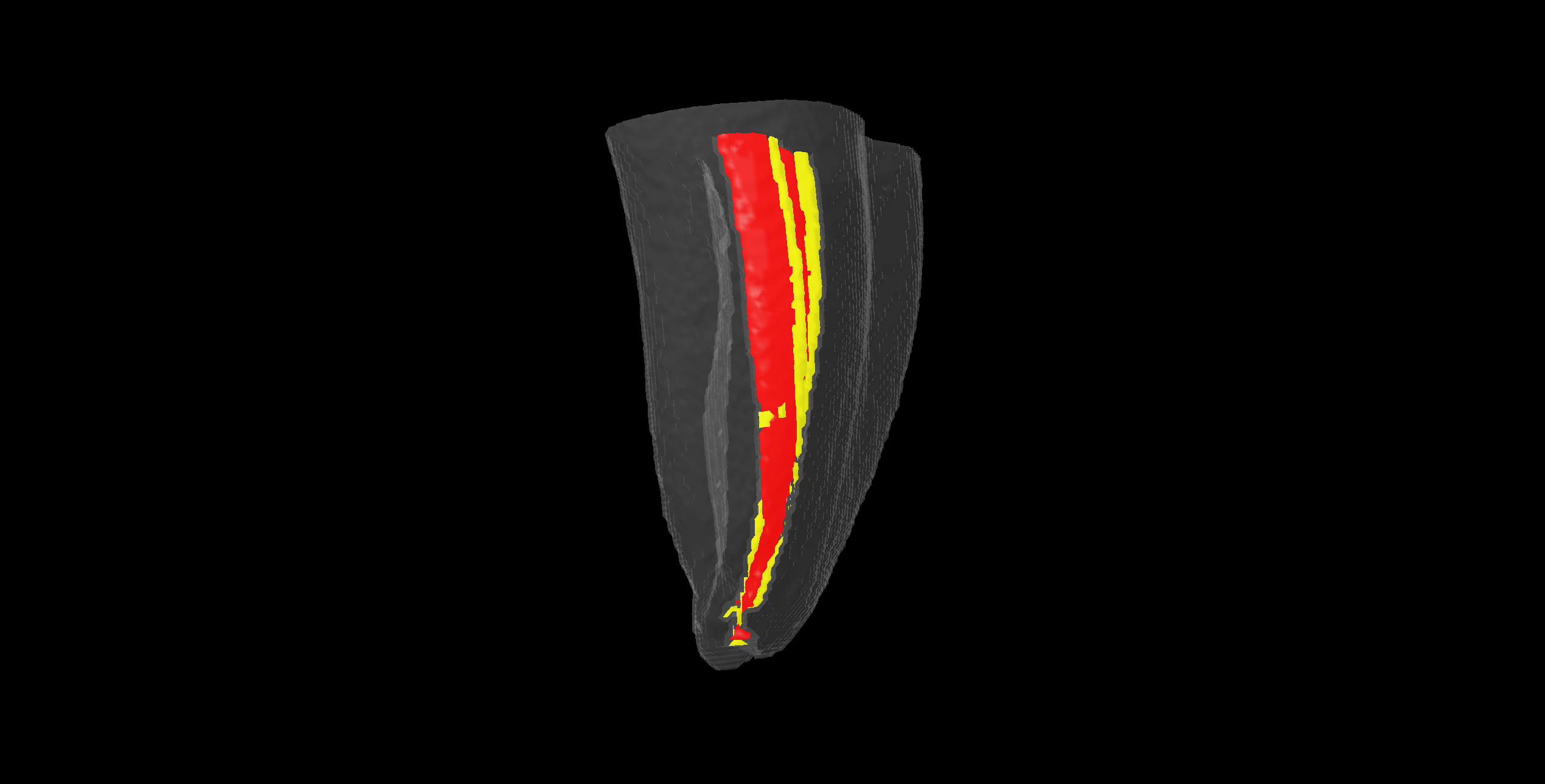

Supplement: S1 File — (ZIP) [file pone.0299896.s001.zip › Dra. Ola/Results & Images/10/10_buc2.bmp]

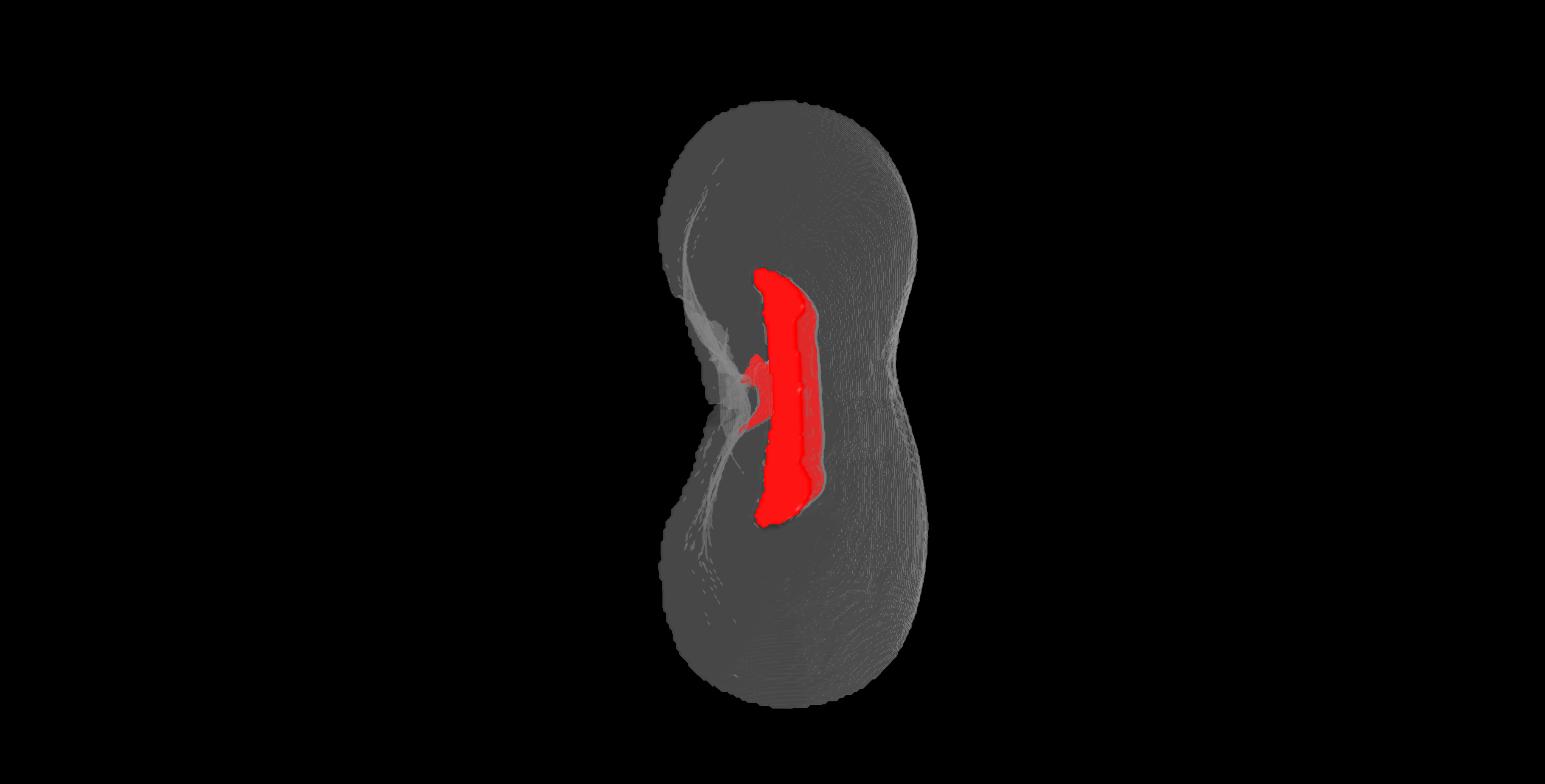

Supplement: S1 File — (ZIP) [file pone.0299896.s001.zip › Dra. Ola/Results & Images/10/10_cor.bmp]

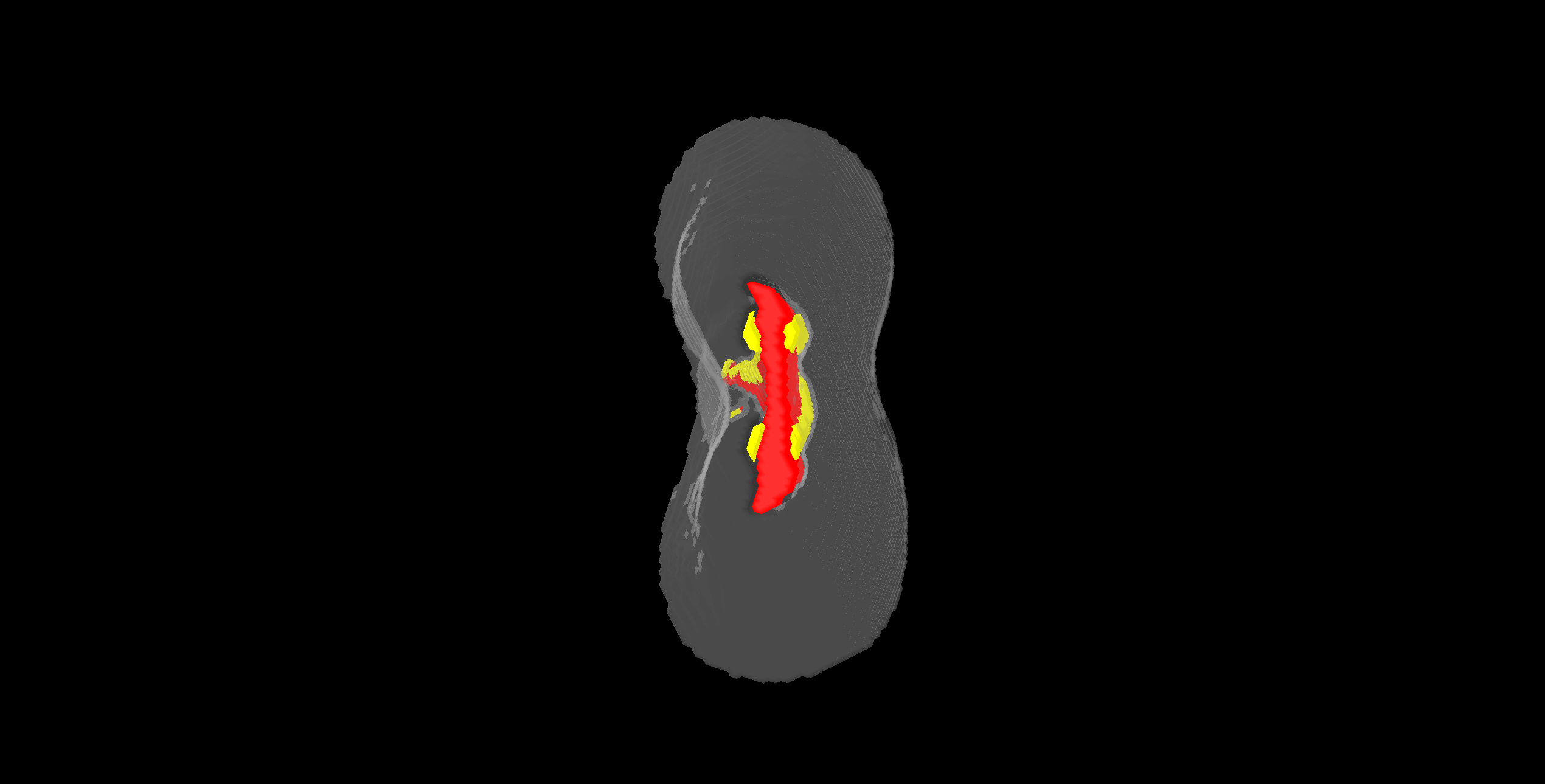

Supplement: S1 File — (ZIP) [file pone.0299896.s001.zip › Dra. Ola/Results & Images/10/10_cor2.bmp]

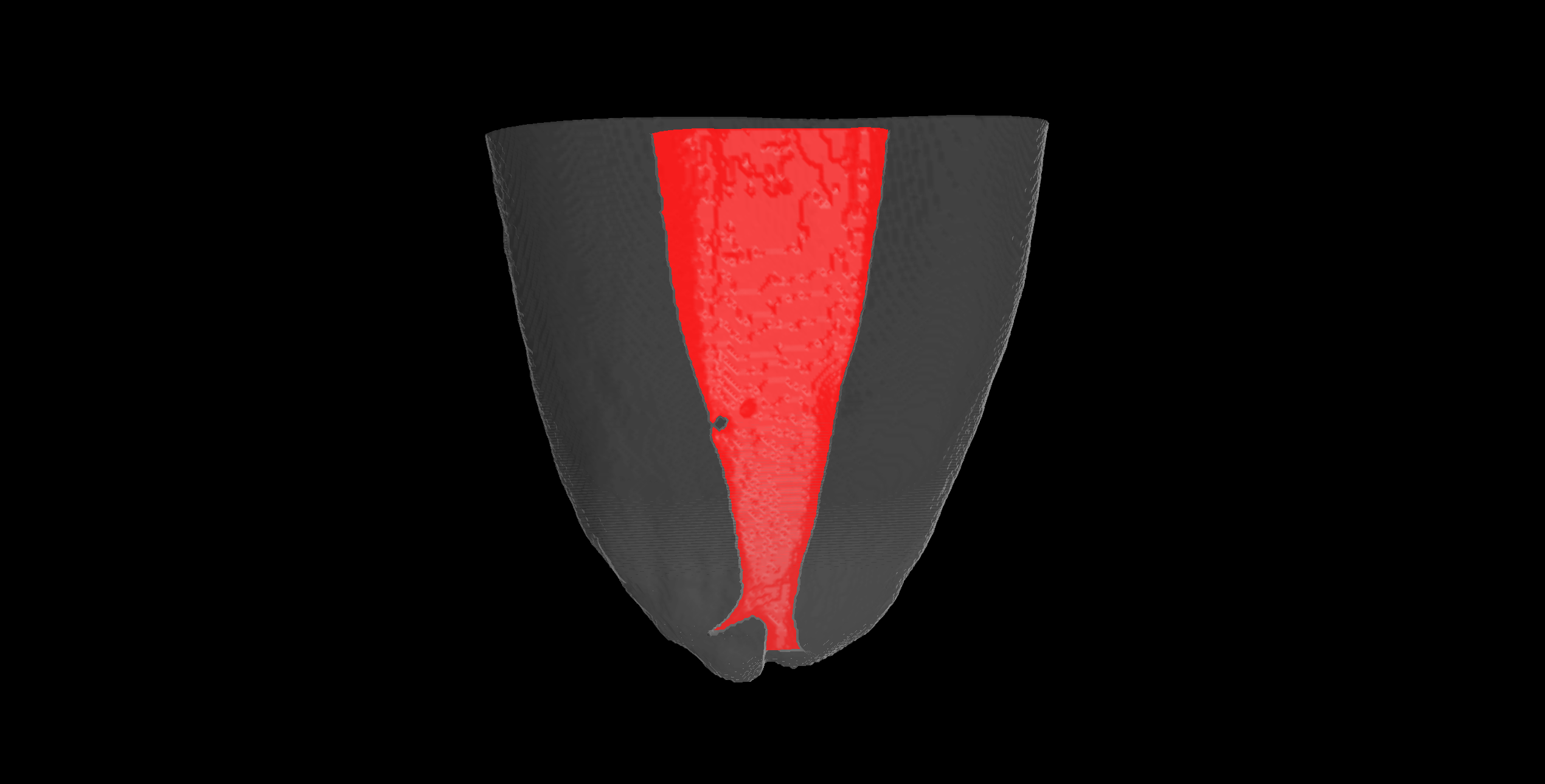

Supplement: S1 File — (ZIP) [file pone.0299896.s001.zip › Dra. Ola/Results & Images/10/10_mes.bmp]

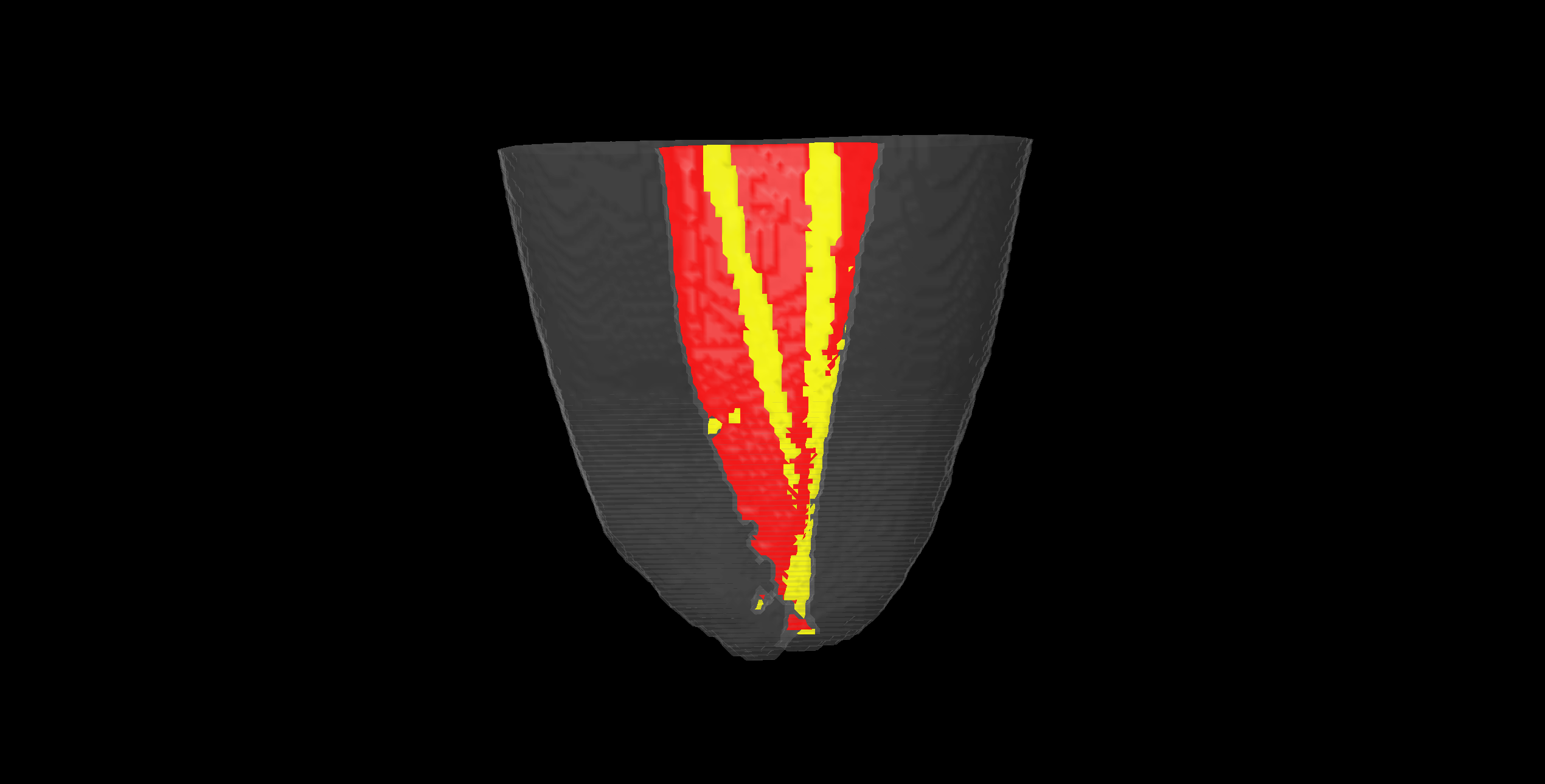

Supplement: S1 File — (ZIP) [file pone.0299896.s001.zip › Dra. Ola/Results & Images/10/10_mes2.bmp]

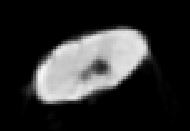

Supplement: S1 File — (ZIP) [file pone.0299896.s001.zip › Dra. Ola/Results & Images/10/1mm post.JPG]

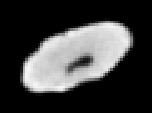

Supplement: S1 File — (ZIP) [file pone.0299896.s001.zip › Dra. Ola/Results & Images/10/1mm pre.JPG]

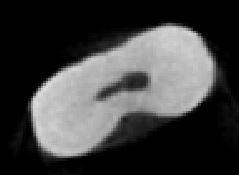

Supplement: S1 File — (ZIP) [file pone.0299896.s001.zip › Dra. Ola/Results & Images/10/3mm post.JPG]

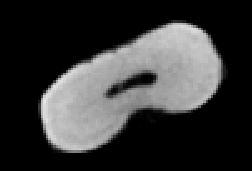

Supplement: S1 File — (ZIP) [file pone.0299896.s001.zip › Dra. Ola/Results & Images/10/3mm pre.JPG]

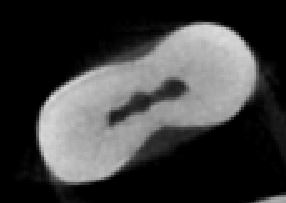

Supplement: S1 File — (ZIP) [file pone.0299896.s001.zip › Dra. Ola/Results & Images/10/5mm post.JPG]

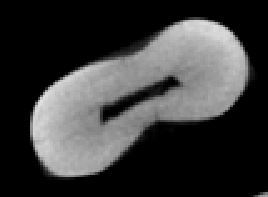

Supplement: S1 File — (ZIP) [file pone.0299896.s001.zip › Dra. Ola/Results & Images/10/5mm pre.JPG]

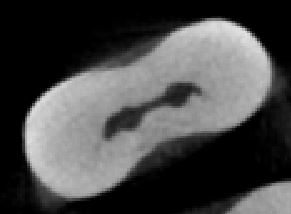

Supplement: S1 File — (ZIP) [file pone.0299896.s001.zip › Dra. Ola/Results & Images/10/7mm post.JPG]

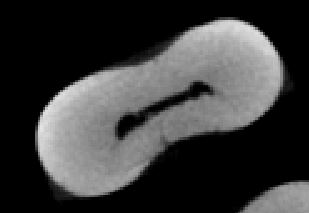

Supplement: S1 File — (ZIP) [file pone.0299896.s001.zip › Dra. Ola/Results & Images/10/7mm pre.JPG]

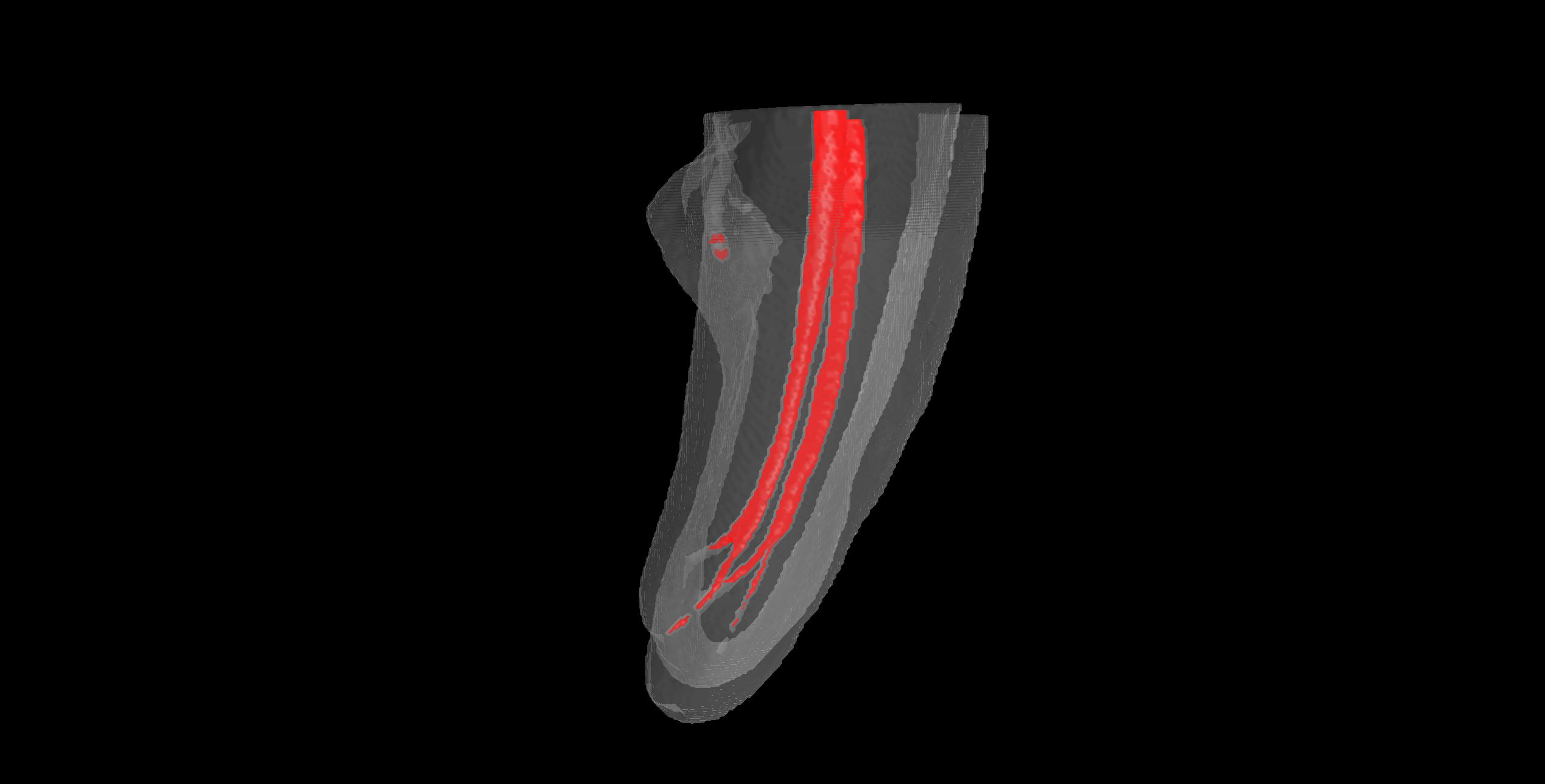

Supplement: S1 File — (ZIP) [file pone.0299896.s001.zip › Dra. Ola/Results & Images/12/12_buc.bmp]

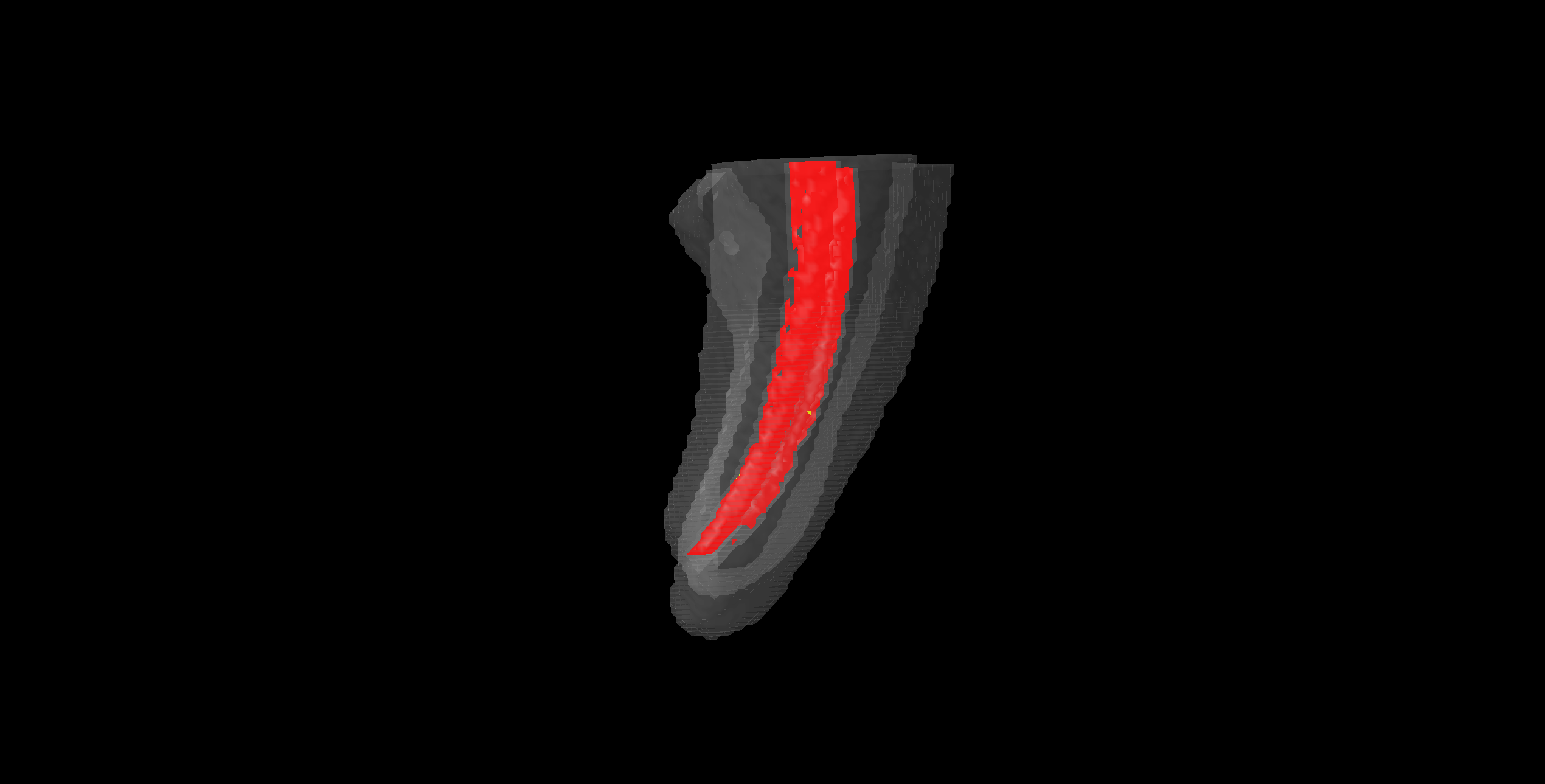

Supplement: S1 File — (ZIP) [file pone.0299896.s001.zip › Dra. Ola/Results & Images/12/12_buc2.bmp]

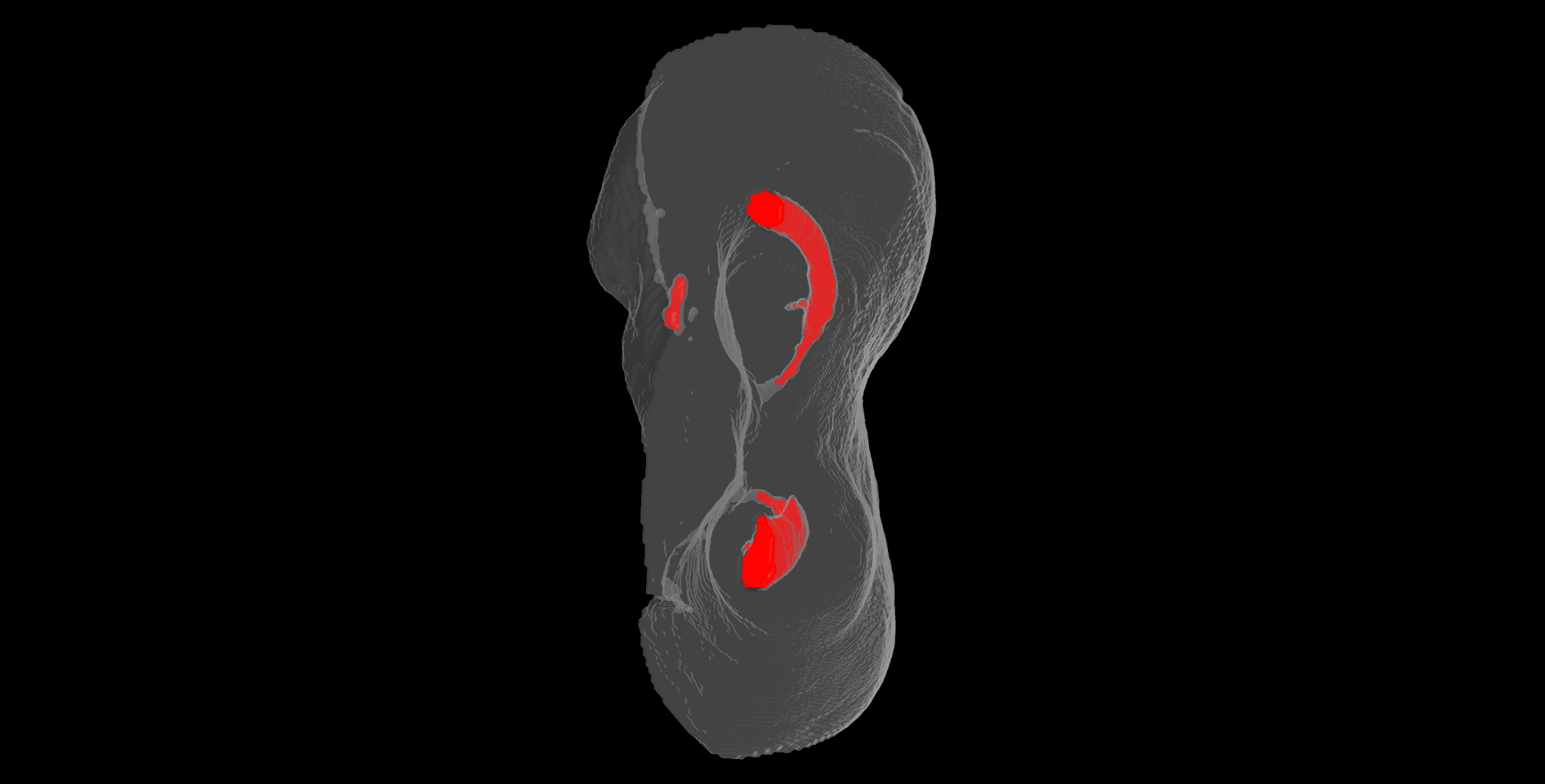

Supplement: S1 File — (ZIP) [file pone.0299896.s001.zip › Dra. Ola/Results & Images/12/12_cor.bmp]

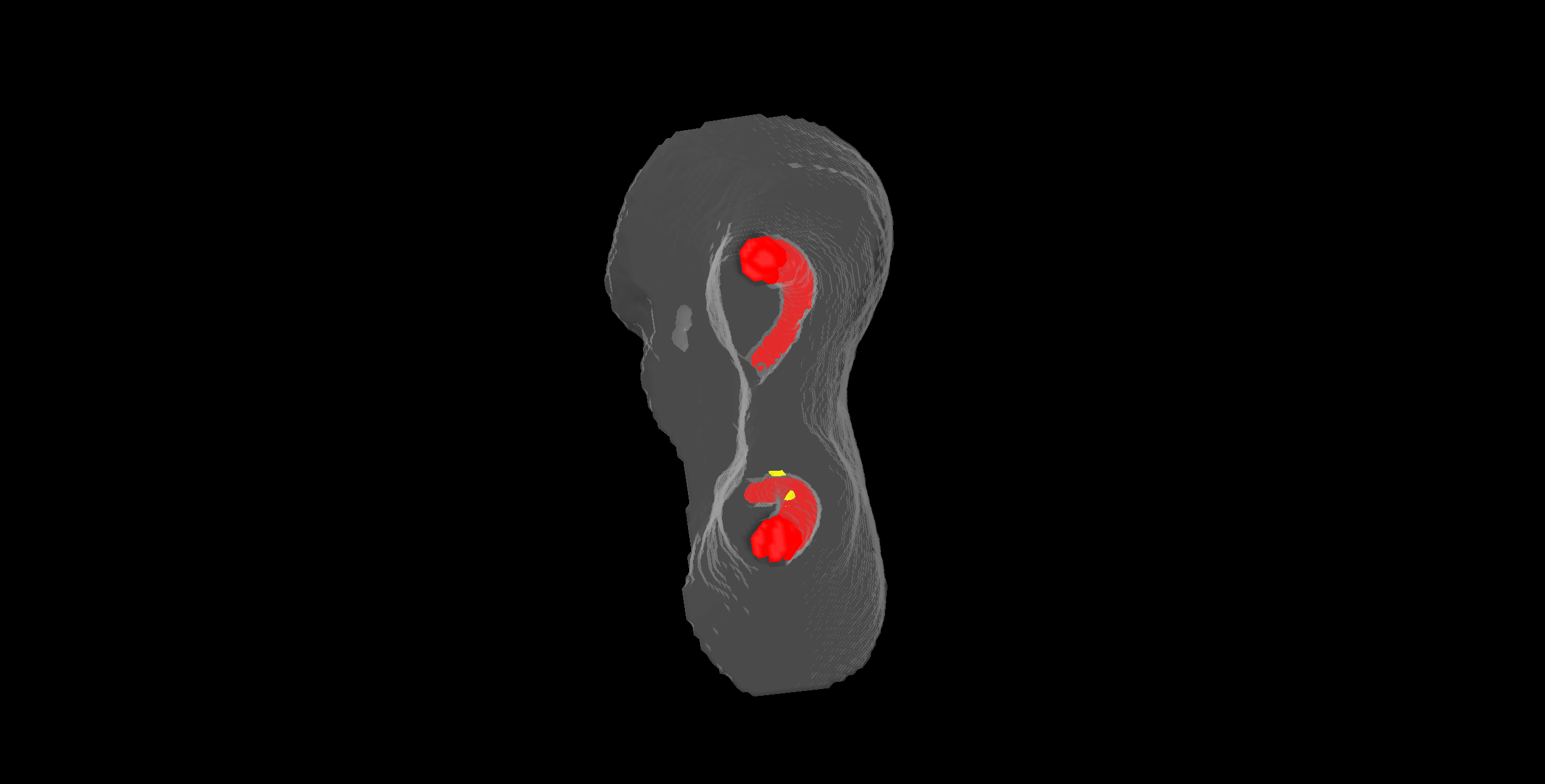

Supplement: S1 File — (ZIP) [file pone.0299896.s001.zip › Dra. Ola/Results & Images/12/12_cor2.bmp]

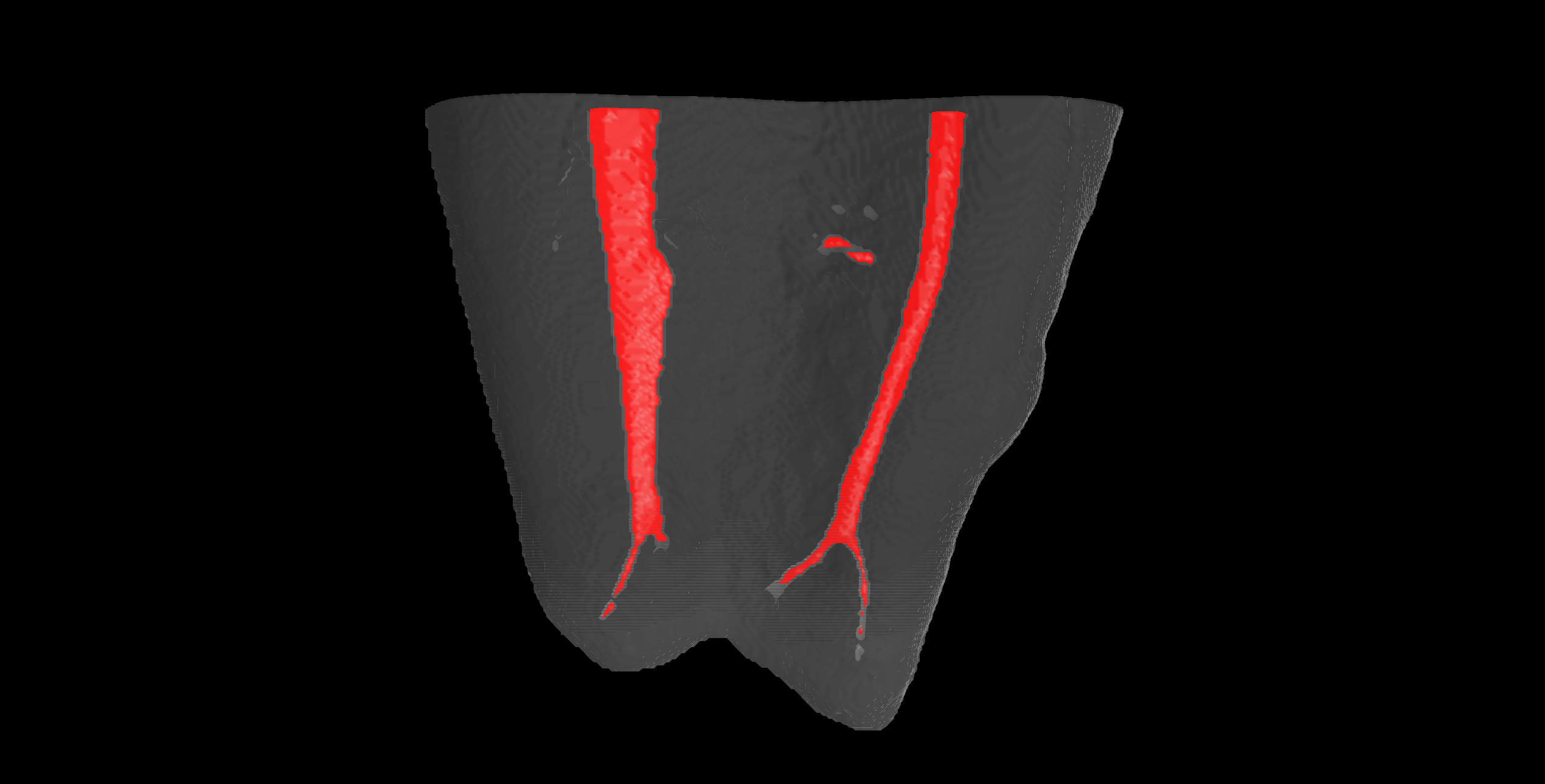

Supplement: S1 File — (ZIP) [file pone.0299896.s001.zip › Dra. Ola/Results & Images/12/12_mes.bmp]

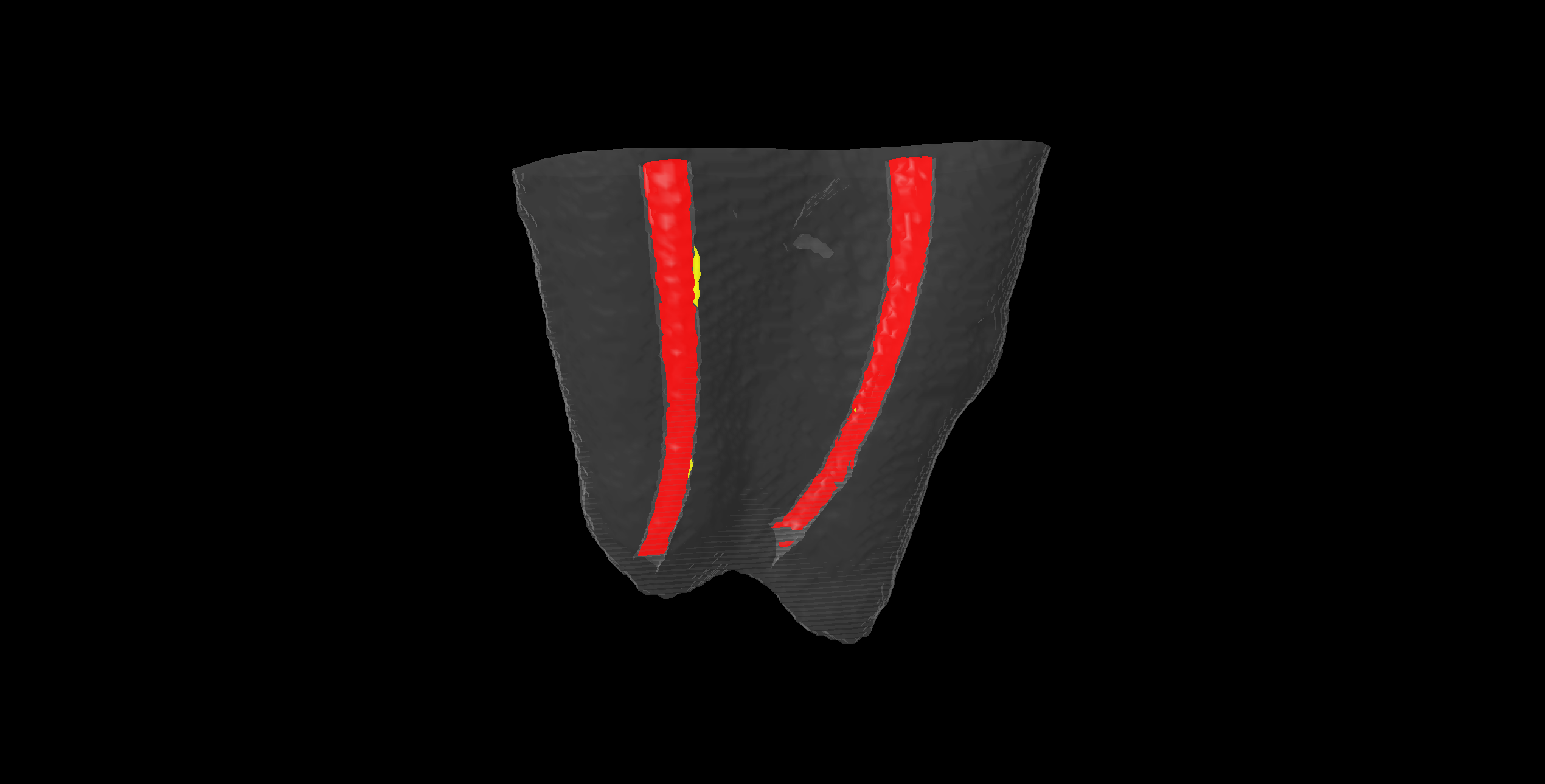

Supplement: S1 File — (ZIP) [file pone.0299896.s001.zip › Dra. Ola/Results & Images/12/12_mes2.bmp]

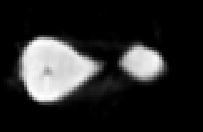

Supplement: S1 File — (ZIP) [file pone.0299896.s001.zip › Dra. Ola/Results & Images/12/1mm post.JPG]

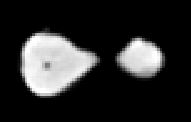

Supplement: S1 File — (ZIP) [file pone.0299896.s001.zip › Dra. Ola/Results & Images/12/1mm pre.JPG]

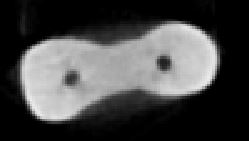

Supplement: S1 File — (ZIP) [file pone.0299896.s001.zip › Dra. Ola/Results & Images/12/3mm post.JPG]

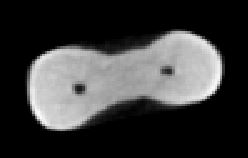

Supplement: S1 File — (ZIP) [file pone.0299896.s001.zip › Dra. Ola/Results & Images/12/3mm pre.JPG]

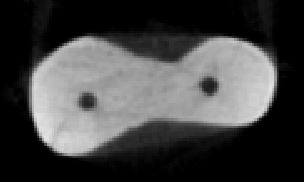

Supplement: S1 File — (ZIP) [file pone.0299896.s001.zip › Dra. Ola/Results & Images/12/5mm post.JPG]

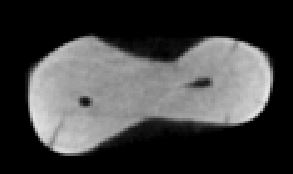

Supplement: S1 File — (ZIP) [file pone.0299896.s001.zip › Dra. Ola/Results & Images/12/5mm pre.JPG]

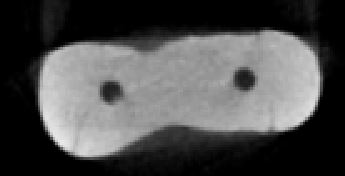

Supplement: S1 File — (ZIP) [file pone.0299896.s001.zip › Dra. Ola/Results & Images/12/7mm post.JPG]

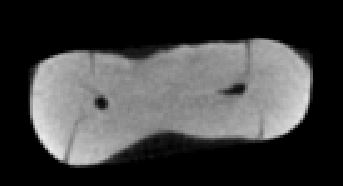

Supplement: S1 File — (ZIP) [file pone.0299896.s001.zip › Dra. Ola/Results & Images/12/7mm pre.JPG]

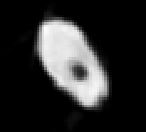

Supplement: S1 File — (ZIP) [file pone.0299896.s001.zip › Dra. Ola/Results & Images/15/1mm post.JPG]

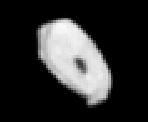

Supplement: S1 File — (ZIP) [file pone.0299896.s001.zip › Dra. Ola/Results & Images/15/1mm pre.JPG]

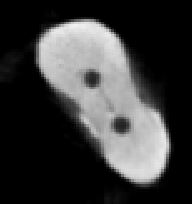

Supplement: S1 File — (ZIP) [file pone.0299896.s001.zip › Dra. Ola/Results & Images/15/3mm post.JPG]

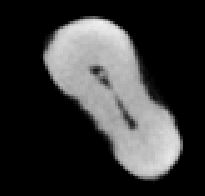

Supplement: S1 File — (ZIP) [file pone.0299896.s001.zip › Dra. Ola/Results & Images/15/3mm pre.JPG]

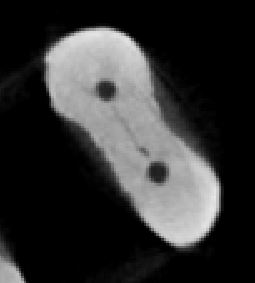

Supplement: S1 File — (ZIP) [file pone.0299896.s001.zip › Dra. Ola/Results & Images/15/5mm post.JPG]

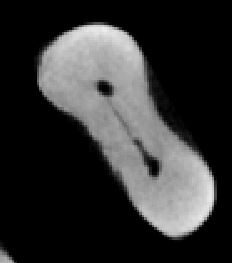

Supplement: S1 File — (ZIP) [file pone.0299896.s001.zip › Dra. Ola/Results & Images/15/5mm pre.JPG]

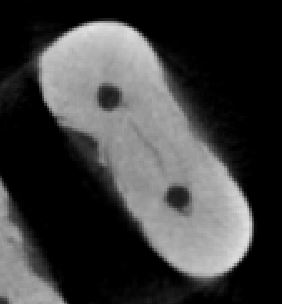

Supplement: S1 File — (ZIP) [file pone.0299896.s001.zip › Dra. Ola/Results & Images/15/7mm post.JPG]

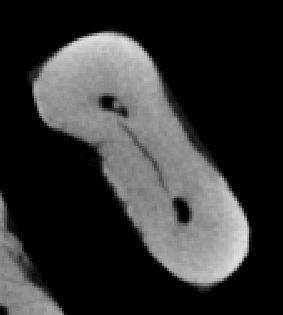

Supplement: S1 File — (ZIP) [file pone.0299896.s001.zip › Dra. Ola/Results & Images/15/7mm pre.JPG]

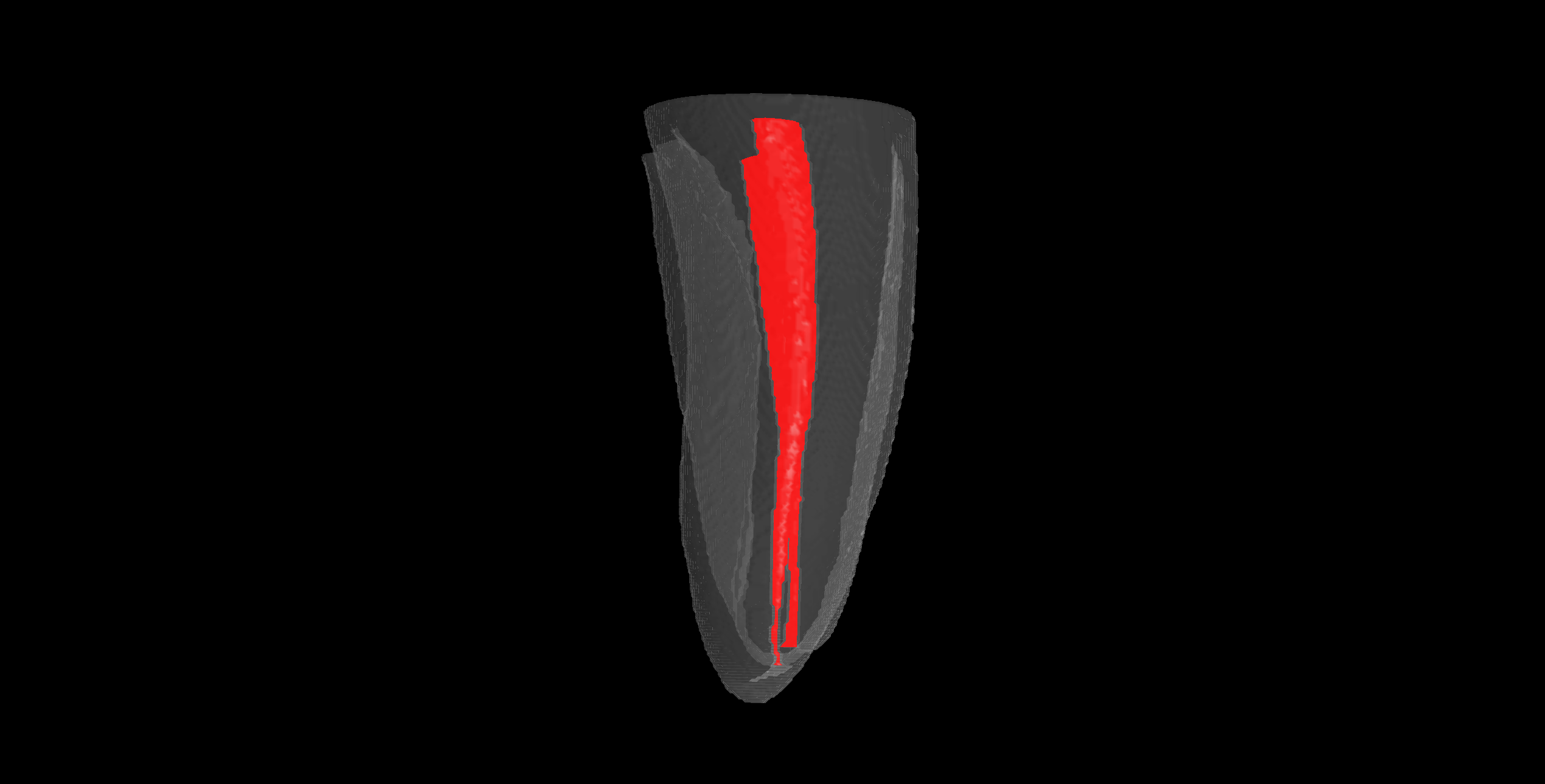

Supplement: S1 File — (ZIP) [file pone.0299896.s001.zip › Dra. Ola/Results & Images/16/16_buc.bmp]

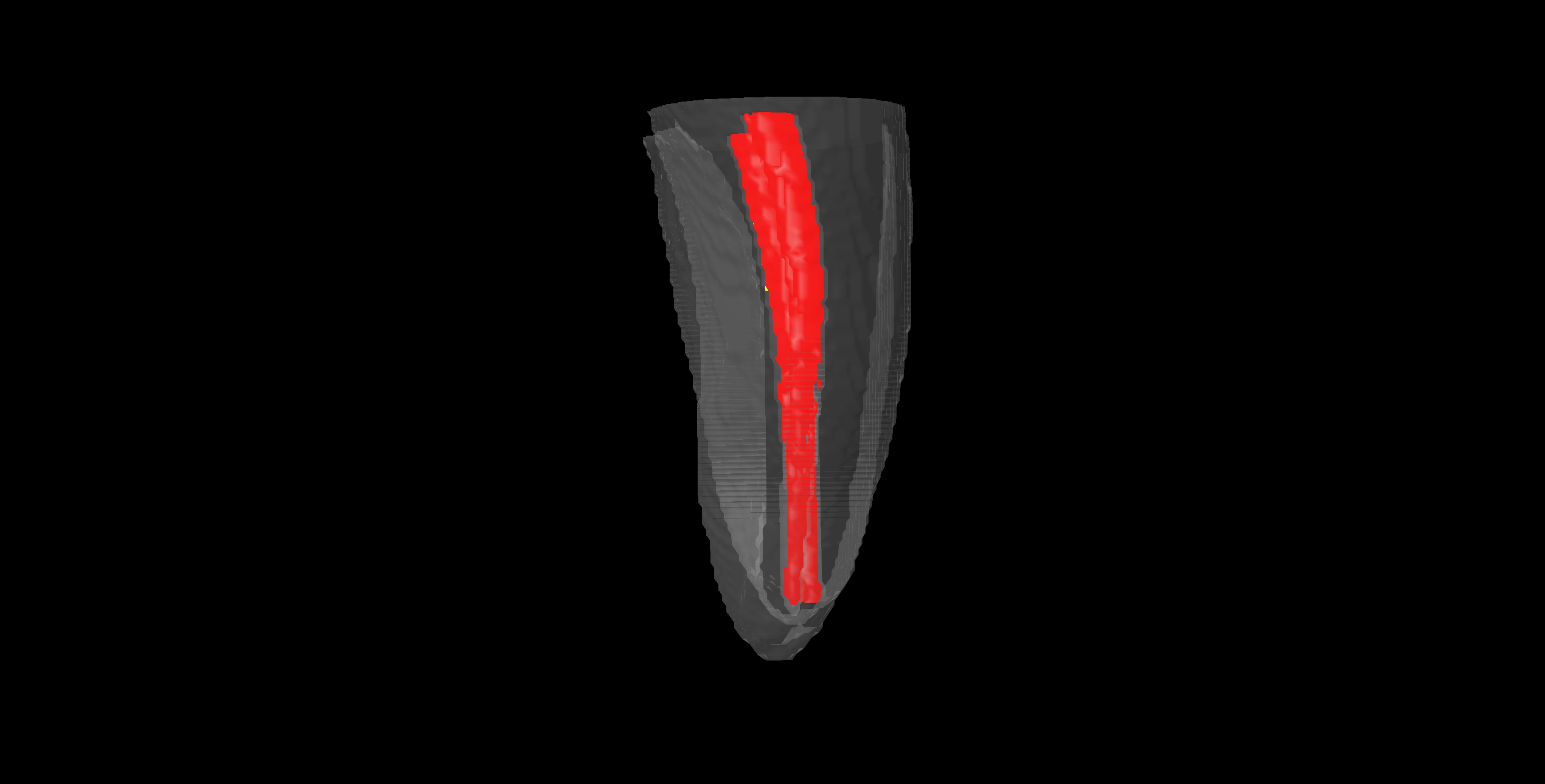

Supplement: S1 File — (ZIP) [file pone.0299896.s001.zip › Dra. Ola/Results & Images/16/16_buc2.bmp]

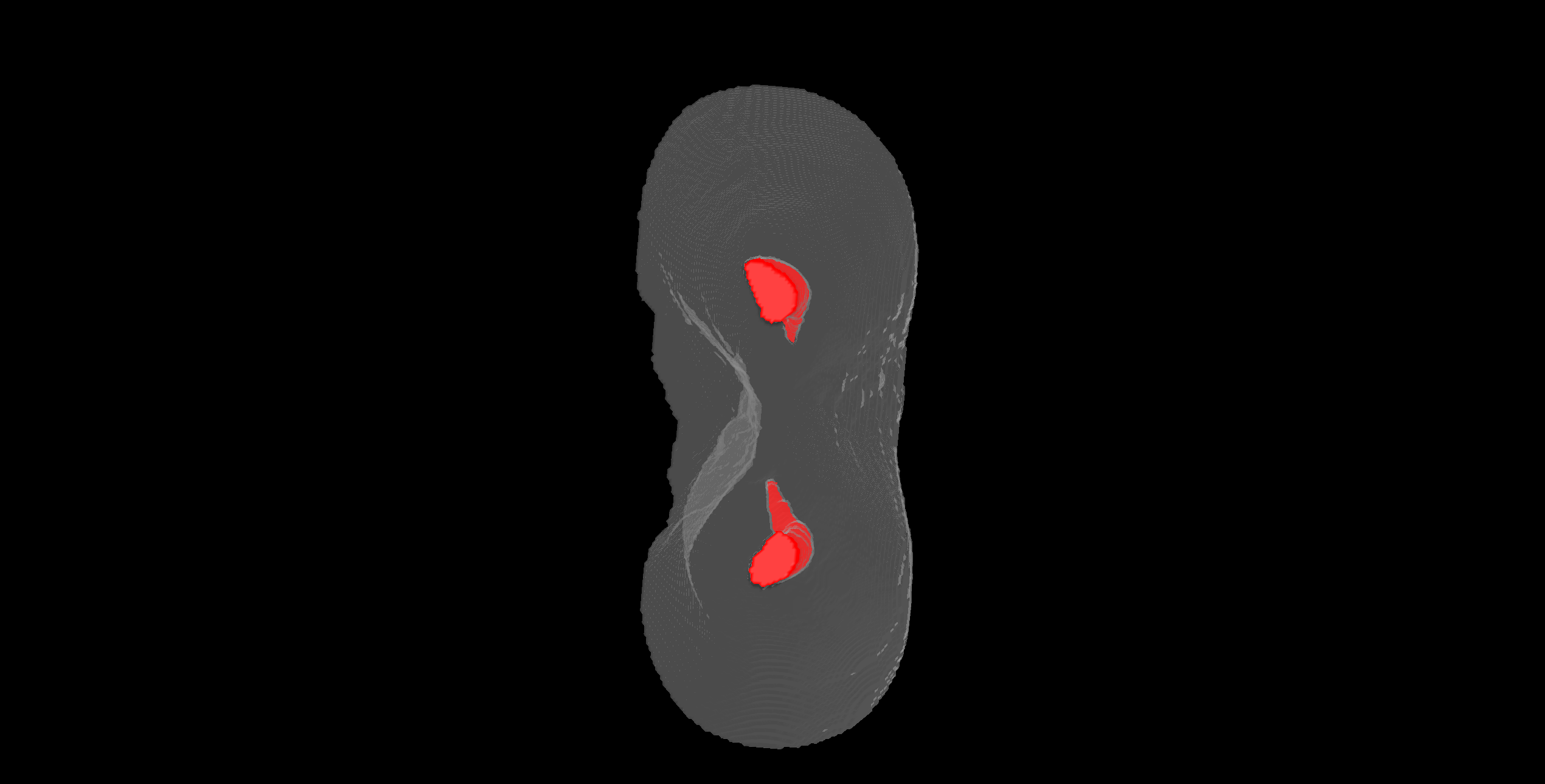

Supplement: S1 File — (ZIP) [file pone.0299896.s001.zip › Dra. Ola/Results & Images/16/16_cor.bmp]

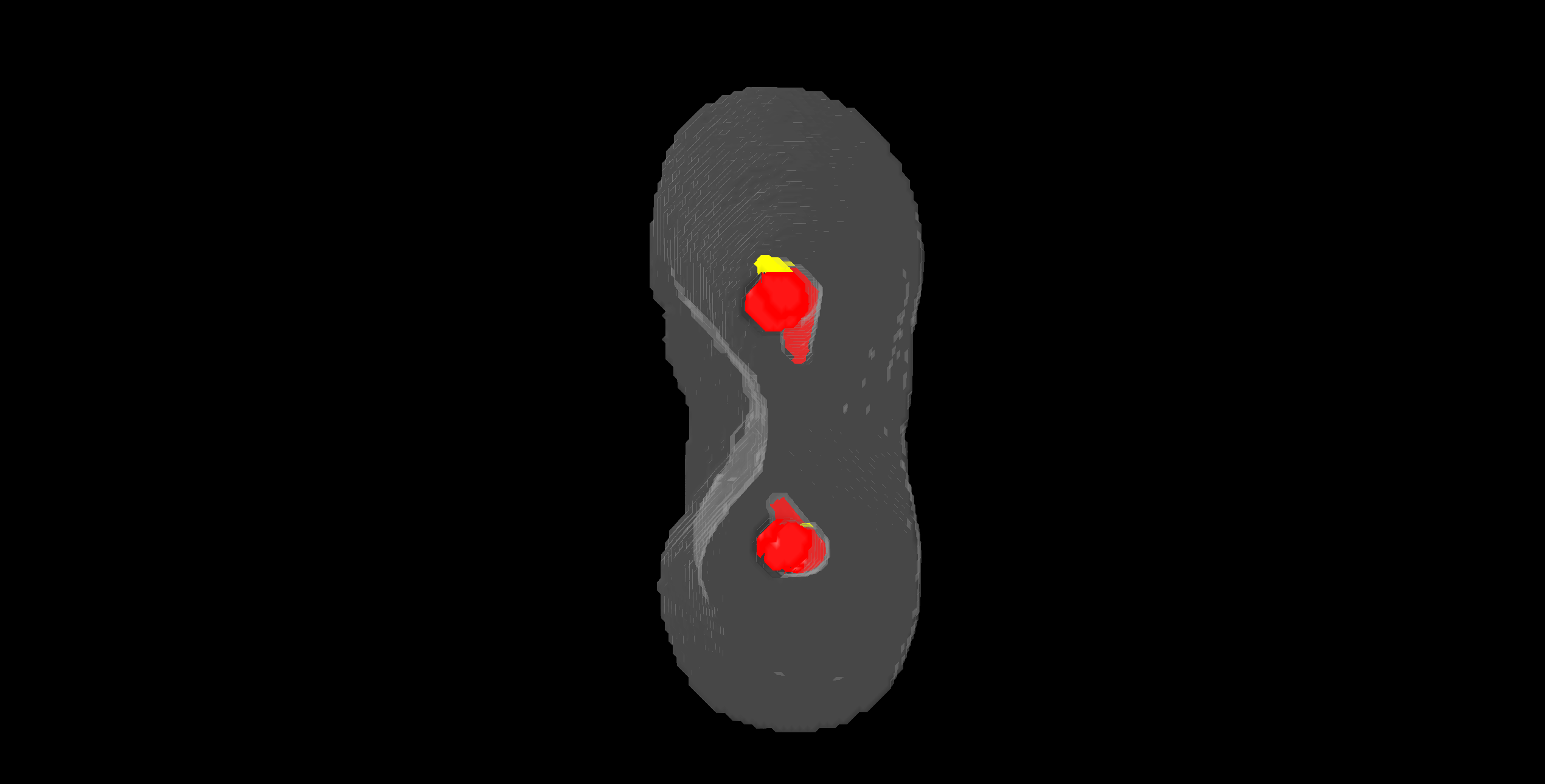

Supplement: S1 File — (ZIP) [file pone.0299896.s001.zip › Dra. Ola/Results & Images/16/16_cor2.bmp]

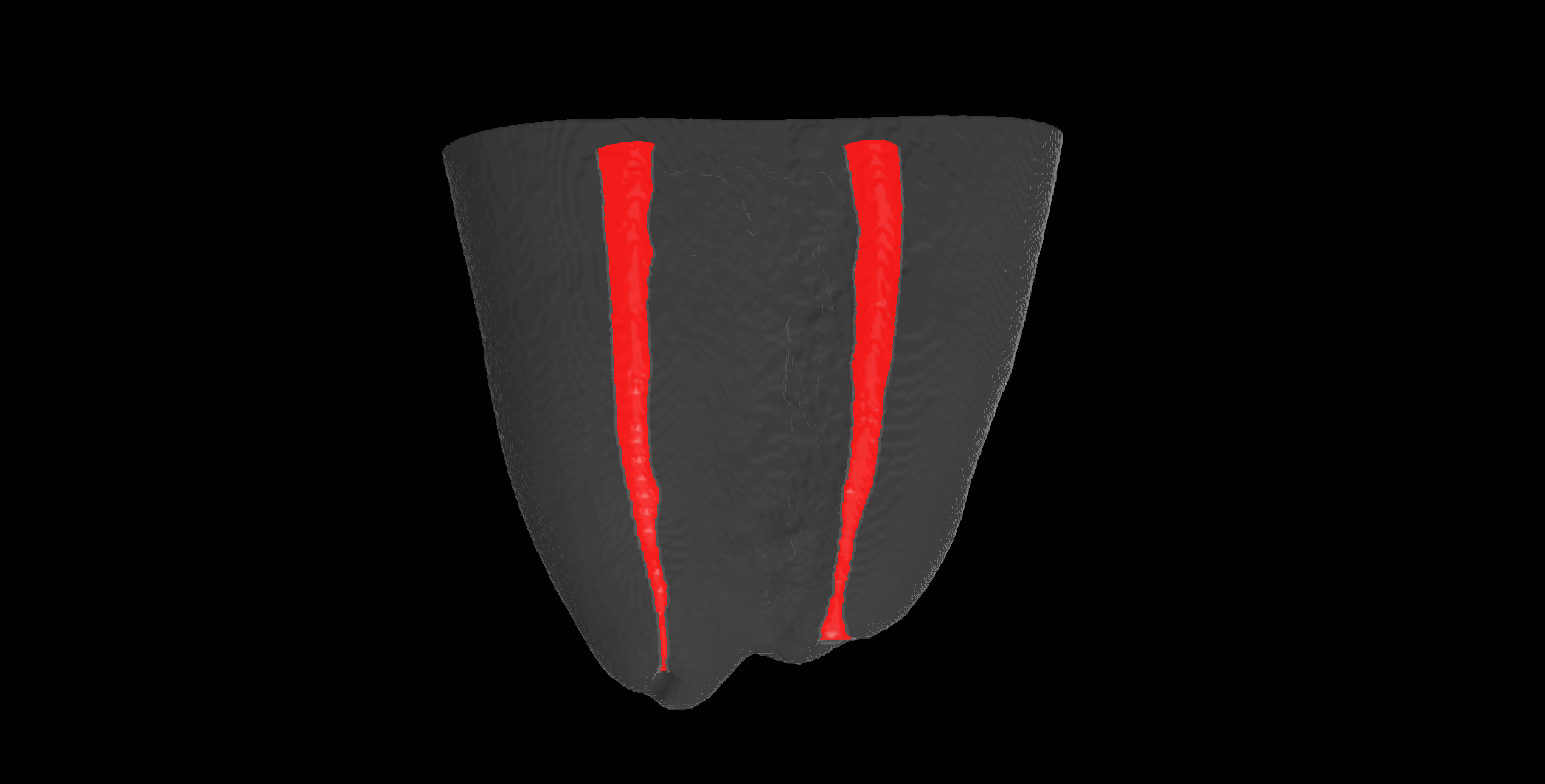

Supplement: S1 File — (ZIP) [file pone.0299896.s001.zip › Dra. Ola/Results & Images/16/16_mes.bmp]

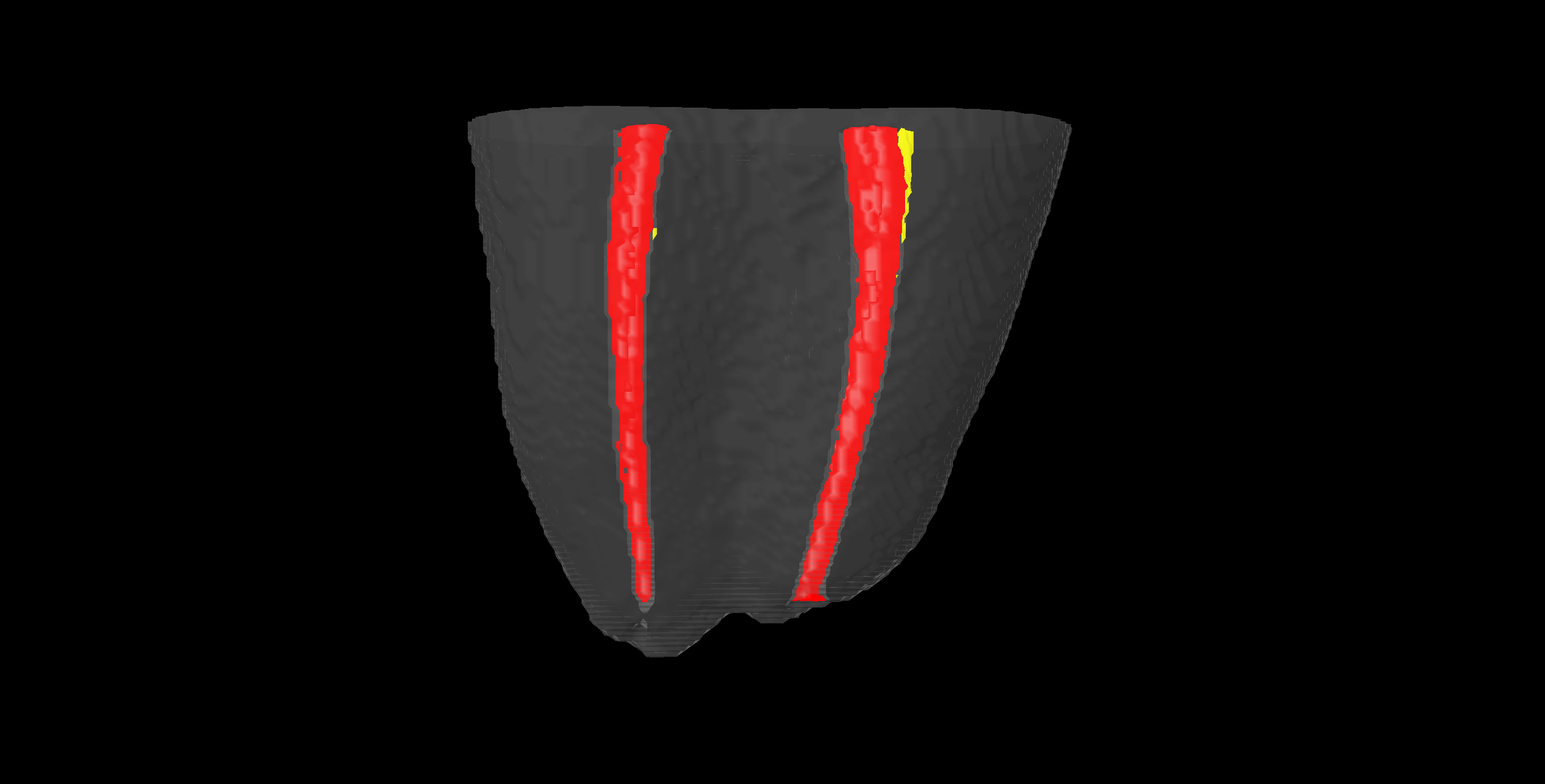

Supplement: S1 File — (ZIP) [file pone.0299896.s001.zip › Dra. Ola/Results & Images/16/16_mes2.bmp]

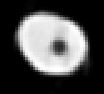

Supplement: S1 File — (ZIP) [file pone.0299896.s001.zip › Dra. Ola/Results & Images/17/1mm post.JPG]

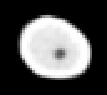

Supplement: S1 File — (ZIP) [file pone.0299896.s001.zip › Dra. Ola/Results & Images/17/1mm pre.JPG]

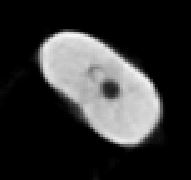

Supplement: S1 File — (ZIP) [file pone.0299896.s001.zip › Dra. Ola/Results & Images/17/3mm post.JPG]

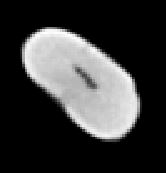

Supplement: S1 File — (ZIP) [file pone.0299896.s001.zip › Dra. Ola/Results & Images/17/3mm pre.JPG]

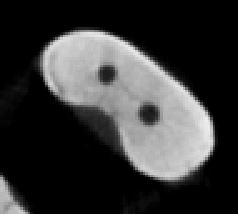

Supplement: S1 File — (ZIP) [file pone.0299896.s001.zip › Dra. Ola/Results & Images/17/5mm post.JPG]

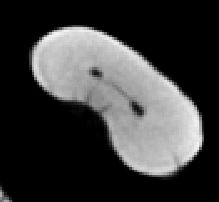

Supplement: S1 File — (ZIP) [file pone.0299896.s001.zip › Dra. Ola/Results & Images/17/5mm pre.JPG]

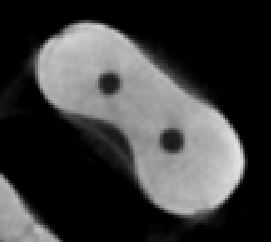

Supplement: S1 File — (ZIP) [file pone.0299896.s001.zip › Dra. Ola/Results & Images/17/7mm post.JPG]

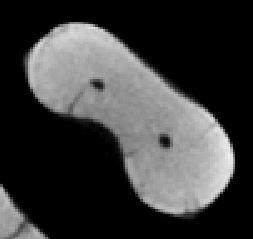

Supplement: S1 File — (ZIP) [file pone.0299896.s001.zip › Dra. Ola/Results & Images/17/7mm pre.JPG]

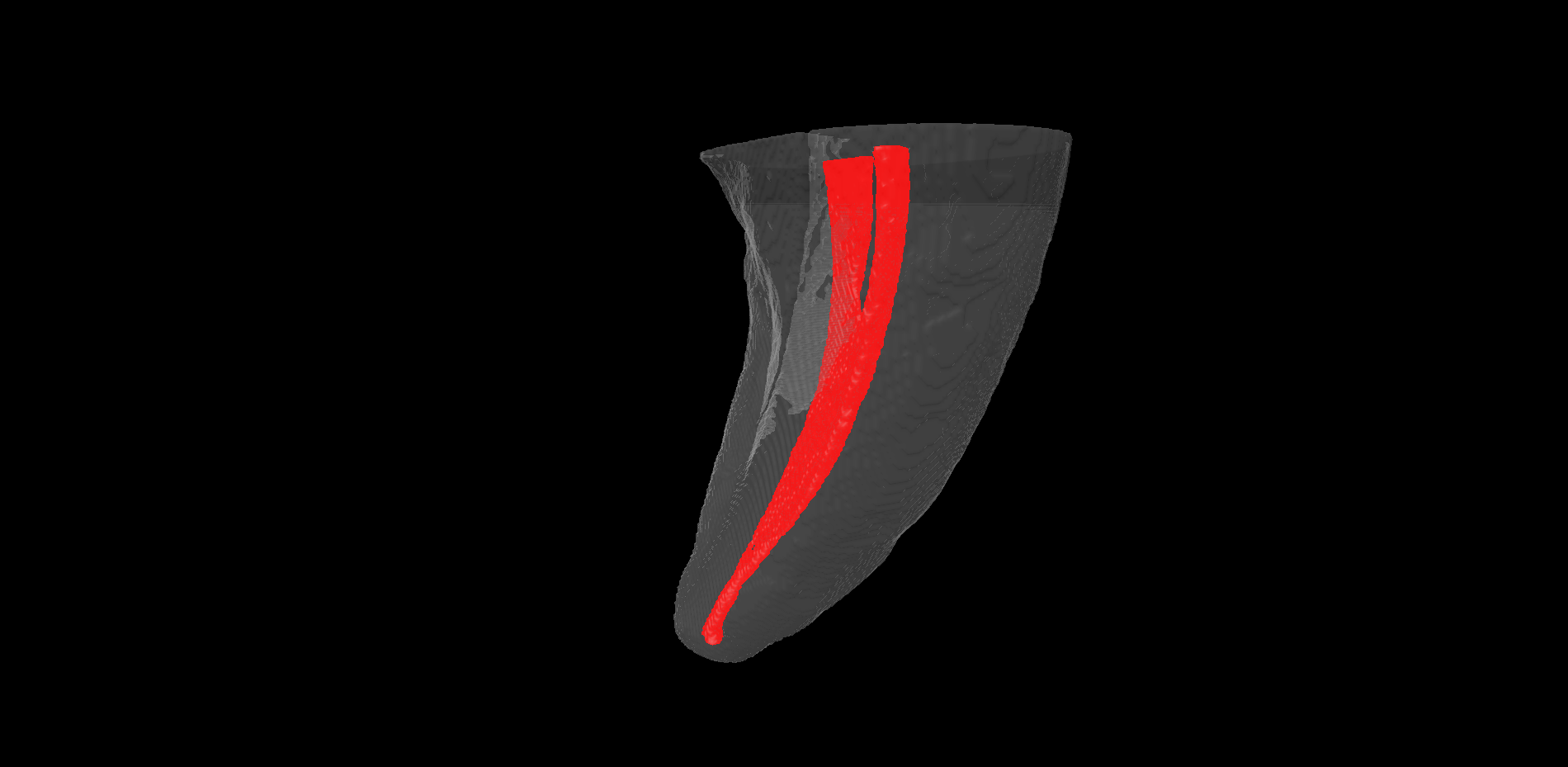

Supplement: S1 File — (ZIP) [file pone.0299896.s001.zip › Dra. Ola/Results & Images/18/18_buc.bmp]

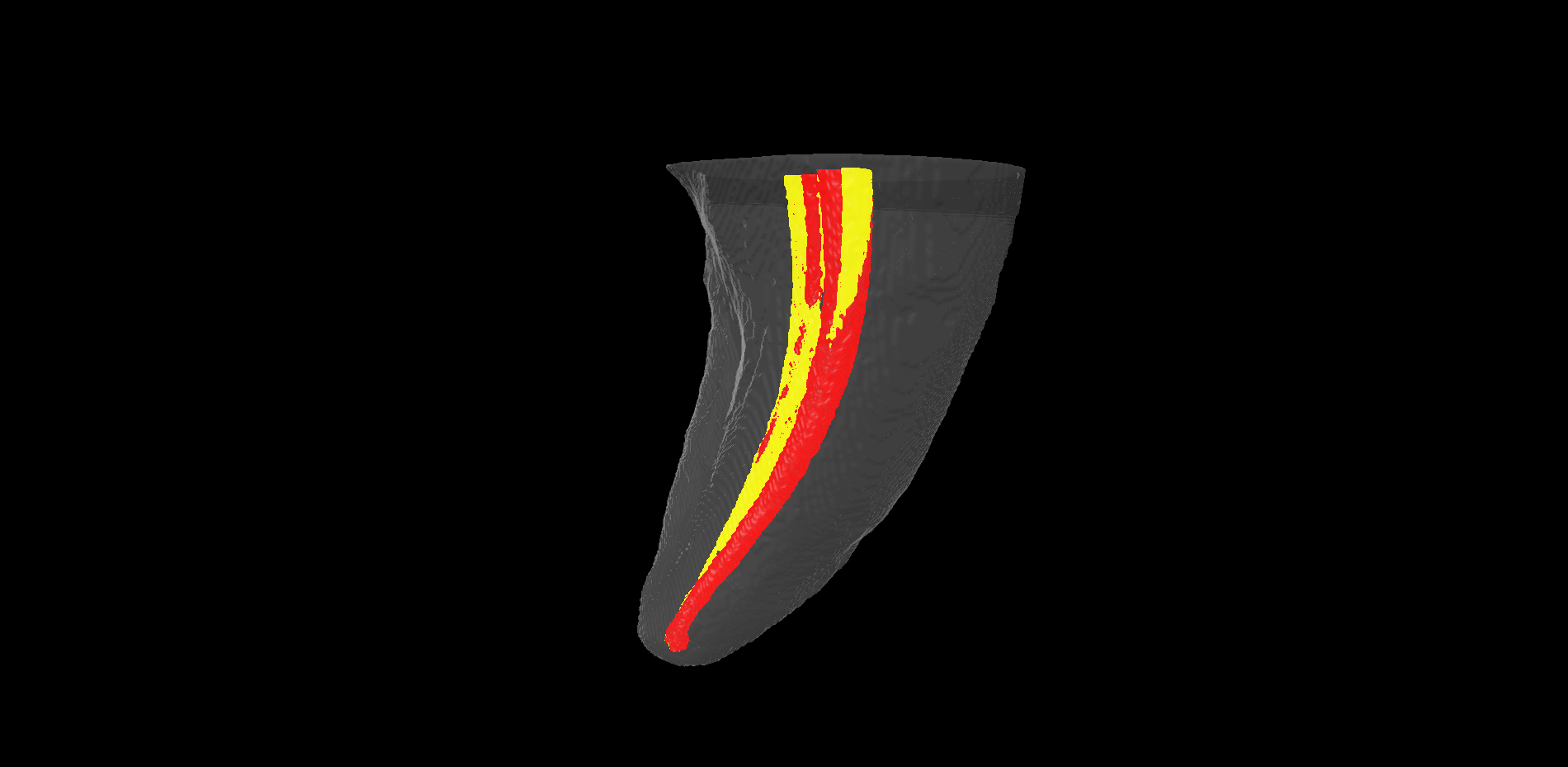

Supplement: S1 File — (ZIP) [file pone.0299896.s001.zip › Dra. Ola/Results & Images/18/18_buc_2.bmp]

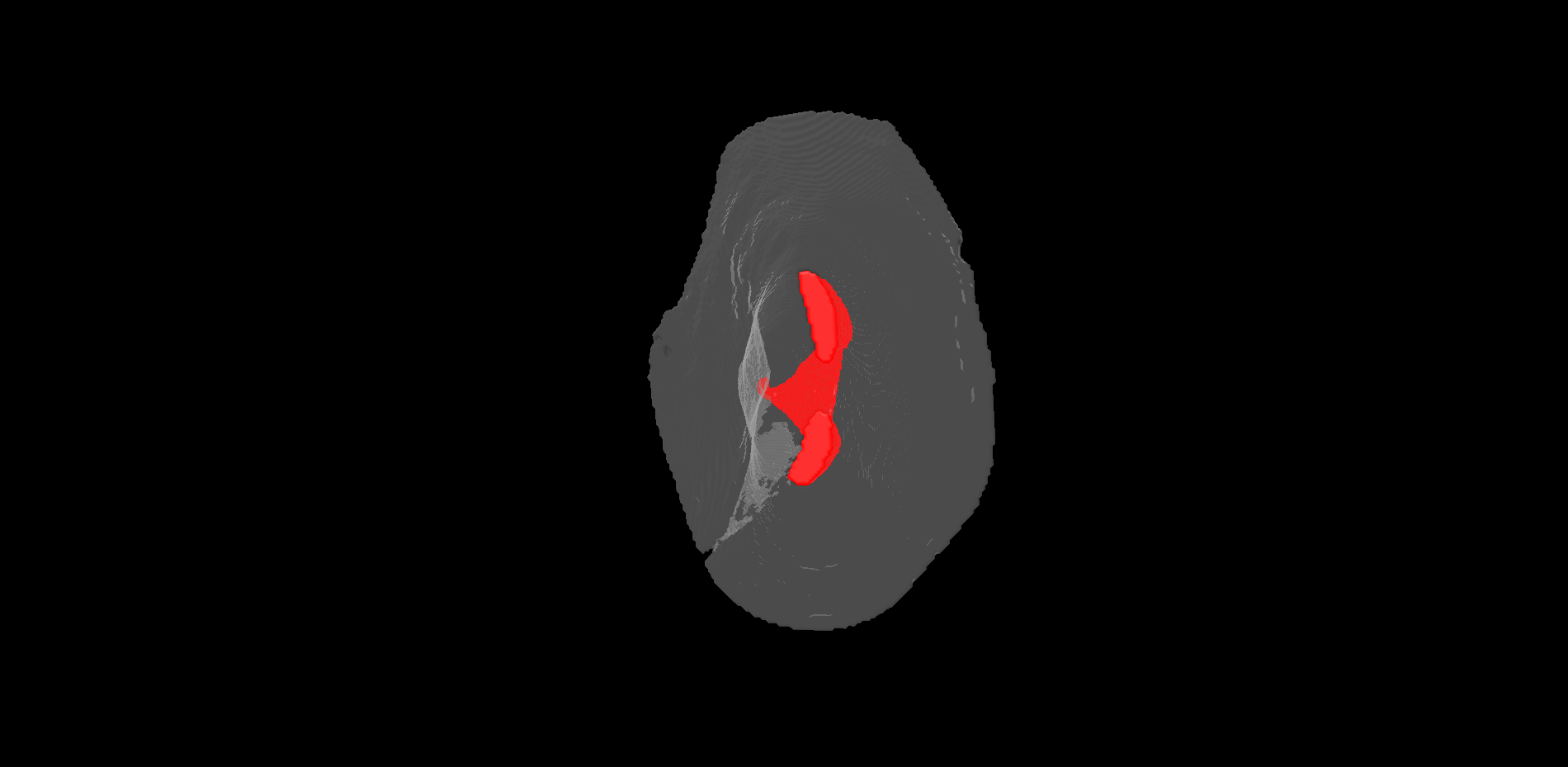

Supplement: S1 File — (ZIP) [file pone.0299896.s001.zip › Dra. Ola/Results & Images/18/18_cor.bmp]

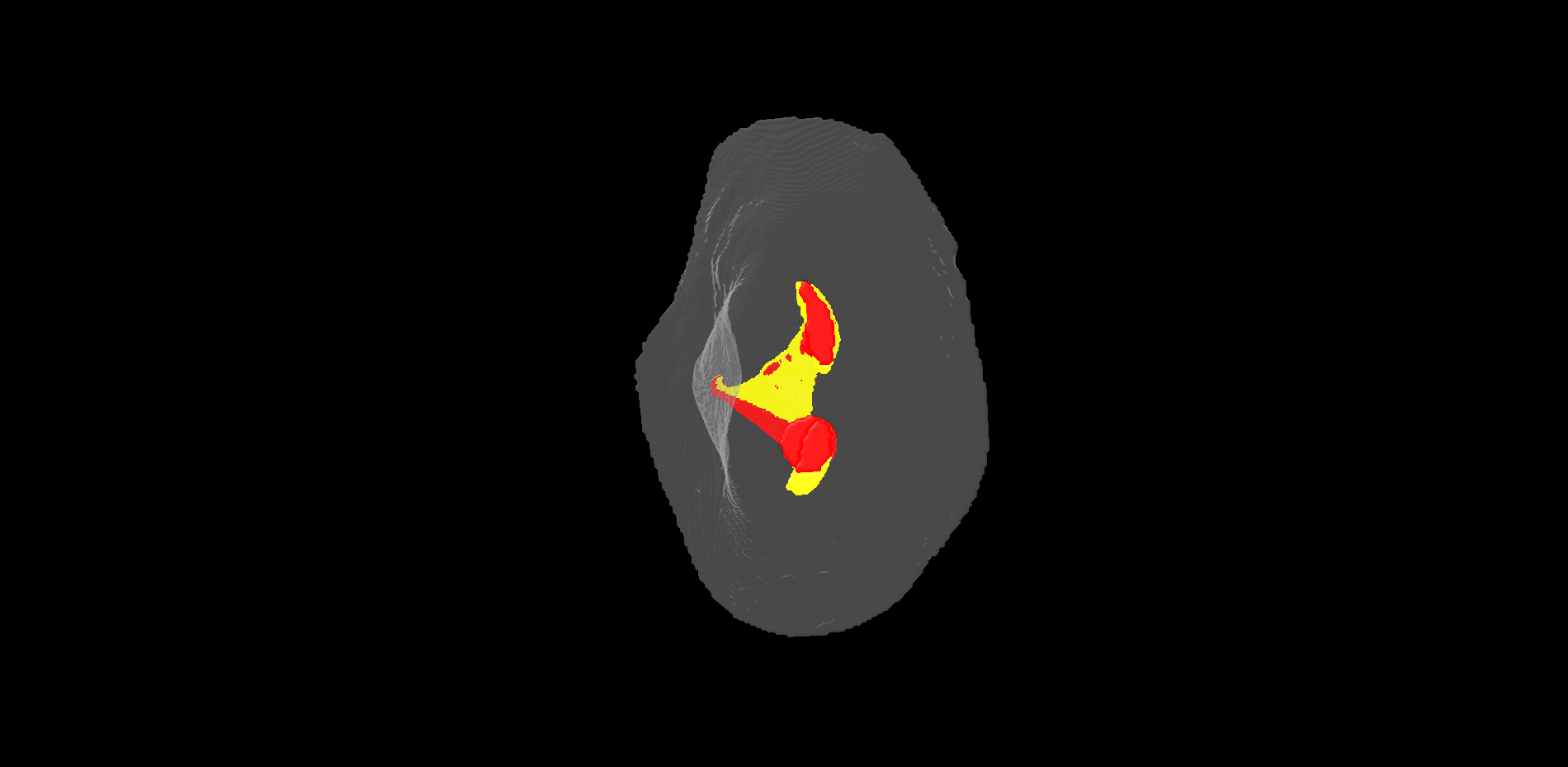

Supplement: S1 File — (ZIP) [file pone.0299896.s001.zip › Dra. Ola/Results & Images/18/18_cor_2.bmp]

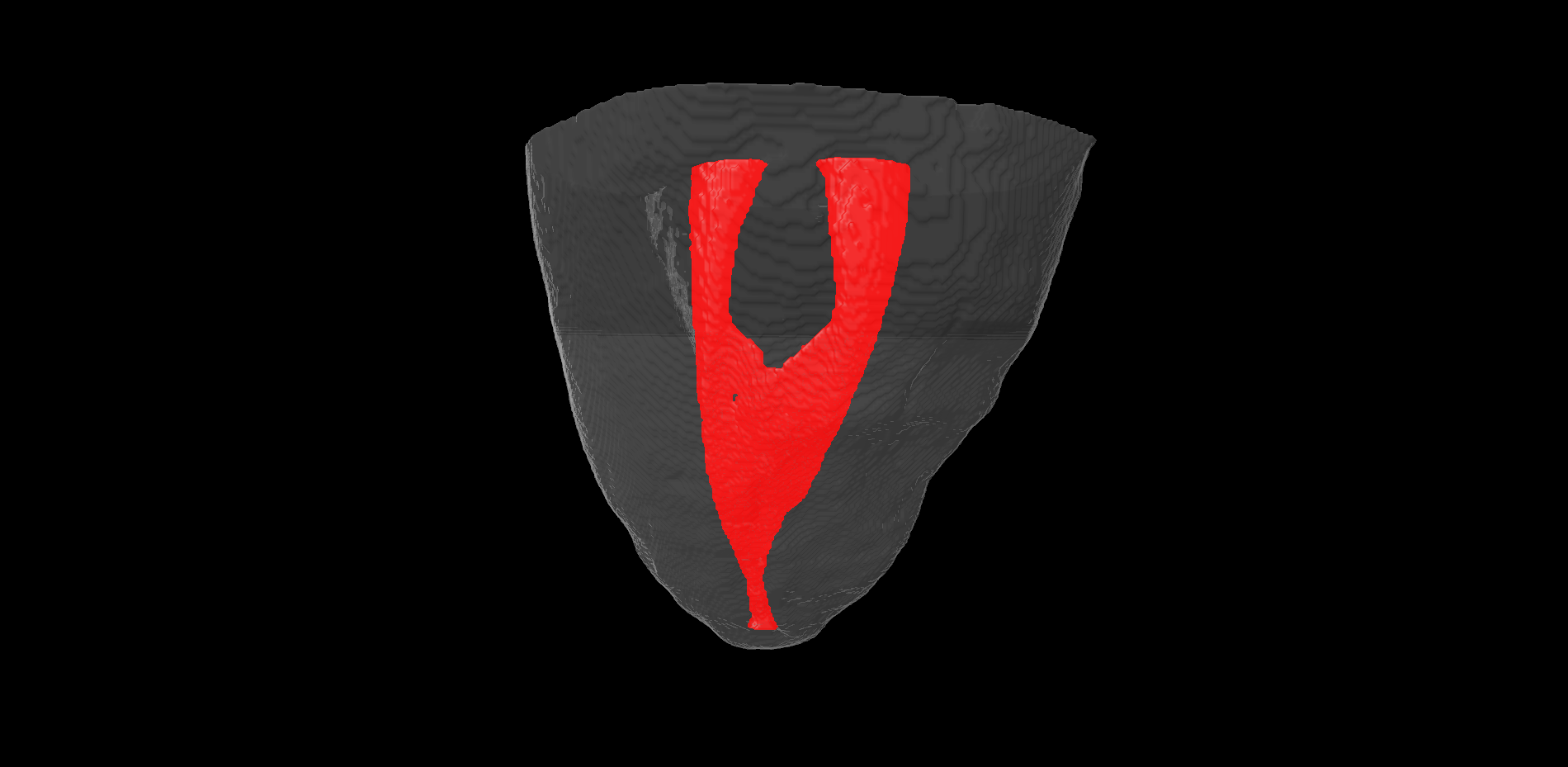

Supplement: S1 File — (ZIP) [file pone.0299896.s001.zip › Dra. Ola/Results & Images/18/18_mes.bmp]

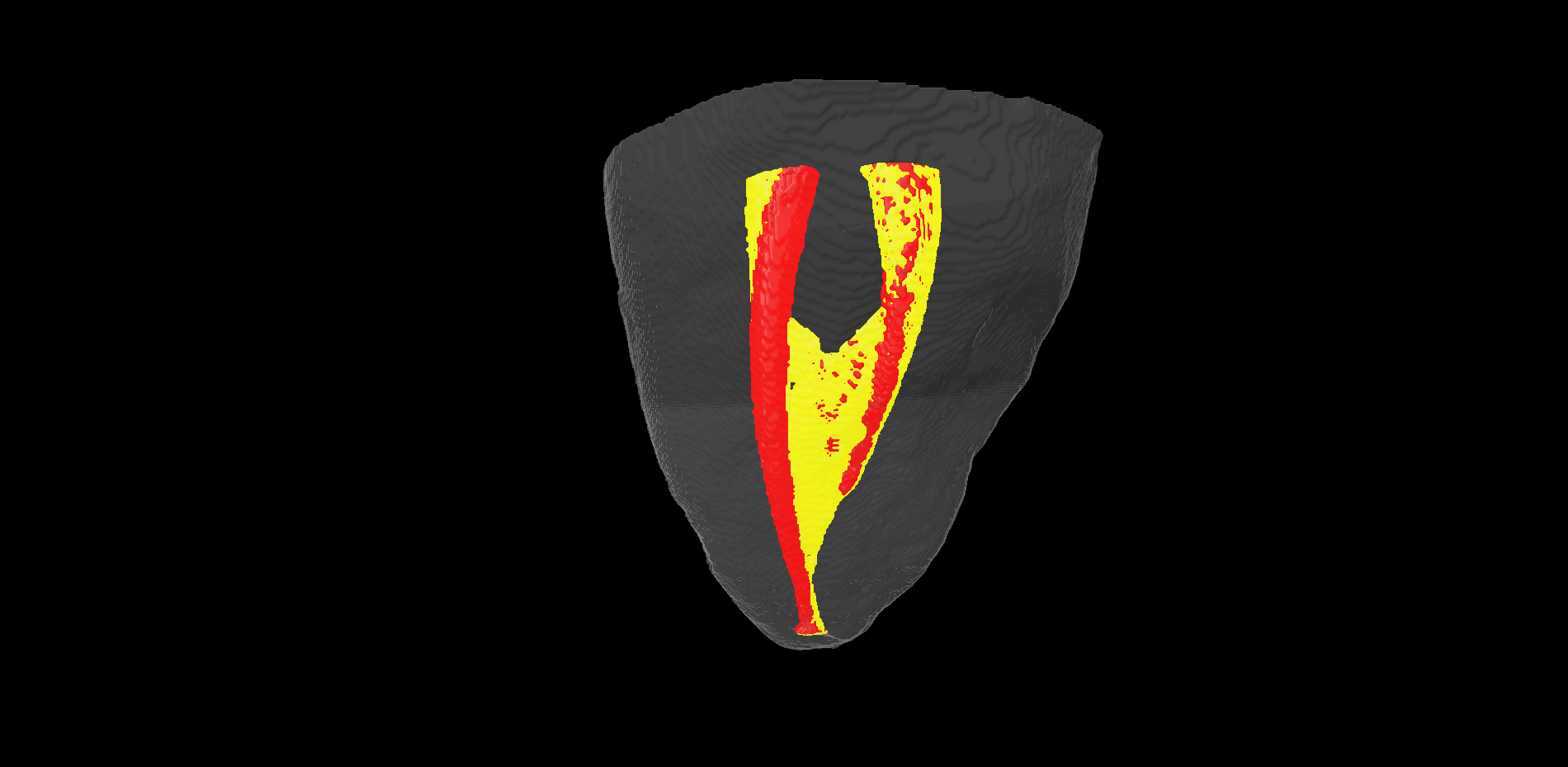

Supplement: S1 File — (ZIP) [file pone.0299896.s001.zip › Dra. Ola/Results & Images/18/18_mes_2.bmp]

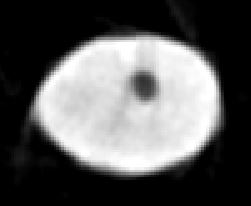

Supplement: S1 File — (ZIP) [file pone.0299896.s001.zip › Dra. Ola/Results & Images/18/1mm post.JPG]

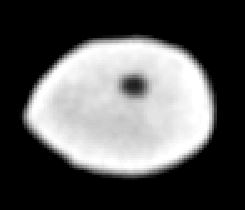

Supplement: S1 File — (ZIP) [file pone.0299896.s001.zip › Dra. Ola/Results & Images/18/1mm pre.JPG]

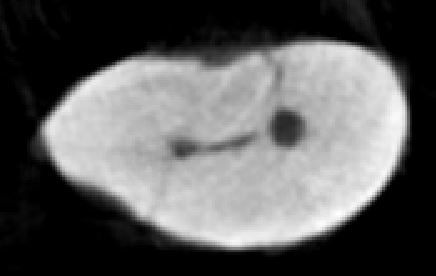

Supplement: S1 File — (ZIP) [file pone.0299896.s001.zip › Dra. Ola/Results & Images/18/3mm post.JPG]

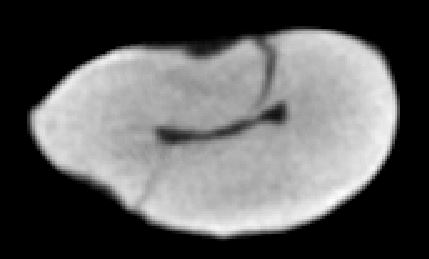

Supplement: S1 File — (ZIP) [file pone.0299896.s001.zip › Dra. Ola/Results & Images/18/3mm pre.JPG]

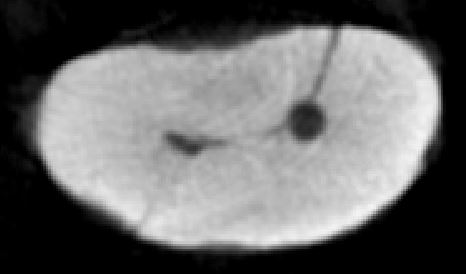

Supplement: S1 File — (ZIP) [file pone.0299896.s001.zip › Dra. Ola/Results & Images/18/5mm post.JPG]

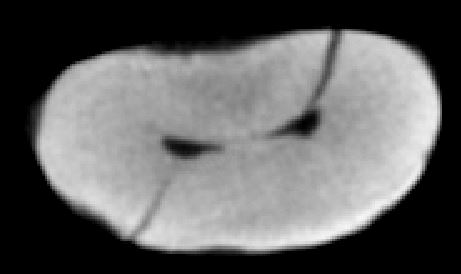

Supplement: S1 File — (ZIP) [file pone.0299896.s001.zip › Dra. Ola/Results & Images/18/5mm pre.JPG]

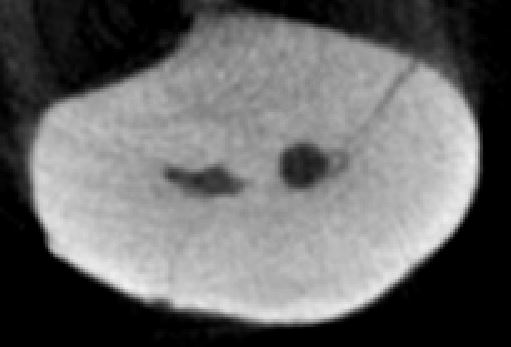

Supplement: S1 File — (ZIP) [file pone.0299896.s001.zip › Dra. Ola/Results & Images/18/7mm post.JPG]

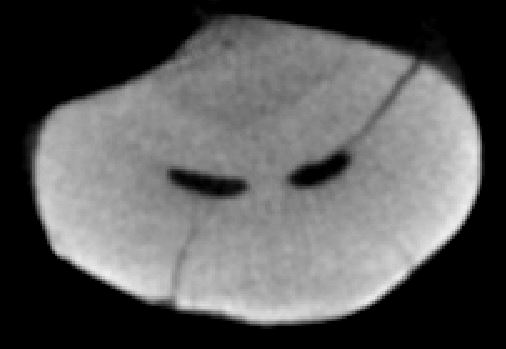

Supplement: S1 File — (ZIP) [file pone.0299896.s001.zip › Dra. Ola/Results & Images/18/7mm pre.JPG]

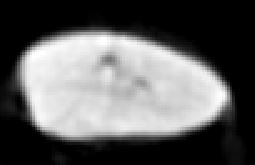

Supplement: S1 File — (ZIP) [file pone.0299896.s001.zip › Dra. Ola/Results & Images/19/1mm post.JPG]

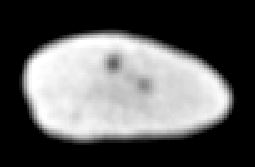

Supplement: S1 File — (ZIP) [file pone.0299896.s001.zip › Dra. Ola/Results & Images/19/1mm pre.JPG]

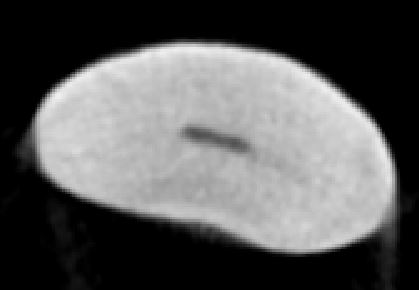

Supplement: S1 File — (ZIP) [file pone.0299896.s001.zip › Dra. Ola/Results & Images/19/3mm post.JPG]

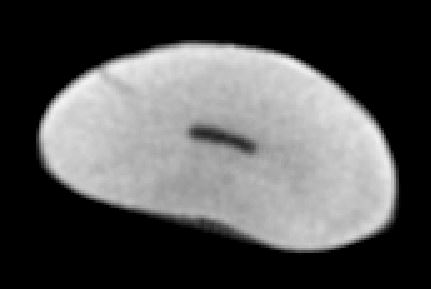

Supplement: S1 File — (ZIP) [file pone.0299896.s001.zip › Dra. Ola/Results & Images/19/3mm pre.JPG]

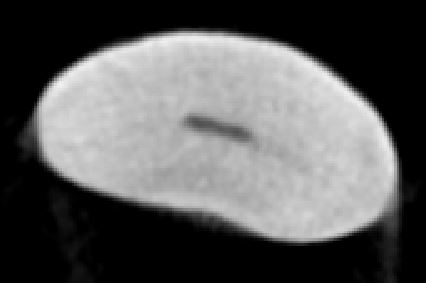

Supplement: S1 File — (ZIP) [file pone.0299896.s001.zip › Dra. Ola/Results & Images/19/5mm post.JPG]

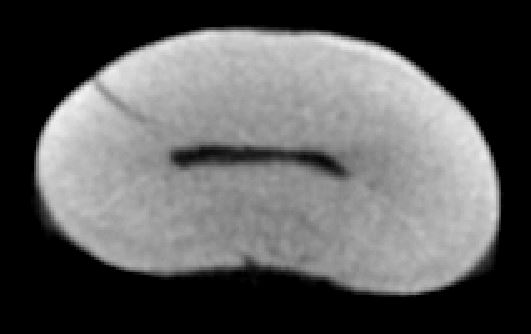

Supplement: S1 File — (ZIP) [file pone.0299896.s001.zip › Dra. Ola/Results & Images/19/5mm pre.JPG]

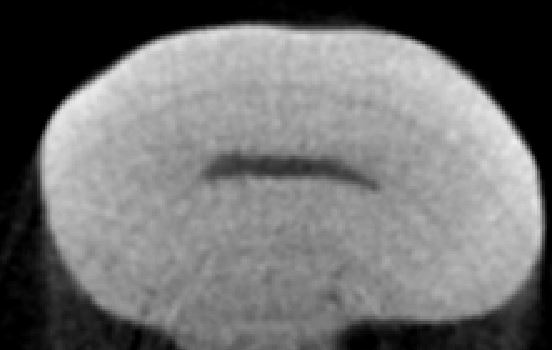

Supplement: S1 File — (ZIP) [file pone.0299896.s001.zip › Dra. Ola/Results & Images/19/7mm post.JPG]

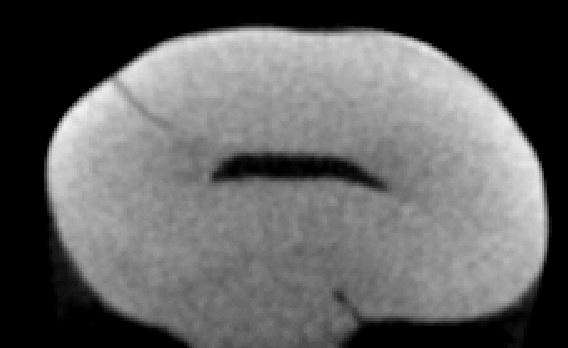

Supplement: S1 File — (ZIP) [file pone.0299896.s001.zip › Dra. Ola/Results & Images/19/7mm pre.JPG]

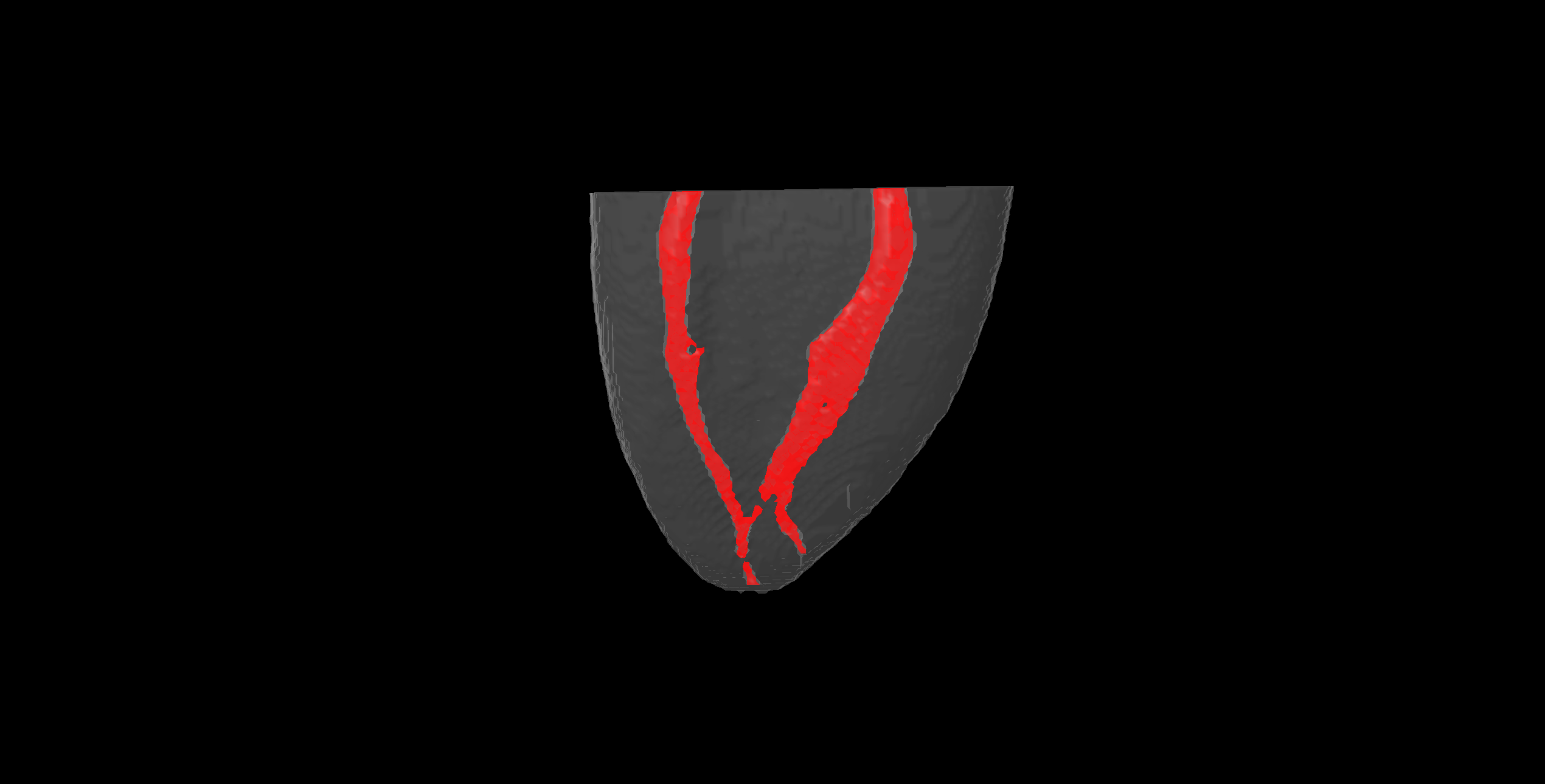

Supplement: S1 File — (ZIP) [file pone.0299896.s001.zip › Dra. Ola/Results & Images/2/1.bmp]

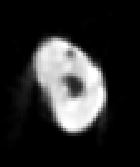

Supplement: S1 File — (ZIP) [file pone.0299896.s001.zip › Dra. Ola/Results & Images/2/1mm post.JPG]

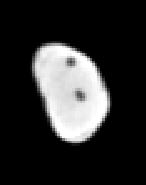

Supplement: S1 File — (ZIP) [file pone.0299896.s001.zip › Dra. Ola/Results & Images/2/1mm pre.JPG]

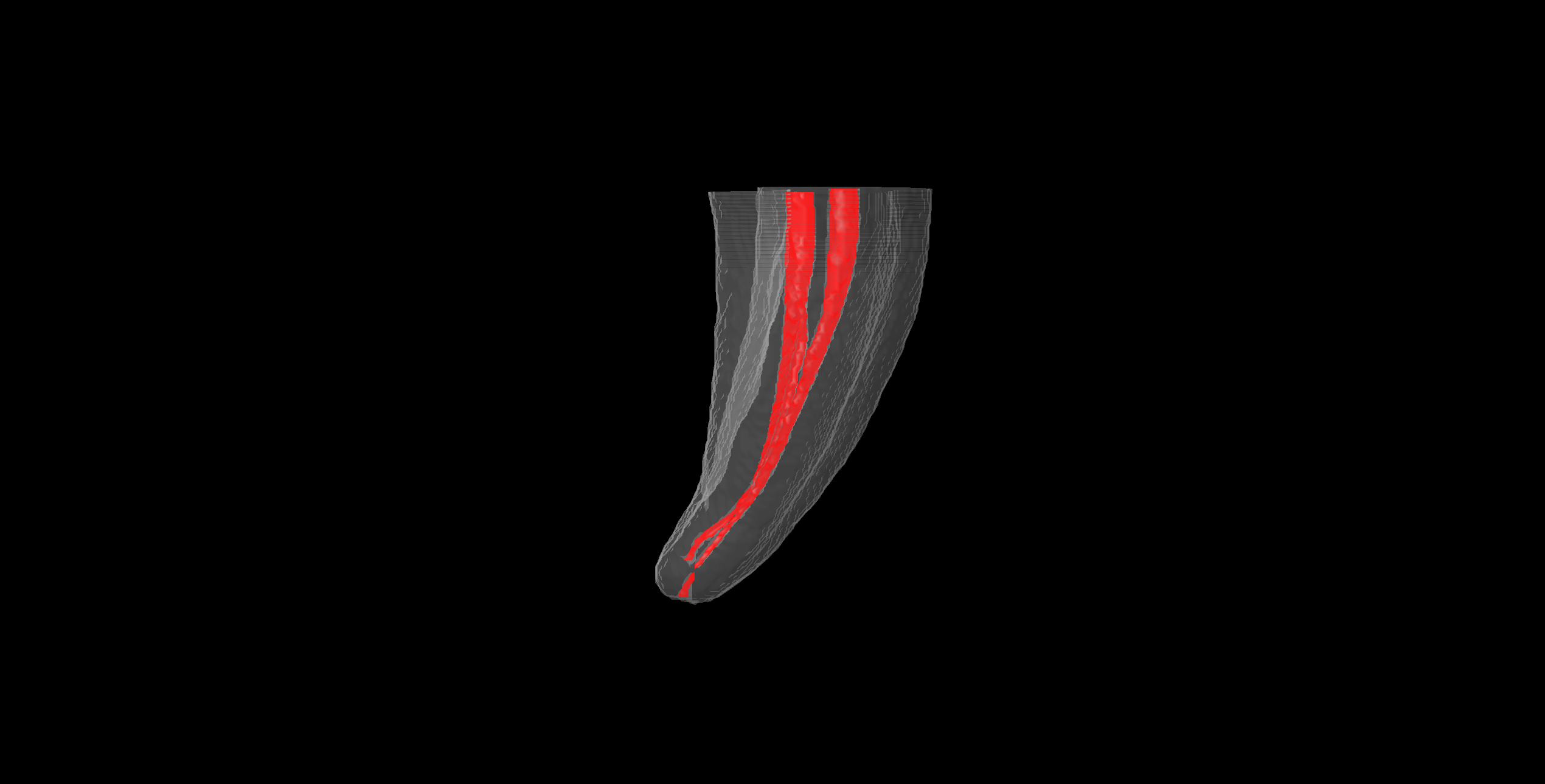

Supplement: S1 File — (ZIP) [file pone.0299896.s001.zip › Dra. Ola/Results & Images/2/2.bmp]

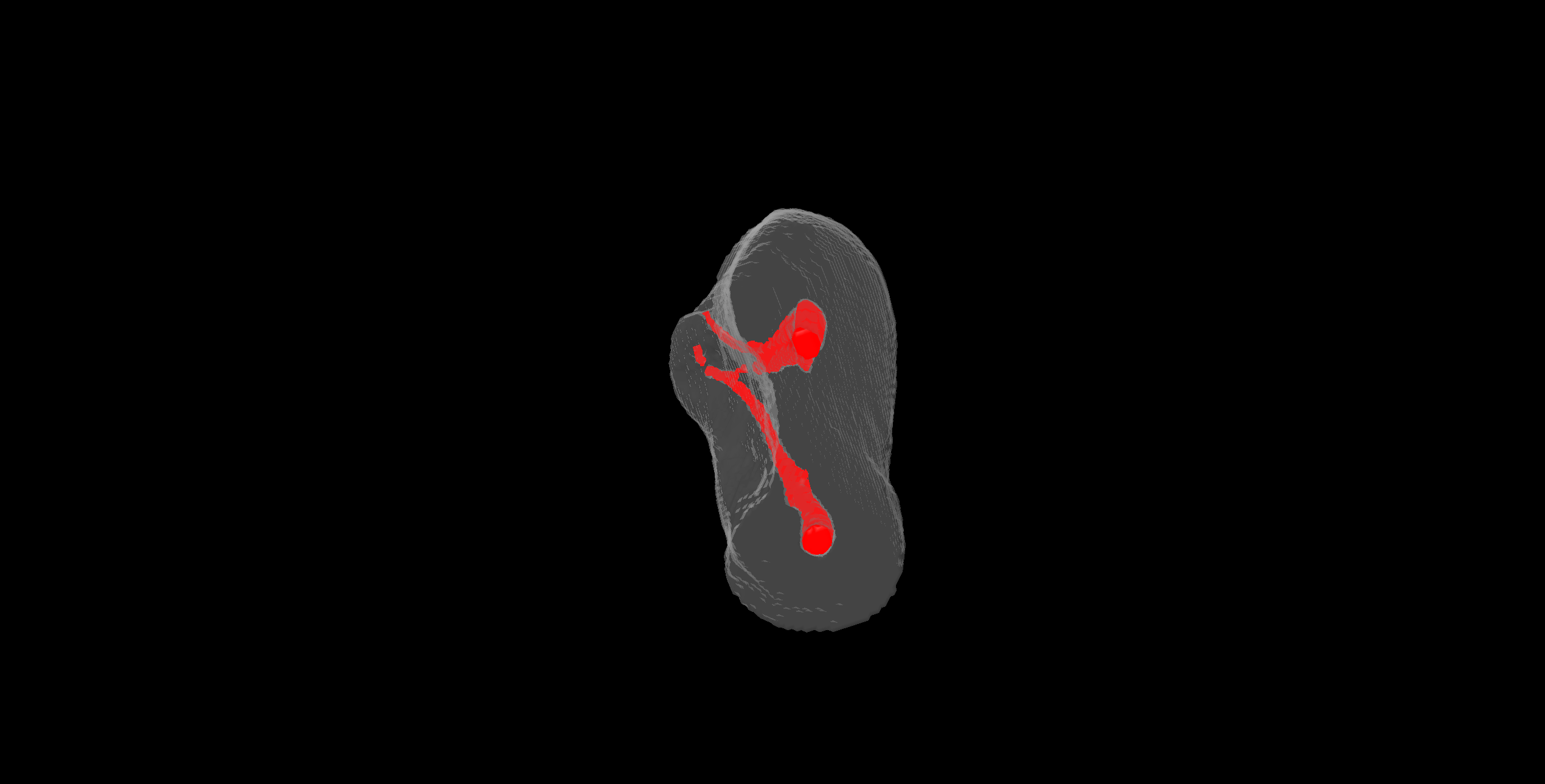

Supplement: S1 File — (ZIP) [file pone.0299896.s001.zip › Dra. Ola/Results & Images/2/3.bmp]

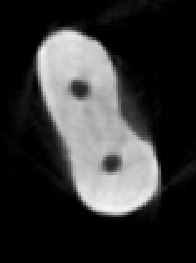

Supplement: S1 File — (ZIP) [file pone.0299896.s001.zip › Dra. Ola/Results & Images/2/3mm post.JPG]

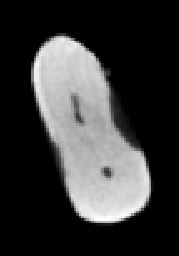

Supplement: S1 File — (ZIP) [file pone.0299896.s001.zip › Dra. Ola/Results & Images/2/3mm pre.JPG]

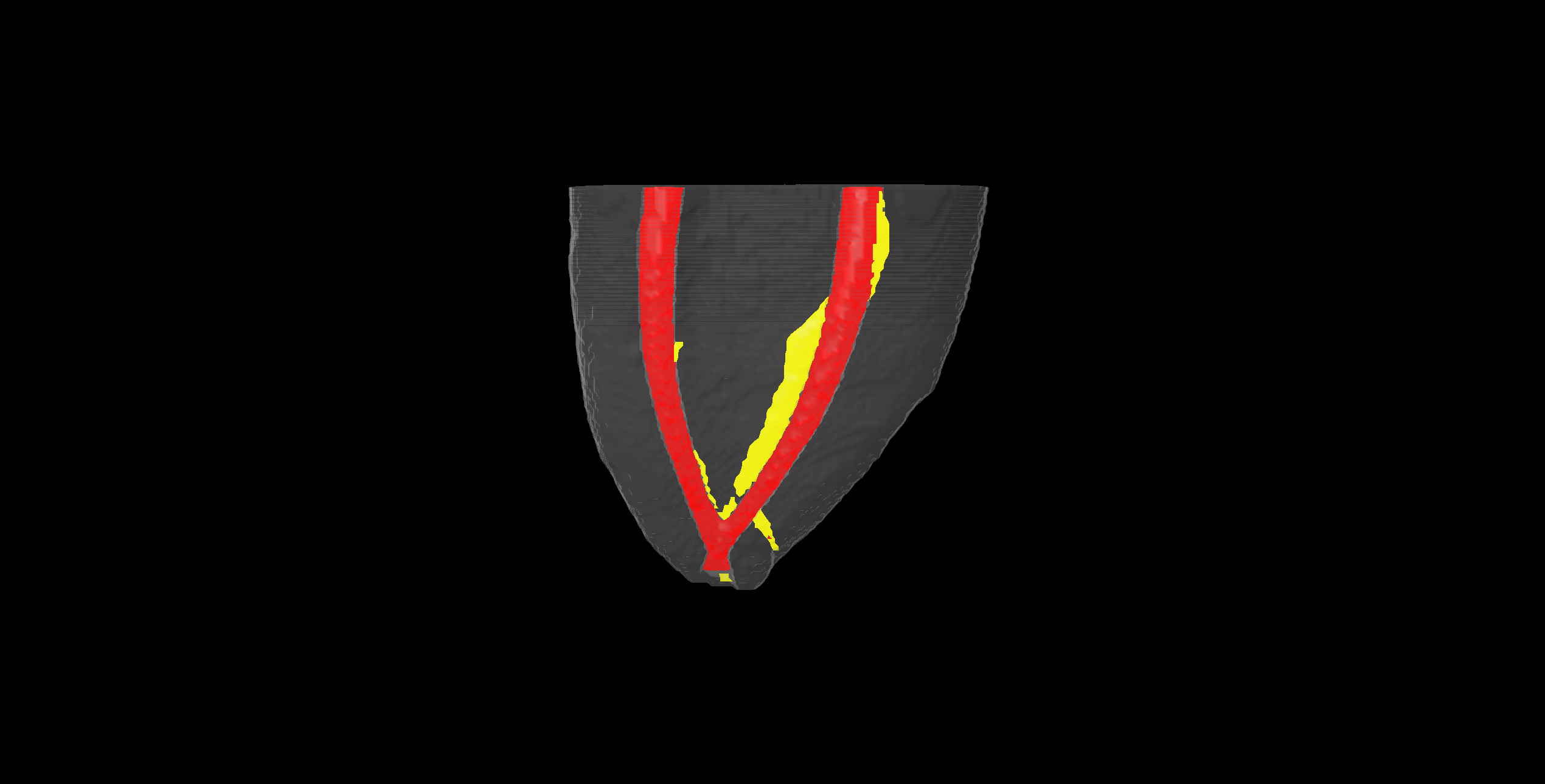

Supplement: S1 File — (ZIP) [file pone.0299896.s001.zip › Dra. Ola/Results & Images/2/4.bmp]

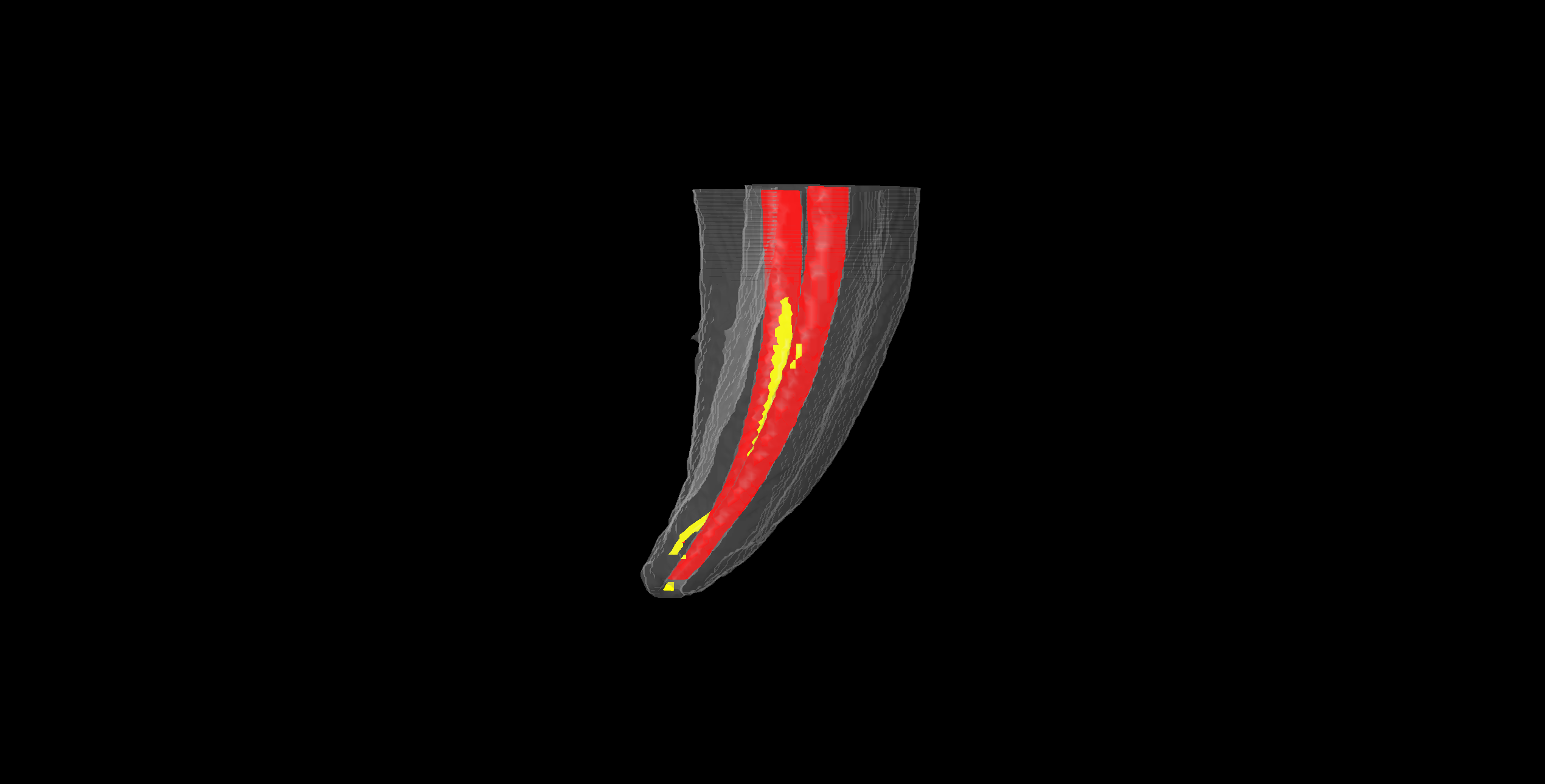

Supplement: S1 File — (ZIP) [file pone.0299896.s001.zip › Dra. Ola/Results & Images/2/5.bmp]

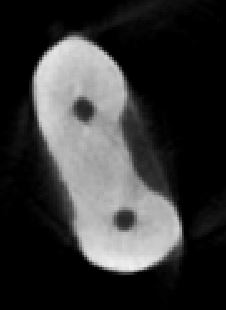

Supplement: S1 File — (ZIP) [file pone.0299896.s001.zip › Dra. Ola/Results & Images/2/5mm post.JPG]

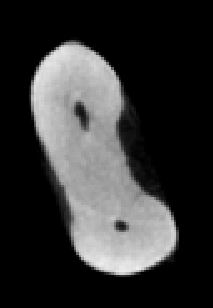

Supplement: S1 File — (ZIP) [file pone.0299896.s001.zip › Dra. Ola/Results & Images/2/5mm pre.JPG]

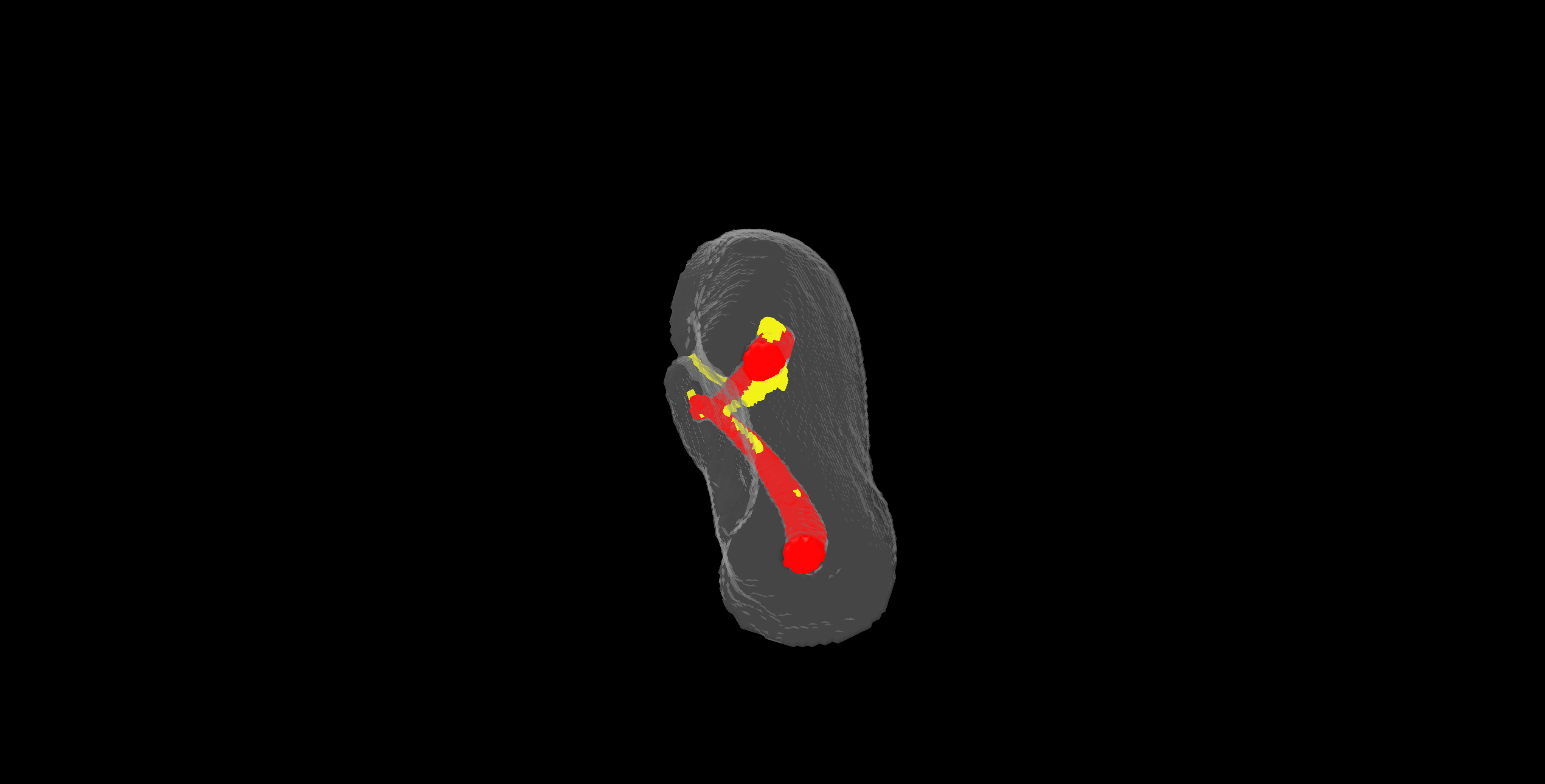

Supplement: S1 File — (ZIP) [file pone.0299896.s001.zip › Dra. Ola/Results & Images/2/6.bmp]

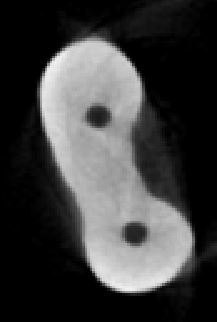

Supplement: S1 File — (ZIP) [file pone.0299896.s001.zip › Dra. Ola/Results & Images/2/7mm post.JPG]

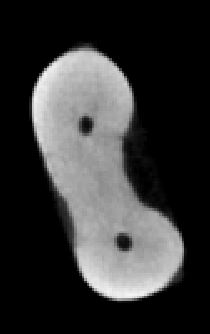

Supplement: S1 File — (ZIP) [file pone.0299896.s001.zip › Dra. Ola/Results & Images/2/7mm pre.JPG]

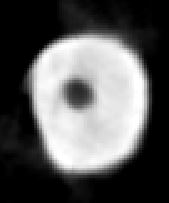

Supplement: S1 File — (ZIP) [file pone.0299896.s001.zip › Dra. Ola/Results & Images/20/1mm post.JPG]

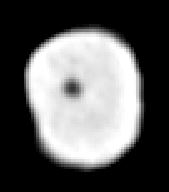

Supplement: S1 File — (ZIP) [file pone.0299896.s001.zip › Dra. Ola/Results & Images/20/1mm pre.JPG]

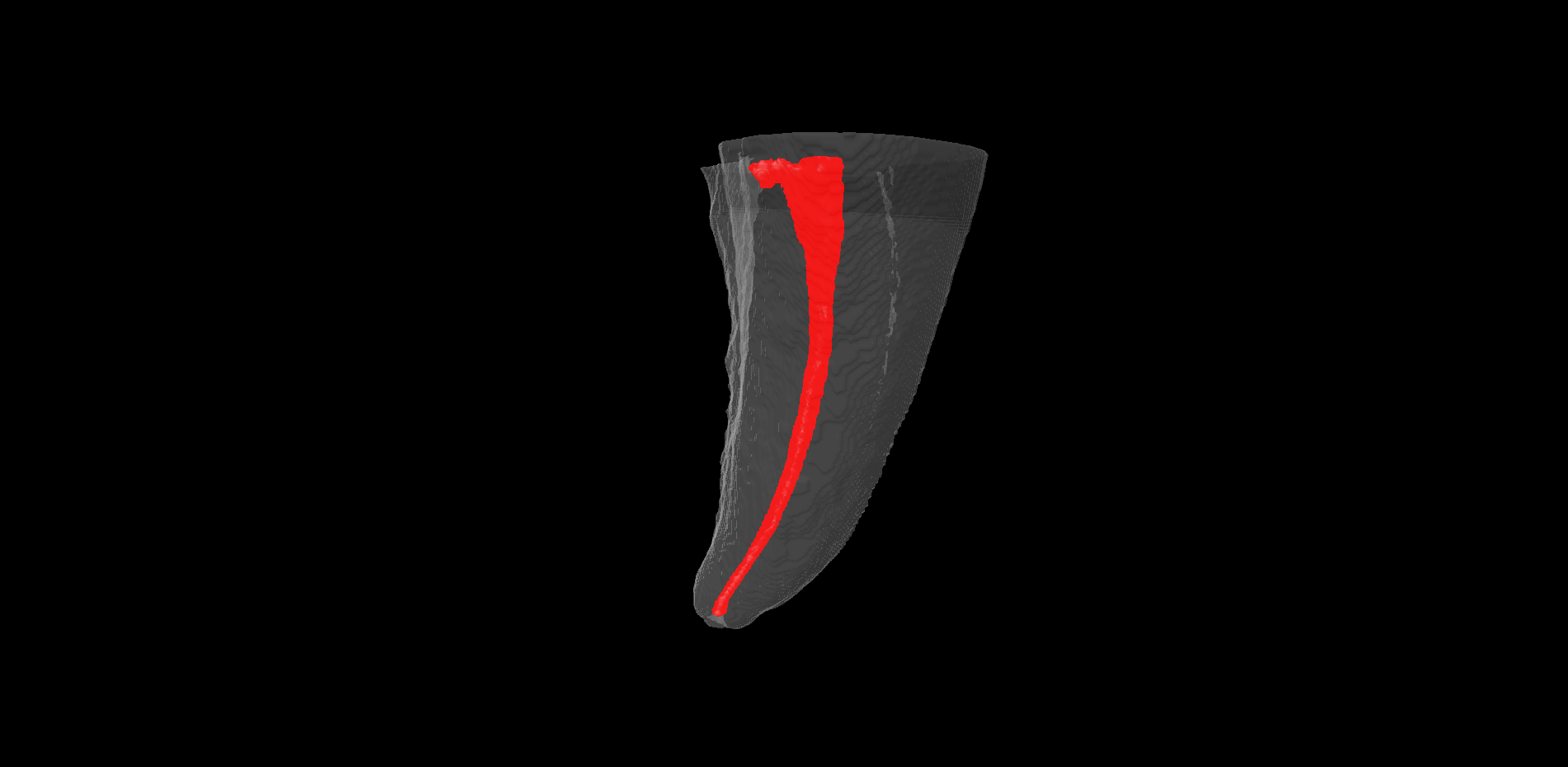

Supplement: S1 File — (ZIP) [file pone.0299896.s001.zip › Dra. Ola/Results & Images/20/20_buc.bmp]

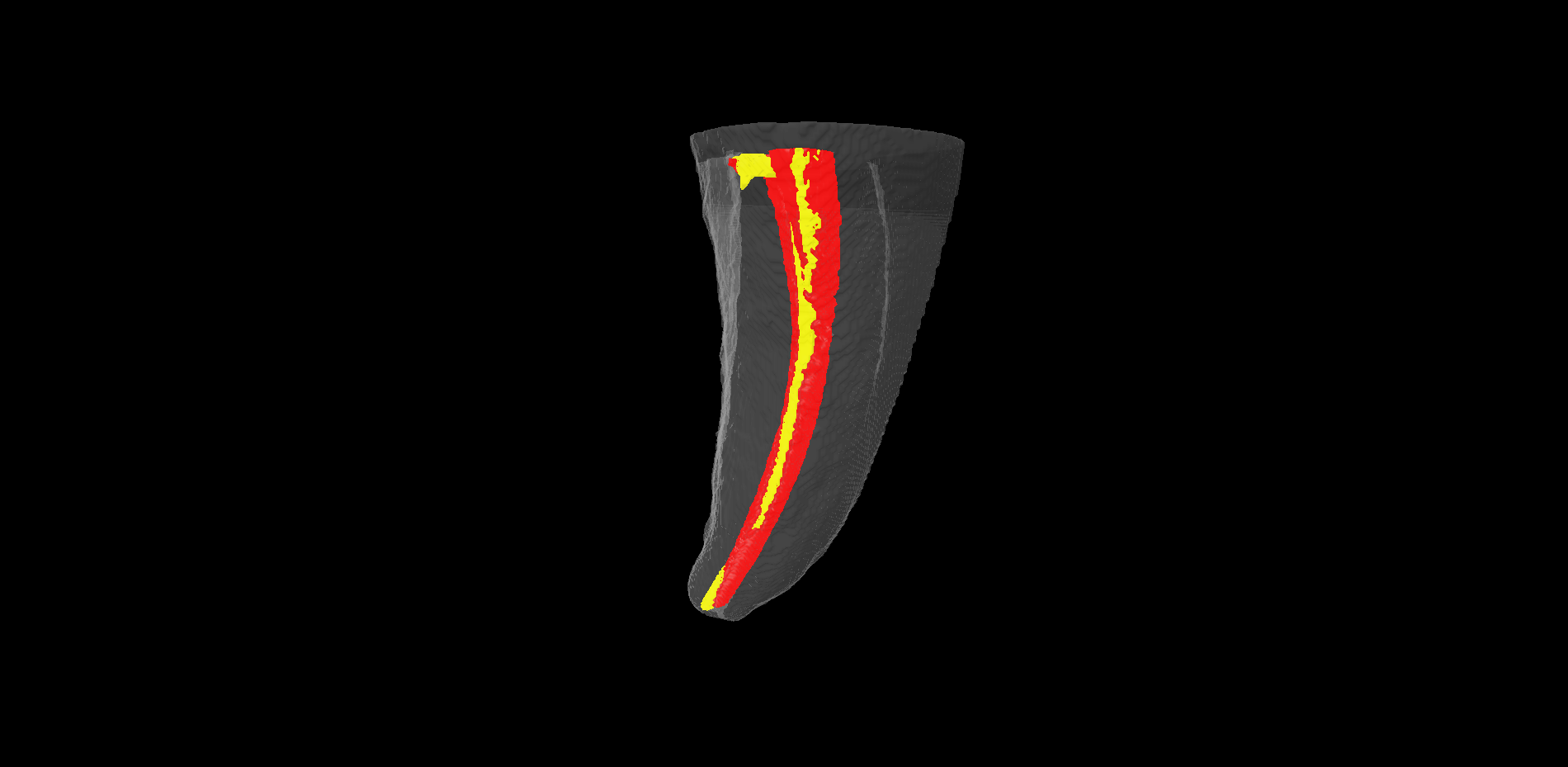

Supplement: S1 File — (ZIP) [file pone.0299896.s001.zip › Dra. Ola/Results & Images/20/20_buc_2.bmp]

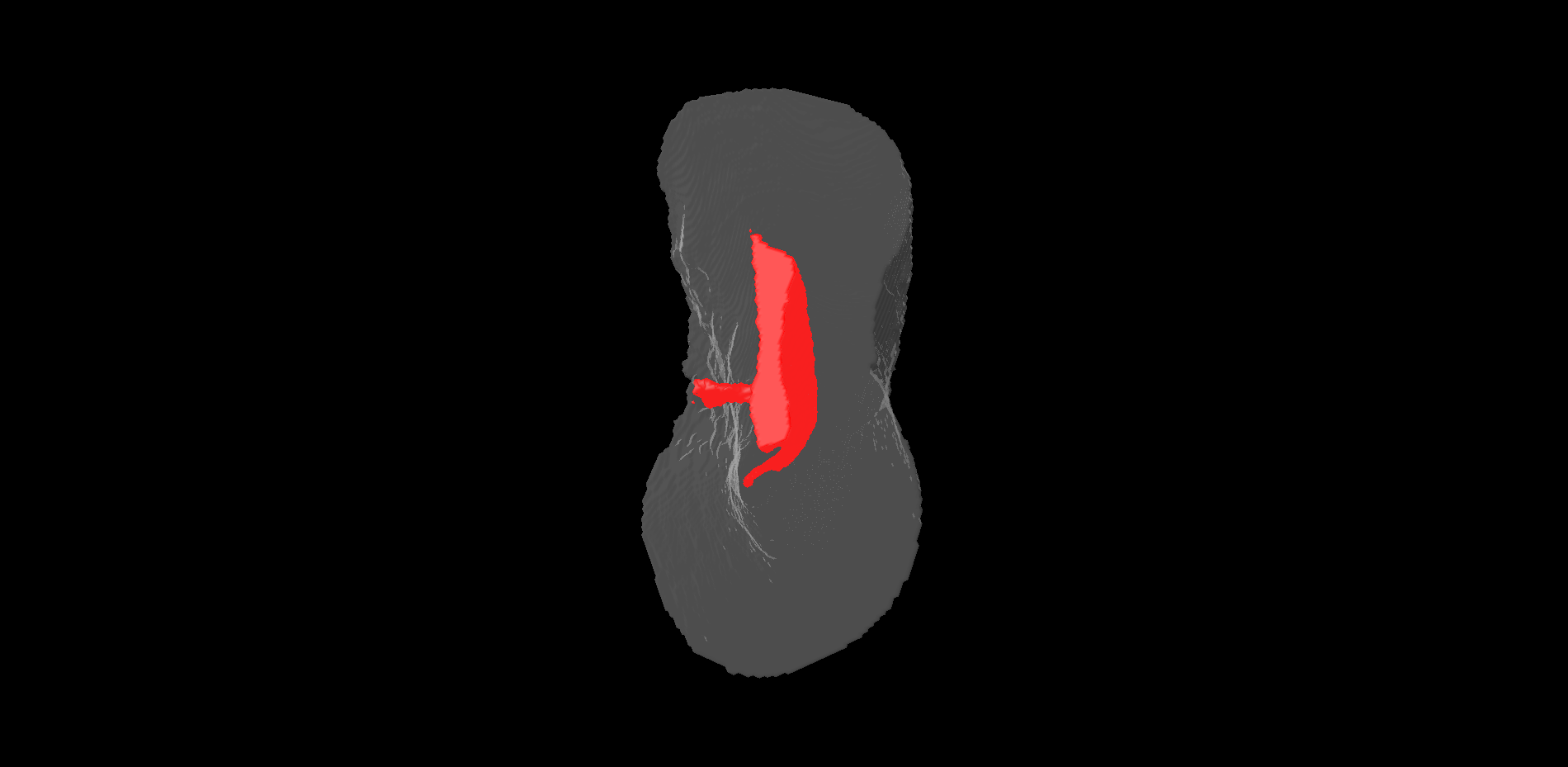

Supplement: S1 File — (ZIP) [file pone.0299896.s001.zip › Dra. Ola/Results & Images/20/20_cor.bmp]

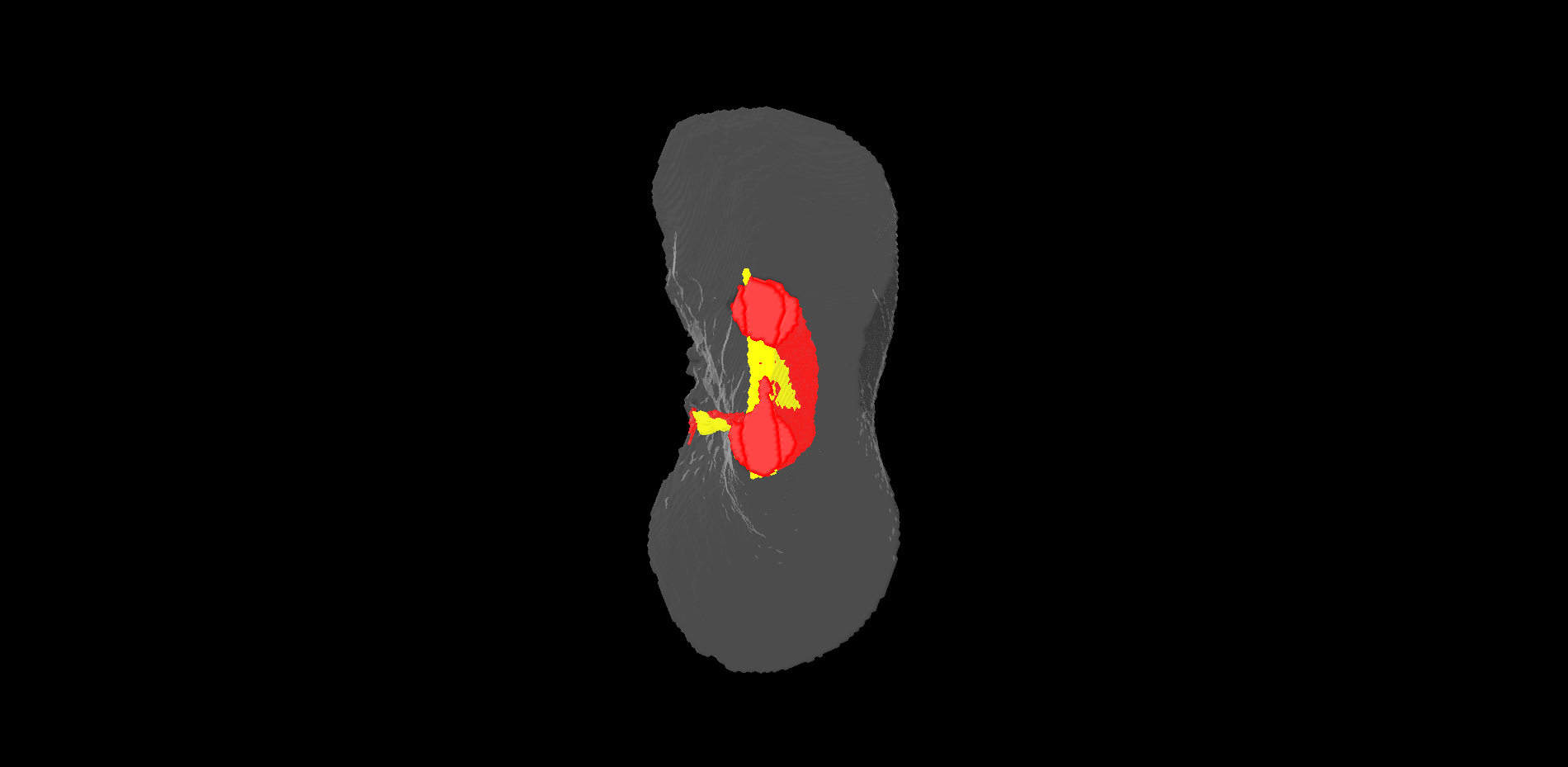

Supplement: S1 File — (ZIP) [file pone.0299896.s001.zip › Dra. Ola/Results & Images/20/20_cor_2.bmp]

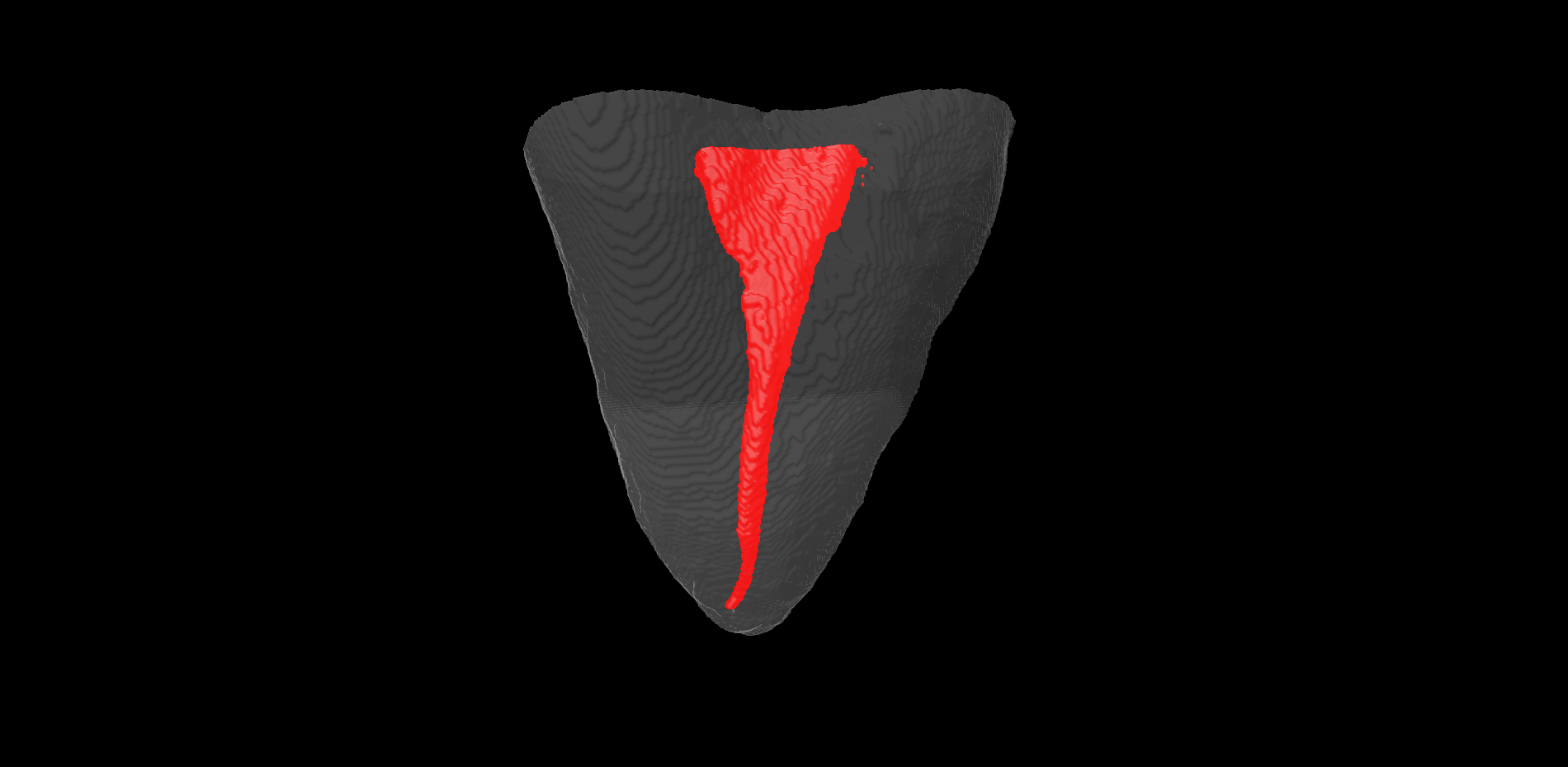

Supplement: S1 File — (ZIP) [file pone.0299896.s001.zip › Dra. Ola/Results & Images/20/20_mes.bmp]

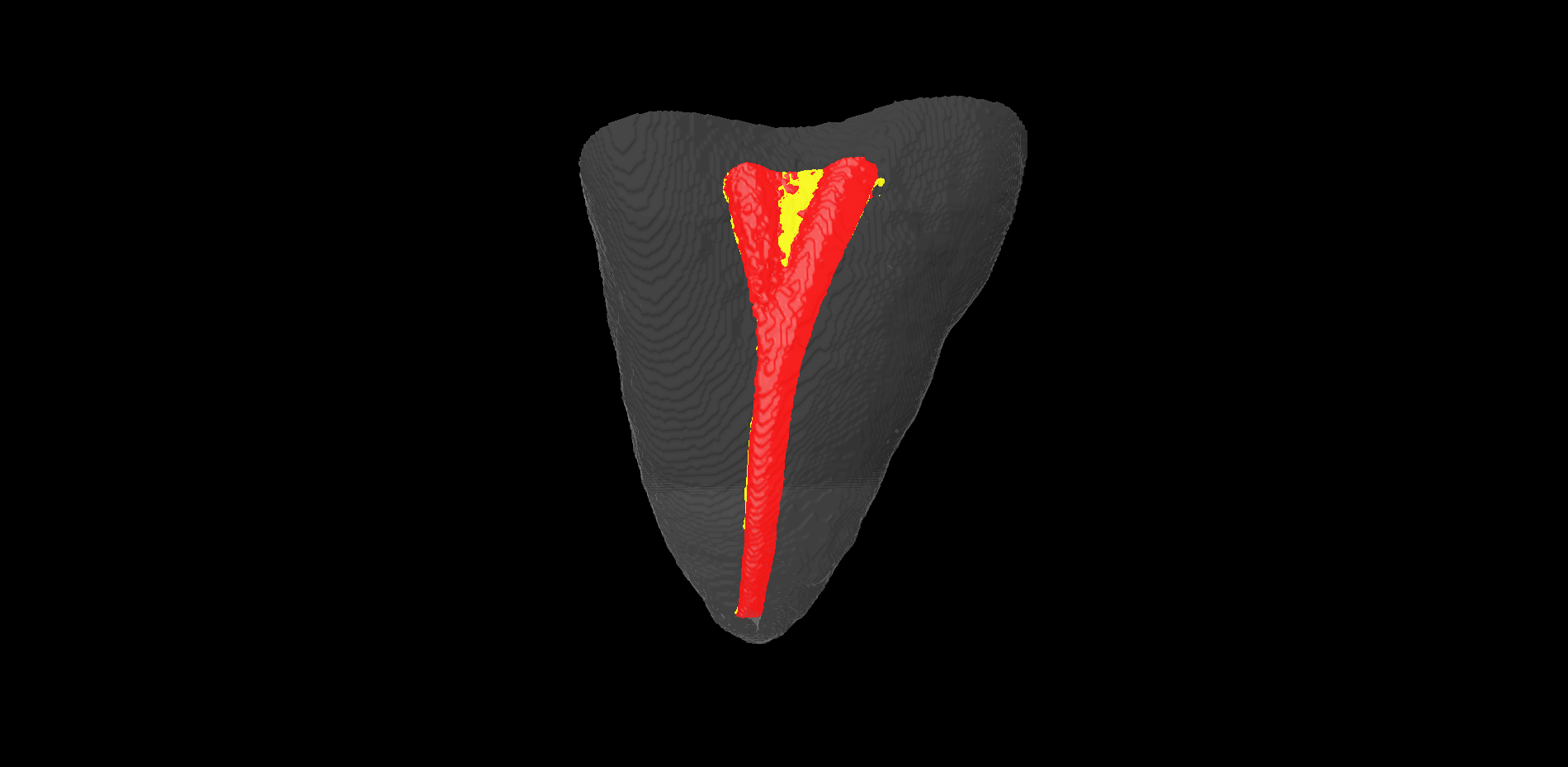

Supplement: S1 File — (ZIP) [file pone.0299896.s001.zip › Dra. Ola/Results & Images/20/20_mes_2.bmp]

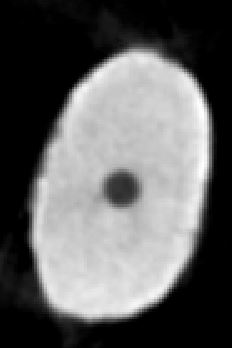

Supplement: S1 File — (ZIP) [file pone.0299896.s001.zip › Dra. Ola/Results & Images/20/3mm post.JPG]

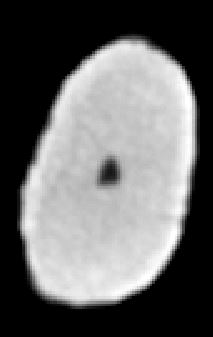

Supplement: S1 File — (ZIP) [file pone.0299896.s001.zip › Dra. Ola/Results & Images/20/3mm pre.JPG]

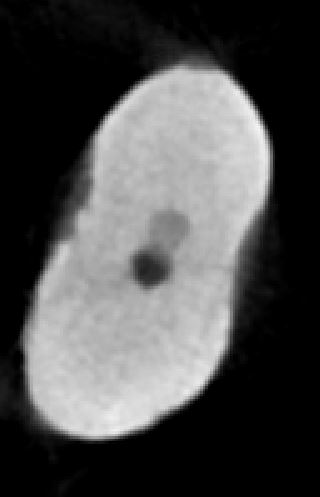

Supplement: S1 File — (ZIP) [file pone.0299896.s001.zip › Dra. Ola/Results & Images/20/5mm post.JPG]

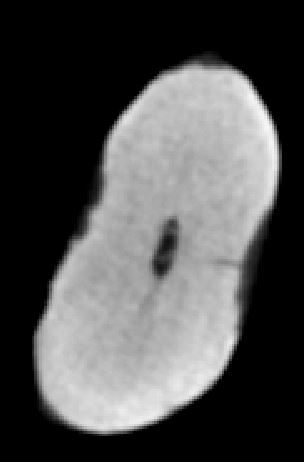

Supplement: S1 File — (ZIP) [file pone.0299896.s001.zip › Dra. Ola/Results & Images/20/5mm pre.JPG]

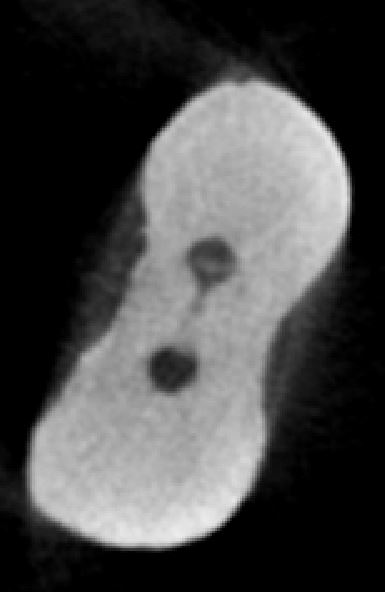

Supplement: S1 File — (ZIP) [file pone.0299896.s001.zip › Dra. Ola/Results & Images/20/7mm post.JPG]

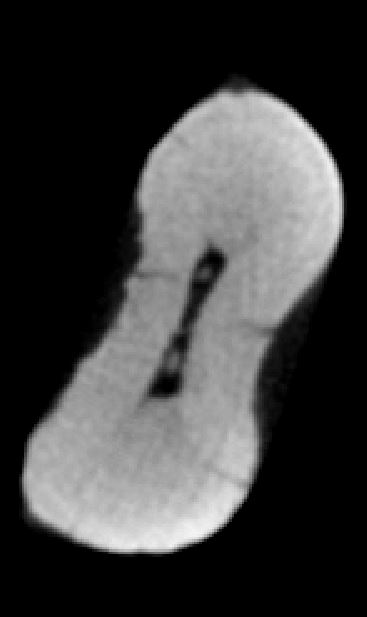

Supplement: S1 File — (ZIP) [file pone.0299896.s001.zip › Dra. Ola/Results & Images/20/7mm pre.JPG]
